# Long-term hazard of recurrence in HER2+ breast cancer patients untreated with anti-HER2 therapy

**DOI:** 10.1186/s13058-015-0568-1

**Published:** 2015-04-16

**Authors:** Kathrin Strasser-Weippl, Nora Horick, Ian E Smith, Joyce O’Shaughnessy, Bent Ejlertsen, Frances Boyle, Aman U Buzdar, Pierre Fumoleau, William Gradishar, Miguel Martin, Beverly Moy, Martine Piccart-Gebhart, Kathleen I Pritchard, Deborah Lindquist, Erica Rappold, Dianne M Finkelstein, Paul E Goss

**Affiliations:** Center for Oncology and Hematology, Wilhelminen Hospital, Montleartstrasse 37, Vienna, 1160 Austria; Massachusetts General Hospital Biostatistics Center, 50 Staniford Street, Boston, MA 02478 USA; Royal Marsden Hospital, Fulham Road, London, SW3 6JJ UK; Baylor Sammons Cancer Center, Texas Oncology, US Oncology, 3410 Worth Street, Dallas, TX 75246 USA; Rigshospitalet, DBCG Secretariat, Blegdamsvej 9, DK-2100 Copenhagen, Denmark; Mater Hospital, 25 Rocklands Road, North Sydney, NSW 2060 Australia; University of Texas MD Anderson Cancer Center, 1515 Holcombe Boulevard, Houston, TX 77030 USA; Centre GF Leclerc, 1 Rue du Professeur Marion, 21000 Dijon, France; Northwestern University, 633 Clark Street, Chicago, IL 60208 USA; Hospital Universitario Gregorio Marañón, Universidad Complutense, Ciudad Universitaria, s/n, 28040 Madrid, Spain; Massachusetts General Hospital Cancer Center, 55 Fruit Street, Boston, MA 02114 USA; Institut Jules Bordet, Université Libre de Bruxelles, Boulevard de Waterloo 121, 1000 Brussels, Belgium; Sunnybrook Odette Cancer Centre, University of Toronto, 2075 Bayview Avenue, Toronto, ON M4N 3M5 Canada; Arizona Oncology, US Oncology, 3700 W. State Route 89A, Sedona, AZ 86336 USA; GlaxoSmithKline, 1250 S Collegeville Road, Collegeville, PA 19426 USA

## Abstract

**Introduction:**

Worldwide, many patients with HER2+ (human epidermal growth factor receptor 2-positive) early breast cancer (BC) do not receive adjuvant trastuzumab. Hazards of recurrence of these patients with respect to hormone receptor status of the primary tumor have not been described.

**Methods:**

Using data from 1,260 patients randomized to placebo in the adjuvant TEACH trial, we report 10-year annual hazards of recurrence in HER2+ patients not treated with anti-HER2 therapy.

**Results:**

Disease-free survival (DFS) was 75% after 5 and 61% after 10 years, respectively. Patients with HER2+ hormone receptor-positive (HR+ (hormone receptor-positive); ER+ (estrogen receptor-positive) or PR+ (progesterone receptor-positive)) disease had a significantly better DFS than patients with HER2+ HR- (ER-/PR-) disease (hazard ratio 0.72, *P* = 0.02). This difference was explainable by a significantly higher hazard of recurrence in years 1 to 5 in HER2+ HR- compared to HER2+ HR+ patients, with a mean risk of recurrence of 9%/year for HR- versus 5%/year in HR+ patients (hazard ratio 0.59, *P* = 0.002 for years 1 to 5). The high early risk of recurrence of HER2+ HR- patients declined sharply over time, so that it was similar to that seen in HER2+ HR+ patients in years 6 to 10 (hazard ratio 0.97, *P* = 0.92 for years 6 to 10).

**Conclusions:**

Our results show that outcomes in HER2+ patients with early BC not receiving anti-HER2 therapy strongly depend on HR expression. The very high early risk of relapse seen in HER2+ HR- patients is particularly relevant in health care settings with limited access to adjuvant anti-HER2 treatment. The event rates shown for subpopulations of HER2+ BC patients suggest that in resource-constrained environments patients with HER2+ HR- early BC should be prioritized for consideration of adjuvant anti-HER2 therapy.

**Electronic supplementary material:**

The online version of this article (doi:10.1186/s13058-015-0568-1) contains supplementary material, which is available to authorized users.

## Introduction

Standard adjuvant treatment of localized high-risk human epidermal growth factor receptor 2-positive (HER2+) early breast cancer (BC) substantially reduces the risk of disease recurrence and improves survival [1]. Current standard systemic treatment consists of chemotherapy with one year of trastuzumab, a monoclonal antibody targeting the HER2 extracellular domain. In resource-limited countries or populations, access to trastuzumab is often limited by economic or logistic conditions [2].

Cancer outcomes in this common setting of having received chemotherapy +/− endocrine therapy but not trastuzumab have not been fully described.

We report here the annual hazards of recurrence based on hormone receptor (HR) status among patients with HER2+ early BC treated with chemotherapy +/− endocrine therapy but not with anti-HER2 therapy.

## Methods

### Study design and participants

Our analyses are based on data from the placebo arm of the adjuvant Tykerb evaluation after chemotherapy (TEACH) trial (ClinicalTrials.gov number NCT00374322), the design and main outcomes of which have been previously published [3]. Briefly, between August 2006 and May 2008, the study recruited 3,161 women with stage I to IIIC (node-negative patients were enrolled only if tumors were ≥1 cm) HER2+ invasive BC who were disease-free at any time in follow-up after completion of prior adjuvant chemotherapy, but who had not received adjuvant trastuzumab. By randomizing patients at any time after diagnosis, the design of this trial and the resulting cohort of patients included are unique, as patients as late as 179 months from diagnosis were randomized (analysis accounts for left-truncation), resulting in a very long follow-up period from diagnosis.

The study was approved by ethics review boards at all participating centers (see Additional file 1). Patients gave written, informed consent. The study complied with the Declaration of Helsinki and Good Clinical Practice. Only patients assigned to the placebo group and with centrally confirmed (positive by fluorescence *in situ* hybridization) HER2+ disease (n = 1,260 of 1,576 in the placebo arm) are included in this analysis.

Patients were randomized to oral lapatinib or placebo, daily for 12 months or until disease recurrence, development of a second primary cancer, or unacceptable toxicity. Details of randomization and stratification have been previously published [3].

### Procedures

Patients were followed every 3 months up until 2 years from diagnosis if they had been randomized within 2 years from diagnosis, once every 6 months up until 5 years from diagnosis if they had been randomized between 2 and 5 years from diagnosis and yearly once they were >5 years from diagnosis. The disease-free survival (DFS) events in this analysis were defined as in the primary analysis [3].

### Statistical analysis

DFS is measured from the date of diagnosis (not the date of randomization). Patients on TEACH had to have had a minimum time from diagnosis to randomization of 3 months. Thus, since patients entered TEACH between 3 and 179 months after diagnosis (median: 32 months), the DFS times from primary diagnosis of BC are left-truncated for all patients; that is, patients included in this analysis had to have survived and remained disease-free long enough to enter TEACH. In addition, DFS times are right-censored for the 1,045 patients who remained disease-free at the conclusion of follow-up. To account for the left-truncation, patients were included in the risk set for a DFS failure at the time they entered the trial (measured from time of diagnosis) [4]. After the time patients had an event or were censored, they were excluded from the risk set calculations. Thus we obtained unbiased estimates of recurrence risk starting at 3 months post diagnosis, with the precision of the estimates increasing (and then decreasing) with time since diagnosis as more patients enter the trial (and then leave the trial).

The Kaplan-Meier method was used to estimate the empirical distribution of DFS from diagnosis, overall and by HR subgroups. The annual hazard function, giving the risk of disease at a specified time conditional on remaining disease-free up until that point in time, was estimated overall and by HR status using splines with separate splines fit for the cohort overall and by HR status [5]. The number of knots used for the splines was determined by comparing the Akaike information criterion (AIC) model fit statistics for a range of choices for number of knots and choosing the value that minimized the AIC statistic, which penalizes models with more knots to avoid overfitting [6]. Cox proportional hazards models were used to evaluate risk of DFS events by prognostic factors. These models rely on the assumption that the effect of a prognostic factor on risk of recurrence remains constant over time. As previous data have consistently shown time-varying hazards of recurrence by ER status in HER2-untested cohorts [7-9] potential violations of this assumption for each prognostic factor were evaluated by examining smoothed plots of the scaled Schoenfeld residuals over time and testing for an association between the residuals and time via regression [10].

## Results

Overall, 1,260 patients assigned to placebo and with centrally confirmed HER2+ disease were included in the analysis. A total of 55.5% (*N* = 699) had hormone receptor positive (HR+, estrogen (ER) and/or progesterone receptor (PR)-positive) disease, and 44.5% (*N* = 561) HR-negative disease (HR-, ER and PR-negative disease). Clinical characteristics are shown in Table 1. Median time from diagnosis to randomization was 32 (3 to 179) months and median follow-up from randomization was 42 (0 to 60) months. The resulting median time from diagnosis to last follow-up was 70 (9 to 216) months.

**Table 1:** **Baseline clinical characteristics of placebo patients with centrally confirmed HER2+ breast cancer in TEACH**

	***N*** **(%)**		
	**All**	**HR−**	**HR+**
	**(** ***N*** **= 1,260)**	**(** ***N*** **= 561)**	**(** ***N*** **= 699)**
**Median age, yrs (range)**	50 (22–81)	51 (24–81)	48 (22–80)
**Age range (%)**			
<40	217 (17)	78 (14)	139 (20)
40-50	446 (36)	160 (28)	286 (41)
50-60	414 (33)	224 (40)	190 (27)
60-70	155 (12)	83 (15)	72 (10)
>70	28 (2)	16 (3)	12 (2)
**Postmenopausal, n (%)**	854 (68)	403 (72)	451 (65)
**Race, n (%)**			
White	840 (67)	370 (66)	470 (67)
Asian	298 (23)	135 (24)	163 (23)
Black	35 (3)	16 (3)	19 (3)
Other/unknown	87 (7)	40 (7)	47 (7)
**Median time since initial diagnosis, yrs (range)**	3 (0–15)	2 (0–15)	3 (0–12)
**Years since initial diagnosis, n (%)**			
≤4	916 (73)	430 (77)	486 (70)
>4	344 (27)	131 (23)	213 (30)
0-1	278 (22)	138 (25)	140 (20)
**Hormone receptor status, n (%)**			
HR+ (ER+ or PR+)	695 (55)	-	695 (100)
ER+ and PR+	438 (35)	-	438 (63)
ER+ and PR-	170 (13)	-	170 (24)
ER- and PR+	87 (7)	-	87 (13)
HR- (ER- and PR-)	561 (45)	561 (100)	-
**Nodal involvement, n (%)**	698 (57)	303 (55)	395 (58)
**Low stage (T1 N0), n (%)**	220 (18)	92 (17)	128 (19)
**Prior or concurrent endocrine therapy (HR+ only), n (%)**			
Yes	625 (50)	-	625 (89)
No	74 (6)	-	74 (11)
N/A	561 (45)	561 (100)	-
**Adjuvant chemotherapy, n (%)**			
Anthracycline without taxane	710 (56)	303 (54)	407 (58)
Anthracycline with taxane	503 (40)	234 (42)	269 (39)
No anthracycline	47 (4)	24 (4)	23 (3)

HER2+, human epidermal growth factor receptor 2 positive; TEACH, Tykerb evaluation after chemotherapy; HR+, hormone receptor positive; ER+, estrogen receptor positive; PR+, progesterone receptor positive; ER-, estrogen receptor negative; HR-, hormone receptor negative; PR-, progesterone receptor negative.

**Figure 1:**
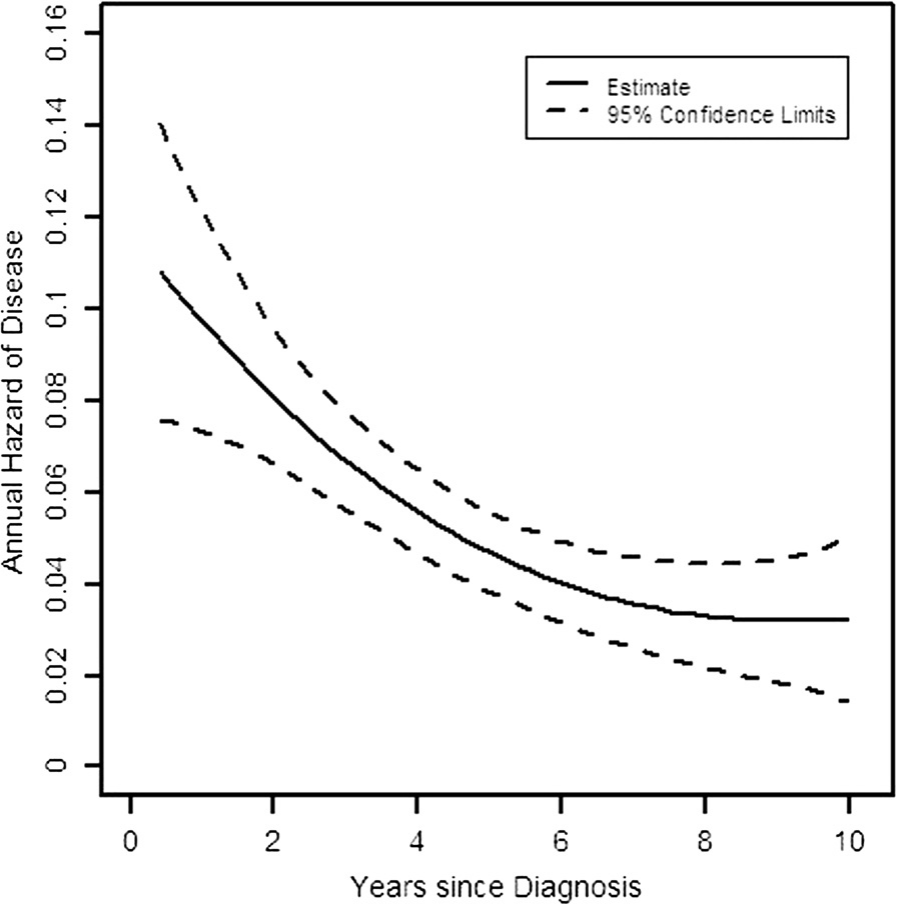
Annual hazard of disease recurrence in centrally confirmed HER2+ placebo patients in TEACH. HER2+, human epidermal growth factor receptor 2 positive; TEACH, Tykerb evaluation after chemotherapy.

In all centrally confirmed HER2+ placebo patients 5-year DFS was 75% and 10-year DFS was 61%. Figure 1 shows the hazard of recurrence for centrally confirmed HER2+ placebo patients. We were able to calculate the curve starting at 3 months post diagnosis, as this was the shortest time between diagnosis and trial entry. As more patients entered the trial, the confidence interval narrows, and widens again as the patient population at risk declines due to disease-free events and censoring. The annual hazard of recurrence for HER2+ patients not treated with anti-HER2 therapy was initially approximately 10% per year, dropping rapidly in years 1 to 5 and stabilizing at an ongoing risk of recurrence of about 3.5% per year at 6 to 10 years post diagnosis.

Analysis of DFS according to HR status showed a superior outcome for patients with HR+ (ER+ and/or PR+) disease, with a 4-year DFS of 84% versus 71% in the HR- (ER-/PR-) cohort (hazard ratio 0.72, *P* = 0.02).

**Figure 2:**
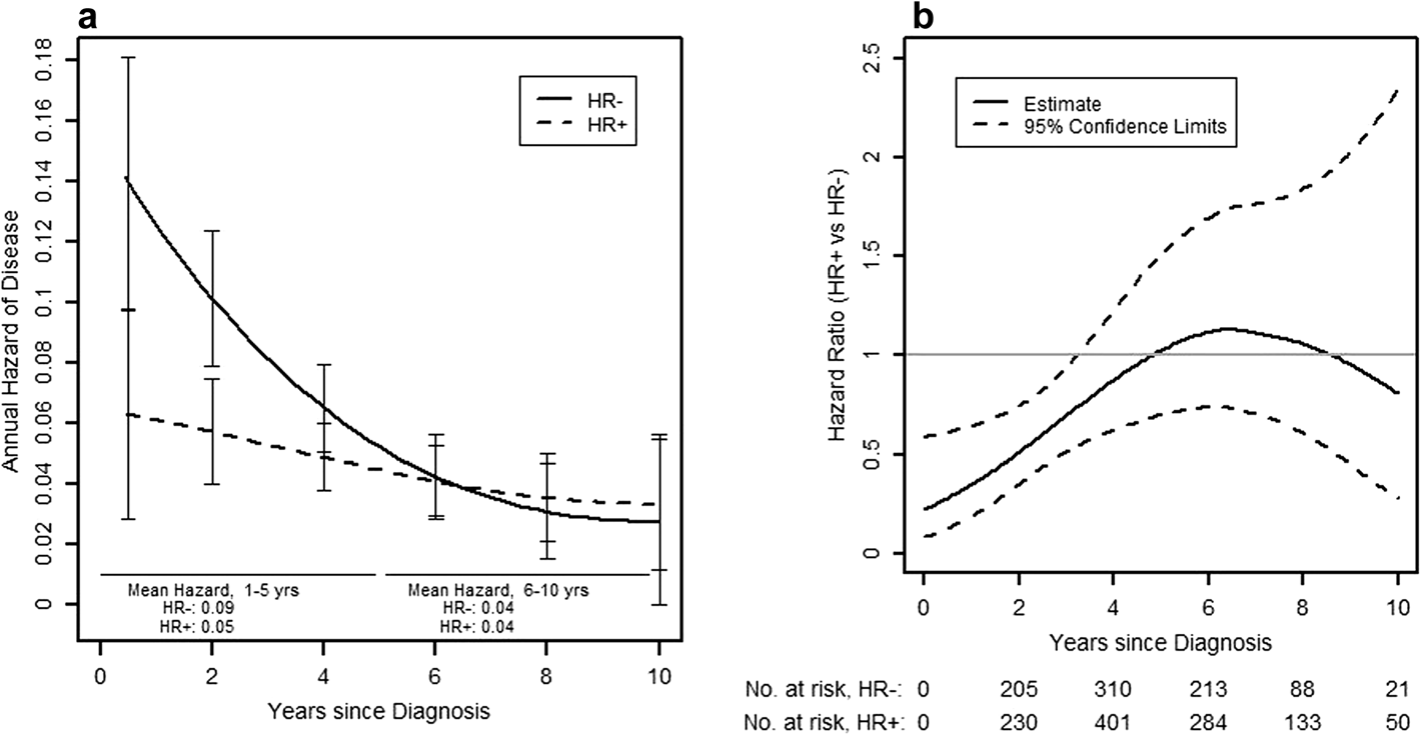
Annual hazards and time-dependent hazard ratio for disease recurrence in centrally confirmed HER2+ patients by HR status. **(a)** Annual hazard of disease recurrence in centrally confirmed HER2+ placebo patients in TEACH, according to HR status (95% confidence limits at 2-year intervals). (HR+ = ER+ and/or PR+; HR- = ER- and PR-). **(b)** Hazard ratio for disease recurrence in centrally confirmed HER2+ placebo patients in TEACH, comparing HR+ versus HR-. (HR+ = ER+ and/or PR+; HR- = ER- and PR-). ER-, estrogen receptor negative; ER+, estrogen receptor positive; HER2+, human epidermal growth factor receptor 2 positive; HR-, hormone receptor negative; HR+, hormone receptor positive; PR-, progesterone receptor negative; PR+, progesterone receptor positive; TEACH, Tykerb evaluation after chemotherapy.

The annual hazards of disease recurrence of centrally confirmed HER2+ patients by HR status are presented in Figure 2a, showing that the high hazard of recurrence in the overall cohort is almost exclusively attributed to patients with HR- disease. In addition, it can be seen that while HR+ patients have a relatively constant annual hazard of recurrence over time, HR- patients experience high annual hazards of recurrence early on, with a steep decrease over the first 5 to 6 years after diagnosis. The mean annual hazards of recurrence in years 1 to 5 from diagnosis were 9% versus 5% in HR- versus HR+ patients, respectively. In years 6 to 10, the annual hazards of recurrence were very similar between HR- and HR+ patients (mean hazard 4% in both groups).

The time-dependent hazard ratio for patients with HR+ versus HR- disease is given in Figure 2b. As suggested by the nonparallel hazard curves for HR+ versus HR- disease in Figure 2a, the test of the proportional hazards assumption was rejected indicating a nonconstant hazard ratio (*P* = 0.05). The analysis confirms a time dependence of the hazard ratio by HR status in HER2+ patients, indicating that the fixed hazard ratio of 0.72 over 10 years underestimates the difference between the HR+ and HR- groups in the first years after diagnosis. The hazard ratios from a Cox model stratified by time are 0.64 for years 1 to 5 and 0.93 for years 6 to 10, showing that the risk of early relapse is substantially higher for HER2+ HR- than for HER2+ HR+ patients.

**Figure 3:**
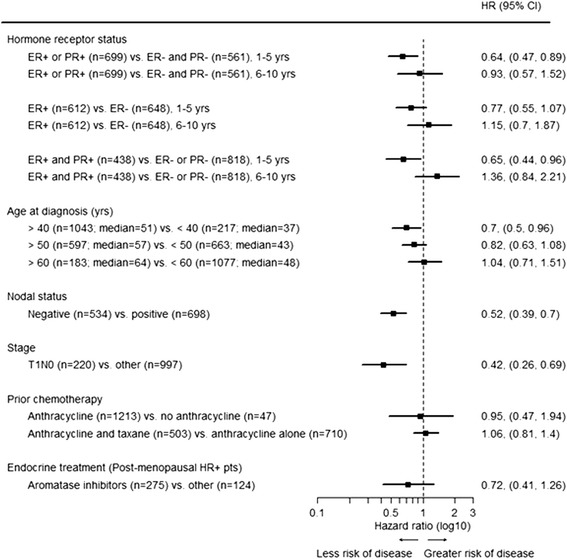
Univariable hazard ratios for risk of disease recurrence by prognostic factors in centrally confirmed HER2+ placebo patients in TEACH. HER2+, human epidermal growth factor receptor 2 positive; TEACH, Tykerb evaluation after chemotherapy.

Figure 3 depicts univariable analysis of hazard ratios for risk of recurrence, taking into account prognostic factors. Since tests of the proportional hazards assumption indicated nonconstant hazards for ER+ status in addition to HR+ status, hazard ratios are reported separately for years 1 to 5 and 6 to 10 post diagnosis. For the other prognostic factors in Table 2, the proportional hazards assumption was not violated. Patients <40 years at diagnosis had a worse prognosis, but this effect was only marginally statistically significant and not seen when different cutoffs for age (50, 60) were used. As expected, low tumor stage and negative nodes were significant prognosticators, each reducing recurrence risk by approximately 50% (hazard ratio 0.42 (0.26 to 0.69) and 0.52 (0.39 to 0.7), respectively). The additional use of taxane-based chemotherapy did not improve the prognosis of patients who were treated with anthracyclines (hazard ratio 1.06 (0.81 to 1.40)), and no difference was seen based on whether patients were treated with or without anthracyclines (hazard ratio 0.95 (0.47 to 1.94)). However, only few patients were treated without anthracyclines (*N* = 47).

**Table 2:** **Multivariable hazard ratios for risk of disease recurrence by prognostic factors in centrally confirmed HER2+ placebo patients in TEACH**

**Prognostic factor**	**Hazard ratio (95% CI)**	***P*** **value**
Hormone receptor (HR^2^) status	0.59 (0.42, 0.81)	0.002
HR+ vs. HR-, 1–5 yrs after diagnosis		
HR+ vs. HR-, 6–10 yrs after diagnosis	0.97 (0.59, 1.62)	0.92
Age at diagnosis (years)		
≥40 vs. <40	0.69 (0.49, 0.96)	0.03
Nodal status		
Negative vs. positive	0.62 (0.45, 0.87)	0.006
Stage		
T1 N0 vs. other	0.58 (0.33, 1.0)	0.05
Prior chemotherapy^1^		
Anthracycline versus no anthracycline	0.78 (0.38, 1.61)	0.51
Anthracycline + taxane versus anthracycline alone	0.85 (0.64, 1.14)	0.27

^1^Separate models, each including all other prognostic factors, were used to estimate the hazard ratio for anthracycline versus no anthracycline and anthracycline with taxane versus anthracycline alone; ^2^HR+ = ER+ or PR+; HR- = ER- and PR-. The hazard ratio estimate for each prognostic factor is adjusted for all other factors listed in the table. HER2+, human epidermal growth factor receptor 2 positive; TEACH, Tykerb evaluation after chemotherapy; CI, confidence interval.

Multivariable analysis of prognostic factors confirmed the significant influence of HR status and nodal status on risk of recurrence, whereas age at diagnosis as well as low stage were only marginally statistically significant (Table 2).

## Discussion

Our results confirm that HER2+ BC patients untreated with anti-HER2 therapy have a high risk of recurrence, particularly during years 1 to 5 post diagnosis. The annual hazard for recurrence in patients with tumors at least 1 cm in size starts at about 10% per year, decreases over time, and stabilizes at about 4% per year after 6 to 8 years. After 10 years approximately 40% of patients will have experienced a recurrence.

From previous data in HER2-untested cohorts [7-9,11,12], reporting that HR expression exhibits time-dependent nonproportional hazard effects on prognosis [8,9], it was unclear whether these effects would also be present in HER2+ patients, which have likely been a minority within the previously studied cohorts. Our results are novel, as we show that a difference in the hazard of recurrence between HR+ and HR- patients is evident in HER2+ patients treated with modern chemotherapy and endocrine therapy but not with anti-HER2 therapy. HER2+ HR- patients have an average yearly rate of recurrence of 9% in years 1 to 5 compared to an average of 5% for HER2+ HR+ patients. HR expression is thus an important determinant of long-term outcome in HER2+ patients not treated with anti-HER2 therapy.

Apart from a difference in the mean hazard of recurrence, we also show that the shapes of the hazard curves differ significantly between HER2+ HR+ and HER2- HR- patients. By showing that there are indeed fundamental differences in the behavior of HR- and HR+ HER2+ BC (with the time-dependent hazard ratio crossing 1) and providing the hazard functions for each group over time, our data will allow correct up-to-date estimates of absolute treatment effects for clinical trials of future novel therapies in HER2+ patients.

In an indirect way, our data also allow inferences about the effect of endocrine therapy in HER2+ BC patients not treated with anti-HER2 therapy: it would have been possible that the flattening effect of endocrine therapy on the early hazard of recurrence of HR+ patients that was seen in HER2-unknown (that is mostly HER2-) cohorts [8,9,11] is not seen if the HER2 epitope is not targeted as well, thus resulting in a similar early peak in HER2+ HR+ patients as seen in HER2+ HR- patients. It is therefore important to note that the reduction in the early hazard of recurrence achieved by endocrine therapy in ER+ patients (with unknown HER2 status) seems to still be evident in HER2+ HR+ patients, underlining the importance of effective endocrine treatment, including prolonged therapy, in these patients even when anti-HER2 therapy administration is not possible.

Subgroup analyses showed that the risk of recurrence was not significantly influenced by menopausal status or age. However, lymph node involvement and higher tumor stage were each associated with significant increases in the risk for disease recurrence, underlining the importance of tumor stage for the prognosis of HER2+ BC patients.

Based on our data, HR expression can thus be used - in addition to tumor stage - in HER2+ patients to identify those at the highest risk. As the relative effect of trastuzumab is similar in patients with HR+ and HR- disease [13-15] the absolute benefit of treatment with trastuzumab is based on a patient’s absolute baseline risk. Therefore, by reporting that trastuzumab-naïve HER2+ HR- patients have a substantially higher recurrence rate than HER2+ HR+ patients, we infer and show that HER2+ HR- will derive higher absolute treatment benefits from trastuzumab treatment. Many countries worldwide with limited resources currently opt not to provide adjuvant trastuzumab at all because of economic reasons. Irrespective of the ultimate goal to provide adjuvant anti-HER2 therapy to all HER2+ patients as defined in the adjuvant trials, our data might therefore provide a strong rationale for offering anti-HER2 therapy to HER2+ HR- subpopulations.

HER2 positivity has been suggested to be predictive for response to certain chemotherapies, including anthracyclines and taxanes [16,17]. Comparison of the risk of recurrence by adjuvant chemotherapy (univariable and multivariable analyses) in our cohort did not show a decrease in risk by adding taxanes in the absence of anti-HER2 therapy. However, these results might be biased by underlying differences in risk, as we were not able to analyze reasons for including or excluding taxanes in the treatment of our patients.

Limitations of our analyses include our inability to collect (competing) causes of death so that we are not able to provide overall survival data for our cohort. As survival post progression is expected to be very long in this cohort of patients, the TEACH trial focused on PFS as a primary endpoint as do all adjuvant BC trials. Overall survival data are not uniformly available within these trials in patients experiencing a recurrence. As cost-effectiveness analyses are typically based on ‘quality-adjusted life years gained’, information on overall survival, ideally by age and comorbidities, would have been the preferable outcome measure as a basis for treatment decisions in health care settings with limited resources. In addition, not all HER2+ BC patients are represented in this analysis, as, similar to the large adjuvant trastuzumab trials [13,18,19], patients with small, low-risk tumors were not included in TEACH. However, even if absolute risks and benefits are smaller in low-risk tumors, we assume that our main findings, including the difference in the behavior of recurrence between HER2+ HR+ and HER2+ HR- tumors, hold true in biologically similar, lower risk situations.

Overall our results show an ongoing risk of recurrence in HER2+ BC patients, not treated with anti-HER2 therapy over 10 years. The reported time-varying hazard functions by HR status will allow up-to-date estimates of absolute treatment effects in these patients and might serve as ‘historical’ control for further analyses of other novel interventions being considered. Based on the adjuvant data of trastuzumab in HER2+ early BC, health care providers should strive to ultimately provide trastuzumab to all eligible patients. However, in health care settings where approval of expensive drugs along the lines of first-world scenarios is not affordable, knowledge about the high risk of recurrence in HER2+ HR- patients might help to devise subgroup-specific surveillance or anti-HER2 treatment strategies in this circumstance.

## Conclusions

Outcomes in patients with HER2+ early BC not receiving anti-HER2 therapy strongly depend on HR expression. The mean hazard of recurrence in years 1 to 5 post diagnosis in HER2+ HR- compared to HER2+ HR+ patients is 9 vs. 5%/year (hazard ratio 0.59, *P* = 0.002). This finding is particularly relevant in health care settings with limited access to adjuvant anti-HER2 treatment.

## Additional file

Additional file 1:
**List of ethics review boards that approved the TEACH trial in participating centers.**

## Abbreviations

AIC: Akaike information criterion
BC: breast cancer
DFS: disease-free survival
ER+: estrogen receptor positive
ER-: estrogen receptor negative
HER2+: human epidermal growth factor receptor 2 positive
HR+: hormone receptor positive
HR-: hormone receptor negative
PR+: progesterone receptor positive
PR-: progesterone receptor negative
TEACH: Tykerb evaluation after chemotherapy
