# Supplementary material for: Long-term hazard of recurrence in HER2+ breast cancer patients untreated with anti-HER2 therapy
Source: Breast Cancer Res. 2015 Apr 16;17(1):56. doi: 10.1186/s13058-015-0568-1 (PMC4423419; doi:10.1186/s13058-015-0568-1)
Supplement: Additional file 1: — List of ethics review boards that approved the TEACH trial in participating centers. [file 13058_2015_568_MOESM1_ESM.pdf]

LIST OF INVESTIGATORS AND IECS/IRBS FOR EGF105485

Investigator	Investigator no./Center no.	Description of Research Facility, Hospital/ Institution, and Address	Name of IEC/IRB Committee, Address, Committee Chair
**Argentina**			
Alvarez, Anna. MD*	100077/032611	Instituto Angel H. Roffo, v San Martin 5481, (1417)-Ciudad de Buenos Aires, Argentina.	Comite de Etica, "Dr. Virgilio G. Foglia", Tucuman 335 - 7 "D", (1049) - Ciudad de Buenos Aires, Argentina.<br><br>Chairperson: Dr. Carlos Barclay<br><br>Comite de Docencia e Investigacion, del Instituto Angel H. Roffo, (1417) Ciudad de Buenos Aires, Argentina.<br><br>Chairperson: Dr. Carlos Barclay<br><br>Comite de Etica, del Instituto Angel H. Roffo, (1417) Ciudad de Buenos Aires, Argentina.<br><br>Chairperson: Dr. Carlos Barclay

Investigator	Investigator no./Center no.	Description of Research Facility, Hospital/ Institution, and Address	Name of IEC/IRB Committee, Address, Committee Chair
Blajman, Cesar. MD	105546/052839	ISIS Centro Especializado de LUCE SA, Urquiza 3077 (S3000FFU) - Santa Fe, Argentina.	Comite de Etica Independiente en Investigacion Clinica "Dr. Carlos A. Barclay", Larrea 1381 3°A. C1117ABK, Buenos Aires, Argentina.<br>Chairperson: Carlos Barclay<br><br>Comite de Docencia e Investigacion de ISIS Centro Especializado Urquiza 3077, (S3000FFU) - Santa Fe. Argentina.<br>Chairperson: Samuel Seiref
Fein, Luis. MD	073914/032503	Centro Oncologico de Rosario, Av, Boulevard Ororio 1085, (2000) - Rosario Santa Fe-Argentina.	Comite de Etica, "Dr. Virgilio G. Foglia", Tucuman 335 - 7 "D", (1049) - Ciudad de Buenos Aires, Argentina.<br>Chairperson: Carlos Barclay<br><br>Comite de Docericia e Investigacion del centro Oncologico de Rosario, Av. Boulevard Orono 1085 (2000) Rosario Santa Fe

Investigator	Investigator no./Center no.	Description of Research Facility, Hospital/ Institution, and Address	Name of IEC/IRB Committee, Address, Committee Chair
			Argentina.<br>Chairperson: Dr. Daniel Sarcuno
Jankilevich, Gustavo. MD	095058/046416	Instituto Medico Especializado, Hidalgo 568, (C1405BCH) Ciudad Autonoma de Buenos Aires<br>Argentina.	Comite de Etica, "Dr. Virgilio G. Foglia", Tucuman 335 - 7 "D", (1049) - Ciudad de Buenos Aires, Argentina.<br>Chairperson: Dr. Jadzinsky Mauricio<br><br>Comite de Revision Institucional delInstituto Medico Especializado", Hidalgo 568, (C1405BCH) Ciudad Autonoma de Buenos Aires Argentina.<br>Chairperson: María Victoria Bertolino
Lacava, Juan. MD	112531/037347	Unidad Oncologica de Neuquen<br>Rivadavia 360, (8300)- Neuquen<br>Argentina.	Comite de Etica, "Dr. Virgilio G. Foglia", Tucuman 335 - 7 "D", (1049) - Ciudad de Buenos Aires, Argentina.<br>Chairperson: Carlos Barclay

Investigator	Investigator no./Center no.	Description of Research Facility, Hospital/ Institution, and Address	Name of IEC/IRB Committee, Address, Committee Chair
			Comite Independiente de Docencia e Investigacion CIDel", Rivadavia 360, (8300) Neuquen Argentina.<br><br>Chairperson: Dr. Ernesto F. Ruiz
Lerzo, Guillermo. MD	038842/049502	IMCABA, Estado de Israel 4718, (C 1185AAS) Ciudad Autonoma de Buenos Aires, Argentina.	Comite de Etica Independiente en Investigacion Clinica "Dr. Carlos A. Barclay", Larrea 1381 3°A. C1117ABK, Ciudad Autónoma de Buenos Aires, Argentina.<br><br>Chairperson: Carlos Barclay
Lerzo, Guillermo. MD	038842/034125	LUCEN, Jauretche 24, (1405) - Ciudad Autonoma de Buenos Aires, Argentina.	Comite de Etica, "Dr. Virgilio G. Foglia", Tucuman 335 - 7 "D", (1049) - Ciudad de Buenos Aires, Argentina.<br><br>Chairperson: Dr. Rome Nicolas

Investigator	Investigator no./Center no.	Description of Research Facility, Hospital/ Institution, and Address	Name of IEC/IRB Committee, Address, Committee Chair
Richardet, Eduardo. MD	112486/037349	IONC, Dean Funes 477, (X5000JFK) – Cordoba, Argentina.	Comite Institucional de Etica de la Investigacion en Salud - CIEIS IONC, Balcarce 451, (X5000JFK)- Ciudad de Córdoba, Provincia de Córdoba, Argentina.<br><br>Chairperson: Pablo Carmignani
Varela, Mirta. MD	094383/032610	CER Instituto Medico, Vicente Lopez 1441, (1878)- Quilmes,Buenos Aires- Argentina	Comité de Ética de CER Instituto Médico (CECIC) Avda. Vicente López 1441 (1878) Quilmes O., Provincia de Buenos Aires, Argentina.<br><br>Chairperson: Dr. Damián Del Percio
Zarba, Juan. MD	093585/032502	Centro Medico San Roque, Balcarce 579, (4000) - San Miguel de Tucuman, Tacuman Argentina.	Comite de Etica Independiente en Investigacion Clinica "Dr. Carlos A. Barclay", Larrea 1381 3°A. C1117ABK, Ciudad Autónoma de Buenos Aires, Argentina.<br><br>Chairperson: Carlos Barclay

Investigator	Investigator no./Center no.	Description of Research Facility, Hospital/ Institution, and Address	Name of IEC/IRB Committee, Address, Committee Chair
			Comite de Docencia e Investigacion Centro Medico San Roque, Balcarce 579, (4000)-San Miguel de Tucuman, Provincia de Tucumán, Argentina.<br><br>Chairperson: Luis F. Medina
**Australia**			
Beadle, Geoffrey. MBBS<br>McCarthy, Nicole. MBBS (Former PI)	040168/032839	Royal Brisbane and Women's Hospital, Butterfield Street, Herston, Queensland, 4029 Australia.	Royal Brisbane and Women's Hospital Human Research Ethics Committee Level 7, Block 7, Royal Brisbane and Women's Hospital, Butterfield Street, Herston, Queensland, 4029 Australia.<br><br>Chairperson: Dr. Conor J Brophy

Investigator	Investigator no./Center no.	Description of Research Facility, Hospital/ Institution, and Address	Name of IEC/IRB Committee, Address, Committee Chair
Boyle, Frances. PhD	060713/031103	The Mater Hospital, St Vincent's and Mater Health, Sydney, Ltd, 25 Rocklands Road North Sydney, New South Wales, 2060, Australia.	St Vincent's Hospital Human Research Ethics Committee, Research Office, Level 6 deLacy Building, 390 Victoria Street, Darlinghurst NSW 2010, Australia.<br><br>Chairperson: Prof. Jo-Anne Brien
Chan, Arlene. MBBS	060716/031048	Mount Medical Centre, 146 Mounts Bay Road, Perth 6000, Western Australia, Australia.	Mount Hospital Ethics Committee, 150 Mounts Bay Road, Perth 6000, Western Australia, Australia.<br><br>Chairperson: Jade Phelan.
Chern, Boris. MBBS	027038/031105	Redcliffe Hospital, Anzac Avenue, Redcliffe-Queensland 4020, Australia.	The Redcliffe-Caboolture Ethics Committee, Redcliffe Hospital, Anzac Avenue, Redcliffe-Queensland 4020, Australia.<br><br>Chairperson: Mr Mark Zgrajewski

Investigator	Investigator no./Center no.	Description of Research Facility, Hospital/ Institution, and Address	Name of IEC/IRB Committee, Address, Committee Chair
Chirgwin, Jacqueline. MBBS	076771/031049	Maroondah Breast Clinic, 20 Grey St, Ringwood East, Victoria 3135, Australia.	Eastern Health Research and Ethics Committee, Level 2, Clive Ward Centre, 16 Arnold Street, Box Hill, Victoria 3128, Australia.<br><br>Chairperson: Prof Bridie Kent
Chirgwin, Jacqueline. MBBS	076771/031050	Box Hill Hospital, Nelson Road, Box Hill, Victoria 3128, Australia.	Eastern Health Research and Ethics Committee, Level 2, Clive Ward Centre, 16 Arnold Street, Box Hill, Victoria 3128,.<br><br>Chairperson: Prof Bridie Kent
Dalley, David. MBBS	096713/031109	St Vincents Hospital, 390 Victoria Street, Darlinghurst, NSW 2010, Australia.	St Vincent's Hospital Human Research Ethics Committee, Research Office, Level 6 deLacy Building, 390 Victoria Street, Darlinghurst NSW 2010, Australia.<br><br>Chairperson: Prof. Jo-Anne Brien

Investigator	Investigator no./Center no.	Description of Research Facility, Hospital/ Institution, and Address	Name of IEC/IRB Committee, Address, Committee Chair
deBoer, Richard. MBBS	030359/032570	Royal Melbourne Hospital, Department of Medical Oncology, Grattan Street, Parkvil le, Victoria, 3050, Australia.	Melbourne Health Human Research Ethics Committee, Royal Melbourne Hospital, Grattan Street, Parkville, Victoria, 3050, Australia.<br><br>Chairperson: Prof. Peter Colman
deBoer, Richard. MBBS	030359/032571	Western Hospital, Department of Medical Oncology, Gordon Street, Footscray, Victoria, 3011 Australia.	Melbourne Health Human Research Ethics Committee, Royal Melbourne Hospital, Grattan Street, Parkville, Victoria, 3050 Australia.<br><br>Chairperson: Prof. Peter Colman
Foo, Serene. MBBS	092805/031111	Breast Unit at Mercy Private, Level 2, 166 Gipps Street, East Melbourne, Victoria, 3002 Australia.	Mercy Health and Aged Care Inc, 678 Victoria Parade, Richmond, Victoria, 3121, Australia.<br><br>Chairperson: Professor Janis (John) Ozolins.<br>Austin Health Research and Ethics

Investigator	Investigator no./Center no.	Description of Research Facility, Hospital/ Institution, and Address	Name of IEC/IRB Committee, Address, Committee Chair
			Committee, Austin Hospital, 145 Studley Road, Heidelberg, Victoria, 3084 Australia.<br><br>Chairperson: Prof David Taylor
Foo, Serene. MBBS	092805/034704	Austin Hospital Campus, Austin Health, 145 Studley Road, Heidelberg, Victoria, 3084 Australia.	Mercy Health and Aged Care Inc., 678 Victoria Parade, Richmond, Victoria, 3121 Austrailia.<br><br>Chairperson: Professor Janis (John) Ozolins.<br><br>(Austin) Austin Health Research and Ethics Committee, Austin Hospital, 145 Studley Road, Heidelberg, Victoria, 3084 Australia.<br><br>Chairperson: Prof David Taylor

Investigator	Investigator no./Center no.	Description of Research Facility, Hospital/ Institution, and Address	Name of IEC/IRB Committee, Address, Committee Chair
Koczwara, Bogda. MBBS	067281/031106	Flinders Medical Centre, Department of Medical Oncology,<br>Flinders Drive, Bedford Park, South Australia, 5042 Australia.	Flinders Clinical Research Ethics Committee, Level 2, Flinders Medical Centre, Flinders Drive, Bedford Park, South Australia, 5042 Australia.<br><br>Chairperson: Prof David Gordon
Lynch, Jodi. MBBS	073424/031104	St George Hospital, Gray Street, Kogarah, NSW 2217 Australia.	South Eastern Sydney Local Health Network HREC (Southern Sector) c/o The St George Hospital, Gray Street, Kogarah, NSW 2217 Australia.<br><br>Chairperson: Associate Professor Winston Liauw
Nowak, Anna. MBBS	089087/031110	Sir Charles Gairdner Hospital, Hospital Avenue, Nedlands WA 6009, Australia.	Sir Charles Gairdner Hospital, Human Research Ethics Committee, Sir Charles Gairdner Hospital, 1st Floor E Block, Hospital Avenue, Nedlands WA 6009, Australia.<br>Chairperson: Prof. D.A. Joyce

Investigator	Investigator no./Center no.	Description of Research Facility, Hospital/ Institution, and Address	Name of IEC/IRB Committee, Address, Committee Chair
Patterson, William. MBBS	105310/034673	Lyell McEwin Hospital, Department of Haematology/Oncology, Oldham Road, Elizabeth Vale, South Australia, Australia 5112	Central Northern Adelaide Health Service Ethics of Human Research Committee, The Queen Elizabelh Hospital, 28 Woodville Road, Woodville South, 5011 Australia.<br><br>Chairperson: Timothy Matthew
Patterson, William. MBBS	105310/034674	The Queen Elizabeth Hospital, Department of Haematology and Oncology, 28 Woodville Road, Woodville South, South Australia 5011 Australia.	Central Northern Adelaide Health Service Ethics of Human Research Committee, The Queen Elizabelh Hospital, 28 Woodville Road, Woodville South, South Australia 5011 Australia.<br><br>Chairperson: Timothy Matthew

Investigator	Investigator no./Center no.	Description of Research Facility, Hospital/ Institution, and Address	Name of IEC/IRB Committee, Address, Committee Chair
Shannon, Catherine. MBBS(Hons)	067276/031051	Mater Adult Hospital, Raymond Terrace, South Brisbane, Queensland, 4101 Australia.	Mater Health Services Human Research Ethics Committee, Room 235, Level 2 Aubigny Place, Raymond Terrace, South Brisbane, Queensland, 4101 Austratia.<br><br>Chairperson: Mr Andrew Crowden
Snyder, Raymond. MBBS	037288/031053	St Vincents Hospital, Healey Wing, 41 Victoria Parade, Fitzroy, Victoria 3065, Australia.	St Vincent's Hospital Human Research Ethics Committee, St Vincent's Hospital, 1st Floor Healy Wing, 41 Victoria Parade,<br>Fitzroy, Victoria, 3065 Australia.<br><br>Chairperson: Prof Jo-anne Brien
Underhill, Craig. MBBS<br>Clarke, Kerrie. MD (Former PI)	055873/031052	Border Medical Oncology, Murray Valley Private Hospital, Nordsvan Drive, Wodonga, Victoria 3690, Australia.	Wodonga Regional Health Service Joint Hospitals' Ethics Committee, Vermont Street (PO Box 156), Wodonga, Victoria 3689 Australia.<br><br>Chairperson: Greg Pearl

Investigator	Investigator no./Center no.	Description of Research Facility, Hospital/ Institution, and Address	Name of IEC/IRB Committee, Address, Committee Chair
**Belgium**			
Awada, Ahmad. MD	027308/030859	Institut Jules Bordet, Centre des Tumeurs de l'Universite Libre de Bruxelles, Boulevard de Waterloo 121, B-1000 Brussels, Belgium.	Comite D'Ethique, Institut Jules Bordet, Boulevard de Waterloo 125, B-1000 Bruxelles, Belgium.<br><br>Chairperson- Dr Thierry Gil
**Brazil**			
Barrios, Carlos Henrique. MD	085726/038824	Centro de Pesquisas Clínicas em Oncologia, Av. Ipiranga 6690 sala 228, Porto Alegre, Rio Grande Do Sul, 90610 000 Brazil.	Comite de Etica em Pesquisa do Hospital Sao Lucas da PUC/RS AV. Ipiranga 6690 3 andar Jardim Botanico, Zip Code: 90. 610-000, Porto Alegre- RS.<br>Local IRB Chairperson: Rodolfo Herberto Schneider<br><br>CONEP, Esplanada dos Ministerios-Bloco G,anexoa Ala B, sala 147, 1 andar, Brasilia, Brazil.<br>National IRB Chairperson: Gysélle Saddi

Investigator	Investigator no./Center no.	Description of Research Facility, Hospital/ Institution, and Address	Name of IEC/IRB Committee, Address, Committee Chair
			Tannous. CONEP - Comissao Nacional de Etica em Pesquisa
Cortes, Eduardo. MD	079294/038815	Hospital Universitario Clementino Fraga Filho-UFRJ, Ilha do Fundão, Rio de Janeiro, 21941-913, Brazil.	Comite de Etica em Pesquisa Clinica da UFRJ, Hospital UniverSitario Clementino Fraga Filho - Faculdade de Medicina<br>Rua Professor Rodolpho Paulo Rocco. 255. Ilha do Fundao, Rio de Janeiro - RJ zip code: 21941-913 – Brazil.<br>Local IRB Chairperson: Alice Helena Duarte Violante<br><br>CONEP. Esplanada dos Ministerios-Bloco G. anexo a Ala B. sala 147, 1oandar Brasilia - DF - ZIP CODE 70058-900 – Brazil<br>National IRB Chairperson: Gysélle Saddi Tannous. CONEP - Comissao Nacional de Etica em Pesquisa.

Investigator	Investigator no./Center no.	Description of Research Facility, Hospital/ Institution, and Address	Name of IEC/IRB Committee, Address, Committee Chair
Cruz, Felipe Jose	203252/038827	Faculdade de Medicina do ABC, Endereco: Avenida Principe de Gales, 821 - Anexo III, 09060-650 Santo Andre - SP – Brazil.	Comite de Eotica em Pesquisa Clinica da Faculdade de Medicina do ABC; Avenida Principe de Gales, 821 – Santo. Andre - SP – 09060-650 Brazil.<br>Local IRB Chairperson: Márcia R. G. Tamosauskas<br><br>CONEP, Esplanada dos Ministerios-Bloco G, anexo aAla B, sala 147,1oandar, Brasilia, Brazil- CEP 70058-900.<br><br>National IRB Chairperson: Gysélle Saddi Tannous.CONEP - Comissao Nacional de Etica em Pesquisa.
Oliveira, Célia	094615/038828	Instituto Brasileiro de Controle do Cancer, Avenida Alcantara Machado, 2576, CEP: 03102-002· SAo Paulo - SP – Brazil.	Comite de Eotica em Pesquisa Clinica da IBCC; Avenida Alcantra Machado, 2576 - CEP: 03102-002 - Sao Paulo - SP- Brazil.<br>Local IRB Chairperson: José Costa de

Investigator	Investigator no./Center no.	Description of Research Facility, Hospital/ Institution, and Address	Name of IEC/IRB Committee, Address, Committee Chair
			Andrade<br><br>CONEP, Esplanada dos Ministerios-Bloco G, anexo aAla B, sala 147,1 andar, Brasilia, CEP 70058-900 Brazil.<br><br>National IRB Chairperson: Gysélle Saddi Tannous CONEP - Comissao Nacional de Etica em Pesquisa.
Wainstein, Alberto. MD	085735/038830	Biocancer, Bernardo Monteiro 918, suíte 904, Belo Horizonte, 30150-281, Brazil.	Comite de etica em Pesquisa Clinica do Hospital Alberto Cavalcanti, Alameda Alvaro Celso, 100 - Santa Efigenia - Belo Horizonte - MG 30.150-260 Brazil.<br><br>Local IRB Chairperson: Vanderson Assis Romualdo<br><br>CONEP, Esplanada dos Ministerios-Bloco

Investigator	Investigator no./Center no.	Description of Research Facility, Hospital/ Institution, and Address	Name of IEC/IRB Committee, Address, Committee Chair
			G,anexo a Ala B,sala 147,1oandar - Brasilia - DF 70.058-900 Brazil.<br>National IRB Chairperson: Gysélle Saddi Tannous CONEP - Comissao Nacional de Etica em Pesquisa.
Zereu, Manuela. MD	049576/038831	Irmandade da Santa Casa de Misericórdia de Porto Alegre, Rua Annes Dias, 295, Porto Alegre, 90020-090, Brazil.	Comite de Etica em Pesquisa Clinica da Irmandade Santa Casa de Misericordia de Porto Alegre<br>Rua Professor Annes Dias, 295<br>Porto Alegre - RS CEP: 90020-090 Brazil.<br><br>Local IRB Chairperson: Cláudio Teloken<br><br>CONEP, Esplanada dos Ministerios-Bloco G,anexoa Ala B, sala 147, 1oandar, Brasilia, Brazil-CEP 70098-900<br><br>National IRB Chairperson: Gysélle Saddi

Investigator	Investigator no./Center no.	Description of Research Facility, Hospital/ Institution, and Address	Name of IEC/IRB Committee, Address, Committee Chair
			Tannous CONEP - Comissao Nacional de Etica em Pesquisa.
**Canada**			
Blondal, John. MD	005245/038974	Saint. Joseph's Health Centre, 30 The Queensway. SSW 601, Toronto, ON M6R 1B5 Canada	Dept Clinical Ethics, Saint Joseph's Health Centre, 30 The Queensway, Toronto, Ontario, M6R 1B5 Canada.<br><br>Chairperson- Hazel Markwell
Chang, Jose. MD	005117/033427	Lakeridge Health Osbawa, RS Mclaughlin Durham Regional Cancer Centre, 1 Hospital Court Osbawa, ON L1G 2B9 Canada.	Research Ethics Board, Lakeridge Health Osbawa, 1 Hospital Court, Oshawa, ON L1G 2B9 Canada.<br><br>Chairperson- John Montgomery
Desjardins, Pierre. MD	004711/033950	Hopital Charles LeMoyne, 3120, Boulevard Tascbereau, Greenfield Park QC J4 V 2H1 Canada.	Comite d'ethique de la recherché Hopital Charles LeMoyne 3120, boulevard Taschereau, Greenfield Park, Quebec, J4V 2H1 Canada.<br><br>Chairperson- Sylvain Brunet

Investigator	Investigator no./Center no.	Description of Research Facility, Hospital/ Institution, and Address	Name of IEC/IRB Committee, Address, Committee Chair
Goel, Rakesh. MD	005200/029352	General Campus, 501 Smyth Road, Ottawa Ontario K1H 8L6 Canada.	Ottawa Hospital Research Ethics Board, Ottawa Hospital, Civic Campus, 751 Parkdale Avenue, Suite 106, Ottawa, ON K 1 Y 117 Canada.<br><br>Chairperson- Raphael Saginur
Haider, Kamal-Uddin MD	092020/030653	Saskatoon Cancer Centre, 20 Campus Drive, Saskatoon, SK S7N 4H4, Canada.	University of Saskatchewan BioMedical Research Ethics Board (Bio-REB) Research Ethics Office, University of Saskatchewan, NRC - Plant Biotechnology Research Institute, 1607 - 110 Gymnasium Place, Saskatoon, SK S7N 4J8 Canada.<br><br>Chairperson- Gord McKay
Haq, Rashida MD	087784/033423	Saint Michael's Hospital, Medical Day Care Clinic, 2nd floor, Queen wing, 30 Bond Street, Toronto, Ontario, M5B 1 W8 Canada.	Saint Michael's Hospital, Research Ethics Board, 30 Bond Street, Toronto, ON M5B 1 W8 Canada.<br><br>Chairperson- Bob Hyland.

Investigator	Investigator no./Center no.	Description of Research Facility, Hospital/ Institution, and Address	Name of IEC/IRB Committee, Address, Committee Chair
Krishnan, Mukta. MD	100095/032620	Royal Victoria Hospital, 201 Gcorgian Drive, Barrie, ON L4M 6M2, Canada.	The Reasearch Ethics Soard of the Royal Victoria Hospital, 201 Georgian Drive, Barrie. Ontario. L4M 6M2 Canada.<br><br>Chairperson- Laura Crook
Laing, Kara. MD	029623/034587	Dr, H. Bliss Murphy Cancer Centre, 300 Prince Philip Drive, St, John's, NL A1B 3V6 Canada.	Human Investigation Committee Eastern Trust Building, 95 Bonaventure Avenue, Saint John's, NL, A1B 2X5 Canada.<br><br>Chairperson- Ban Younghusband.
Madarnas, Yolanda. MD	028295/032618	Cancer Centre of Southeastern Ontario at Kingston General Hospital, 25 King Street West Kingston, Ontario, K7L 51'9 Canada	Queen's University Health Sciences and Affiliated Teaching Hospitals Research Ethics Board Office of Research Services Flemming Hall - Jemmett Wing, 99 University Ave Kingston, Ontario, K7L3N6 Canada.<br><br>Chairperson- Albert Clark.

Investigator	Investigator no./Center no.	Description of Research Facility, Hospital/ Institution, and Address	Name of IEC/IRB Committee, Address, Committee Chair
Miller, Wilson. MD	055728/029404	Sir Mortimer B. Davis - Jewish General Hospital, Clinical Research Unite, RM E: 872 3755 Cote St-Catherine, Montreal, QC H3T IE2 Canada.	S.M.B.D-Jewish General Hospital Research Ethics Committee, Sit Mortbner B. Davis - Jewish General Hospital, 3755 Chemin de la. Cote Ste Catherine, Rm: A-925, Montreal, QC H3T IE2 Canada.<br><br>Chairperson- Carolyn Ells.
Myers, Robert. MD	093553/029620	Credit Valley Hospital, Carlo Fidani Peel. Regional Cancer Centre, 2200 Bglinton Avenue West, Mississauga, Ontario, L5M 2NI Canada.	Credit Valley Hospital c/o Research Administration, Research Review Committee, 2200 Eglinton Avenue West Mississauga. ON L5M 2N1 Canada.<br><br>Chairperson- Connie Day.
Potvin, Kylea. MD	133136/046167	London Health Sciences CentreLondon Regional Cancer Program 790 Commissioner's Road E, Room A3-834 London, Ontario, N6A 4L6 Canada.	Ontario Cancer Research Ethics Board, Ontario Institute Cancer Research Network MaRS Centre South Tower, 101 College Street, Suite 800, Toronto, Ontario, M5G 0A3 Canada.<br><br>Chairperson- Jack Holland.

Investigator	Investigator no./Center no.	Description of Research Facility, Hospital/ Institution, and Address	Name of IEC/IRB Committee, Address, Committee Chair
Provencher, Louise. MD	007714/034451	Centre Hospitalier Affilie Universilaire de Quebec (CHA) Hopilal du Saint-Sacrement 1050 chemin Ste·Foy Quebec, DC, GIS 4L8 Canada	Comite d'ethique de la recherché Centte Hospitalier Affilie Universitaire de Quebec (CHA) Hospital du Saint-Sacrement, 1050 chemin Ste-Foy, Quebec. QC, G1S 4LB Canada.<br><br>Chairperson- Ana Marin.
Bin, James. MD<br>Rahim, Yasmin. MD (Former PI)	186256/033424	Toronto East General Hospital 825 Coxwell A venue, Toronto, Ontario, M4C 3E7 Canada.	Ontario Cancer Research Ethics Board, Ontario Institute Cancer Research Network, MaRS Centre South Tower, 101 College Street. Suite 800, Toronto. Ontario, M5G 0A3 Canada.<br><br>Chairperson- Jack Holland.
Rayson, Daniel. MD	098006/031683	QEII Health Science Centre, Nova Scotia Cancer Centre, 5820 University Avenue, Halifax, NS B3H 1V7 Canada.	Capital District Health Authority Research Ethics Board, QEII Centre for Clinical Research, 5790 University Avenue, Room 118, Halifax, NS B3H 1V7 Canada.<br><br>Chairperson- Richard Hall.

**Investigator**	**Investigator no./Center no.**	**Description of Research Facility, Hospital/ Institution, and Address**	**Name of IEC/IRB Committee, Address, Committee Chair**
Sehdev, Sandeep	026463/029621	William Osler Health Centre, Brampton Memorial Hospital, 20 Lynch Street, Brampton, Ontario, L6W 2Z8 Canada.	William Osler Health Centre. Brampron Memorial Hospital, 20 Lynch Street, Brampton, Ontario, L6W 2Z8 Canada.<br><br>Chairperson- Henry Blair.
Trudeau, Maureen. MD	030067/029622	Odette Cancer Centre, Sunnybrook Health Sciences Centre, 2075 Bayview Avenue, Toronto, Ontario, M4N 3M5 Canada.	Research Ethics Board, Sunnybrook Health Sciences Centre, Room C819, 2075 Bayview Avenue, Toronto, Ontario M4N 3M5 Canada.<br><br>Chairperson- Philip Herbert.
Vergidis, Dimitrios MD	055711/032715	Thunder Bay Regional Healdl Science Centre, Regional cancer Care, 980 Oliver Road, Thunder Bay, ON P7B 6V8 Canada.	Ontario Cancer Research Ethics Board, Ontario Instinlle Cancer Research Network, MaRS Centre South Tower, 101 College Street. Suite 500, Toronto, Ontario MSG 1 L 7 Canada.<br><br>Chairperson- Jack Holland.

Investigator	Investigator no./Center no.	Description of Research Facility, Hospital/ Institution, and Address	Name of IEC/IRB Committee, Address, Committee Chair
**Chile**			
Gallardo, Jorge. MD	117838/039675	Instituto de Terapias Oncologicas Providencia, Drive Torres Boonan 520, Providencia, Santiago, 750-1088 Chile.	Comite Etico Cientiflco, Servicio de Salud Metropolitano del Oriente, Av Salvador 384, provideneill. Santiago, 750·0922 Chile.<br><br>Chairperson: Dr. Andrés Stuardo
Giannini, Osvaldo. MD	093892/030020	Cllnica Renaca, Anabaena 336, Vlna del Mar, 254-0364 Chile.	Comite Etico Cientlfico, Servicio de Salud Vlna del Mar - Quillota, Av, Alvarez 1532, Vlna del Mar, 254-0364, Chile.<br><br>Chairperson: Dr. Tulio Moreno Bolton.<br><br>Comite de Etica, Clinica Renaca, Anabaena 336, Vina del Mar, 254-0364, Chile.<br><br>Chairperson: Dr. Luis Bavestrello
Reyes, José Miguel. MD	036315/029920	Clinica Las Condes, Lo Fonteollla 441, Las Condes -Santiago, 877-0126 Chile.	Comite Etlco Cientifico, Servicio de Balud Metropolltano Oriente, Av. Salvador 364,

Investigator	Investigator no./Center no.	Description of Research Facility, Hospital/ Institution, and Address	Name of IEC/IRB Committee, Address, Committee Chair
			750-0922, Santiago.<br><br>Chairperson: Dr. Andrés Stuardo<br><br>Comite de Etioa, Clinica Las Condes, Lo Fonteollla 441, Las Condes -Santiago, 877-0128, Chile.<br><br>Chairperson: Armando Ortiz Pommier.
Salman, Pamela. MD	058834/029921	Fundacion Arturo Lopez Perez Rancagua 878-Providencia, Santiago, 750-0921 Chile.	Comite Etico Cientifico, Servicio de salud Metropolitano del Oriente, Av. Salvador 364, Providencia, Santiago, 750-0922 Chile.<br><br>Chairperson: Dr. Andrés Stuardo
**China**			
Jiang, Zefei. MD	083838/035210	The hospital affiliated to Military Medical Science Academy, No.8 East Road, Fengtai District, Beijing, 100071 P. R. China.	Ethics Committee of the Hospital affiliated to Military Medical, Science Academy, No.8 East Road, Fengtai District, Beijing 100071 P.

Investigator	Investigator no./Center no.	Description of Research Facility, Hospital/ Institution, and Address	Name of IEC/IRB Committee, Address, Committee Chair
			R. China.<br><br>Chairperson: Yuezhong He
Liu, Donggeng. MD	087321/035200	Sun Yat-Sen University Cancer Center, 651 Dongfeng Eastern Road, Guangzhou, 510060 China.	Ethics Committee of Sun Yat-Sen University Cancer Center, 651 Dongfeng Eastern Road, Guangzhou, 510060 China.<br><br>Chairperson: Wangqing Peng
Shen, Zhenzhou. MD	083832/035211	Cancer Hospital affilicated to Fudan university, 270 Dong An Road, Shanghai 200032 P.R.China.	Ethics Committee of Cancer Hospital affilicated to Fudan university, 270 Dong An Road, Shanghai 200032 P.R.China.<br><br>Chairperson: Jiong Wu.
Tong, Zhongsheng. MD	083837/035202	Tianjin Cancer Hospital, Huan, Huxi Road, Ti Yuan Bei Hexi District, Tianjing 300060, P.R.China.	Ethics Committee of Tianjin Cancer Hospital, Huan, Huxi Road, Ti Yuan Bei Hexi District, Tianjing 300060 P.R.China.<br><br>Chairperson: Ping Wang.

Investigator	Investigator no./Center no.	Description of Research Facility, Hospital/ Institution, and Address	Name of IEC/IRB Committee, Address, Committee Chair
Wang, Yongsheng. MD	113190/049773	The Cancer Hospital of Shandong Province, 440 Jiyan Road, Jinan, Shandong 25011 7 P.R.China	Ethics Committee of The Cancer Hospital of Shandong Province, 440Jiyan Road, Jinan, Shandong 25011 7 P.R.China.<br><br>Chairperson: Jinming Yu.
Xu, Binghe. MD, Ph.D	083840/035201	Cancer Hospital, CAMS&PUMC, 17 Pan Jia Yuan Nan Li, Chaoyang District, Beijing 100021 P.R.China.	Ethics Committee of Cancer Hospital, CAMS&PUMC, 17 Pan Jia Yuan Nan Li, Chaoyang District, Beijing 100021 P.R.China.<br><br>Chairperson: Datong Chu.
Yu, Shiying. MD	098501/035209	Huazhong University of Science and Technology Tongji Medical College Tongji Hospital 1095 Jie Fang DaDao, Wuhan, Hubei 430030 P.R.China.	Ethics Com mit tee of Huazhong University of Science and Technology Tongji Medical College Tongj i Hospital, 1095 Jie Fang DaDao, Wuhan, Hubei 430030 P.R.China.<br><br>Chairperson: Fandian Zeng.

Investigator	Investigator no./Center no.	Description of Research Facility, Hospital/ Institution, and Address	Name of IEC/IRB Committee, Address, Committee Chair
**Croatia**			
Grgic, Mislav. MD	049347/032301	KBC Zagreb, Klinika za onkologiju, Kispaticeva 12, Zagreb, 10000 Croatia.	Sredisnje eticko povjerenstvo za lijekove i rnedicinske proizvode, Ulica Roberta Frangeša Mihanovića 9, Zagreb, 10 000 Croatia.<br><br>Chairperson- Prof.dr.sc.Dinko Vitezić, dr.med.<br><br>Ministarstvo zdravstva Republike Hrvatske; Uprava za stručno-medicinske poslove, Odijel za lijekove; Ulica Roberta Frangeša Mihanovića 9, Zagreb, 10 000 Croatia.<br><br>Chairperson- Prof.dr.sc.Rajko Ostojić, dr.med.
Kotromanović, Zdenka. MD	082641/032304	KB Osijek, Odjel za onkologiju, Huttlerova 4, Osijek, 31000 Croatia.	Sredisnje eticko povjerenstvo za lijekove i rnedicinske proizvode, Ulica Roberta

Investigator	Investigator no./Center no.	Description of Research Facility, Hospital/ Institution, and Address	Name of IEC/IRB Committee, Address, Committee Chair
			Frangeša Mihanovića 9, Zagreb, 10 000 Croatia.<br><br>Chairperson- Prof.dr.sc.Dinko Vitezić, dr.med.<br><br>Ministarstvo zdravstva Republike Hrvatske; Uprava za stručno-medicinske poslove, Odijel za lijekove; Ulica Roberta Frangeša Mihanovića 9, 10 000 Zagreb, Croatia.<br>Chairperson- Prof. dr.sc. Rajko Ostojić, dr.med.

Investigator	Investigator no./Center no.	Description of Research Facility, Hospital/ Institution, and Address	Name of IEC/IRB Committee, Address, Committee Chair
Pavlović Ružić, Ira. MD	098639/032302	KBC Rijeka, Zavod za radioterapiju i onkologiju, KreCirnirava 42, Rijeka, 51000 Croatia.	Sredisnje eticko povjerenstvo za lijekove i rnedicinske proizvode, Ulica Roberta Frangeša Mihanovića 9, Zagreb, 10 000 Croatia.<br><br>Chairperson- Prof.dr.sc.Dinko Vitezić, dr.med.<br><br>Ministarstvo zdravstva Republike Hrvatske; Uprava za stručno-medicinske poslove, Odijel za lijekove; Ulica Roberta Frangeša Mihanovića 9, 10 000 Zagreb, Croatia. Chairperson- Prof.dr.sc.Rajko Ostojić, dr.med.
Radolović, Liliana. MD<br>Bozac, Mario. MD. (Former PI)	114353/032303	OB Pula, Djelatnost za unutarnje bolesti, Negrijeva 6, Pula, 52000 Croatia.	Sredisnje eticko povjerenstvo za lijekove i rnedicinske proizvode, Ulica Roberta Frangeša Mihanovića 9, Zagreb, 10 000 Croatia.

Investigator	Investigator no./Center no.	Description of Research Facility, Hospital/ Institution, and Address	Name of IEC/IRB Committee, Address, Committee Chair
			Chairperson- Prof.dr.sc.Dinko Vitezić, dr.med.<br><br>Ministarstvo zdravstva Republike Hrvatske; Uprava za stručno-medicinske poslove, Odijel za lijekove; Ulica Roberta Frangeša Mihanovića 9, Zagreb, 10 000 Croatia.<br><br>Chairperson- Prof.dr.sc.Rajko Ostojić, dr.med.
Vrdoljak, Eduard	030301/032299	KB Split, Centar za onkologiju Spinciceva 1, Split, 21000 Croatia.	Sredisnje eticko povjerenstvo za lijekove i medicinske proizvode, Ulica Roberta Frangeša Mihanovića 9, Zagreb, 10 000 Croatia.<br><br>Chairperson- Prof.dr.sc.Dinko Vitezić, dr.med.

Investigator	Investigator no./Center no.	Description of Research Facility, Hospital/ Institution, and Address	Name of IEC/IRB Committee, Address, Committee Chair
			Ministarstvo zdravstva Republike Hrvatske; Uprava za stručno-medicinske poslove, Odijel za lijekove; Ulica Roberta Frangeša Mihanovića 9, 10 000 Zagreb, Croatia. Chairperson- Prof.dr.sc.Rajko Ostojić, dr.med.
**Czech Republic**			
Petrakova, Katarina. MD	054796/031062	Masaryk Memorial Cancer Institute, Zluty kopec 7, Brno, 656 53 Czech Republic.	Multicenter Ethics Committee of General Faculty Hospital, Na Bojisti 1, Prague 2, 128 08 Czech Republic.<br><br>Chairperson- Josef Sedivy, MD<br><br>Ethics Committee of Masaryk Memorial Cancer Institute, Zluty kopec 7, Brno, 656 53 Czech Republic.<br><br>Chairperson- Oldrich Coufal, MD

Investigator	Investigator no./Center no.	Description of Research Facility, Hospital/ Institution, and Address	Name of IEC/IRB Committee, Address, Committee Chair
Petruzelka, Lubos. MD	000638/031065	Teaching Health Centre, Karlovo namesti 32, Prague 2, 12100 Czech Republic.	Multicenter Ethics Committee of General Faculty Hospital, Na Bojistl 1, Prague 2, 12808 Czech Republic.<br><br>Chairperson- Josef Sedivy, MD
Stahalova, Vladimira. MD		Institute of radiation oncology, Faculty Hospital Bulovka, Na Truhlarce 100, Prague 8, 18000 Czech Republic.	Multicenter Ethics Committee of General Faculty Hospital, Na Bojisti 1, Prague 2, 128 08 Czech Republic.<br><br>Chairperson- Josef Sedivy, MD<br><br>Ethics Committee of Faculty Hospital Bulovka, Budinova 2, Prague 8, 18081 Czech Republic.<br><br>Chairperson- Michal Holub, MD

Investigator	Investigator no./Center no.	Description of Research Facility, Hospital/ Institution, and Address	Name of IEC/IRB Committee, Address, Committee Chair
**Denmark**			
Jakobsen, Erik Hugger. MD	035503/035011	Department of Oncology, Vejle Sygehus, Kabbeltoft 25, Vejle, DK-7100 Denmark.	Den Videnskabsetiske Komite for Region Syddanmark, Odense Universitetshospita Kløvervaenget 10, 2. Odense C, 5000 Denmark.<br><br>Chairperson- Birger Møller.
Jensen, Brita Bjerregaard. MD	098406/031908	Onkologisk Klinik, Sydvestjysk Sygehus Esbjerg, Finsensgade 35, Esbjerg, 6700 Denmark.	Den Videnskabsetiske Komite for Region Syddanmark, Odense Universitetshospital Kløvervaenget 10, 2. Odense C, 5000 Denmark.<br><br>Chairperson- Birger Møller.
Knoop, Ann. MD	044605/031789	Onkologisk afdeling, Odense Universitetshospita, Sdr. Boulevard 29, Odense, 5000 Denmark.	Den Videnskabsetiske Komite for Region Syddanmark, Odense Universitetshospital Kløvervaenget 10, 2. Odense C, 5000 Denmark.<br><br>Chairperson- Birger Møller.

Investigator	Investigator no./Center no.	Description of Research Facility, Hospital/ Institution, and Address	Name of IEC/IRB Committee, Address, Committee Chair
Philip, Preben. MD	035495/050678	Naestved sygehus, Department of Oncology/Hematology, Ringstedgade 61, Naestved, DK-4700 Denmark.	Den Videnskabsetiske Komite for Region Syddanmark, Odense Universitetshospital Kløvervaenget 10, 2. Odense C, 5000 Denmark.<br><br>Chairperson- Birger Møller.
Stenbygaard, Lars. MD<br>Ewertz, Marianne.MD (Former PI)	049468/031793	Onkologisk Afdeling, Aalborg Sygehus Syd, Hobrovej 18-22, Aalborg, DK-9100 Denmark.	Den Videnskabsetiske Komite for Region Syddanmark, Odense Universitetshospital Kløvervaenget 10, 2. Odense C, 5000 Denmark.<br><br>Chairperson- Birger Møller.
Tange, Ulla. MD	050122/031791	The Finsen Center Rigshospitalet, Copenhagen University Hospital, Blegdamsvej 9, Copenhagen ø, DK-2100 Denmark.	Den Videnskabsetiske Komite for Region Syddanmark, Odense Universitetshospital Kløvervaenget 10, 2. Odense C, 5000 Denmark.<br><br>Chairperson- Birger Møller.

Investigator	Investigator no./Center no.	Description of Research Facility, Hospital/ Institution, and Address	Name of IEC/IRB Committee, Address, Committee Chair
Tuxen, Gosia. MD	104189/043379	Herlev Hospital, Department of Oncology, Herlev Ringvej 75, Herlev, DK- 2730 Denmark.	Den Videnskabsetiske Komite for Region Syddanmark, Odense Universitetshospital Kløvervaenget 10, 2. Odense C, 5000 Denmark.<br><br>Chairperson- Birger Møller.
Vestlev, Peter. MD.<br>Sorensen, Peter. MD (Former PI)	035969/031794	Department of Oncology and Haematology, Roskilde Hospital, Kogevej 7-13, Roskilde, DK-4000 Denmark.	Den Videnskabsetiske Komite for Region Syddanmark, Odense Universitetshospital Kløvervaenget 10, 2. Odense C, 5000 Denmark.<br><br>Chairperson- Birger Møller.
**France**			
Audhuy, Bruno. MD	002585/031237	Hopital Pasteur - Hopitaux civils Colmar, Service d'Onco-Hematologie et Immunologie Clinique 39, avenue de la Liberte, Colmar Cedex, 68 024 France.	CPP de Lyon Sud-Est IV, Centre Leon Berard 28 rue Laennec, Lyon cedex 08, 69373 France.<br><br>Chairperson- Daniel Espinouse, MD.

Investigator	Investigator no./Center no.	Description of Research Facility, Hospital/ Institution, and Address	Name of IEC/IRB Committee, Address, Committee Chair
Bachelot, Thomas. MD	037302/032615	Departement de Cancerologie Medicale, Centre Leon Berard, 28 rue Laennec, Lyon cedex 08, 69373 France.	CPP de Lyon Sud-Est IV, Centre Leon Berard 28 rue Laennec, Lyon cedex 08, 69373 France.<br><br>Chairperson- Daniel Espinouse, MD.
Blay, Jean-Yves.	060439/031331	Hopital Edouard Herriot, Pavillion E- Oncologie medicale 5 Place d'Arsonval, 69437 Lyon cedex 03, France.	CPP de Lyon Sud-Est IV, Centre Leon Berard 28 rue Laennec, Lyon cedex 08, 69373 France.<br><br>Chairperson- Daniel Espinouse, MD.
Bonneterre, Jacques. MD	062011/035069	Centre Oscar Lambret 3, rue Frederic Combemale, B.P. 307-59020 Lille, France.	CPP de Lyon Sud-Est IV, Centre Leon Berard 28 rue Laennec, Lyon cedex 08, 69373 France.<br><br>Chairperson- Daniel Espinouse, MD.
Campone, Mario. MD	037298/031274	Centre Rene Gauducheau (CRLCC Nantes), Service d'oncologie Medicale, Boulevard Jacques Monod, Saint Herblain cedex, 44 805	CPP de Lyon Sud-Est IV, Centre Leon Berard 28 rue Laennec, Lyon cedex 08, 69373 France.

Investigator	Investigator no./Center no.	Description of Research Facility, Hospital/ Institution, and Address	Name of IEC/IRB Committee, Address, Committee Chair
		France.	Chairperson- Daniel Espinouse, MD.
Chevelle, Christian. MD	104607/ 037958	Centre des Hautes Energies 6, rue Maspoul Toulouse, 31400 France.	CPP de Lyon Sud-Est IV, Centre Leon Berard 28 rue Laennec, Lyon cedex 08, 69373 France.<br><br>Chairperson- Daniel Espinouse, MD.
Chollet, Philippe. MD	086028/ 030863	CAC Jean Perrin, 58 rue Montalembert, Clermot-Ferrand, 63 000 France.	CPP de Lyon Sud-Est IV, Centre Leon Berard 28 rue Laennec, Lyon cedex 08, 69373 France.<br><br>Chairperson- Daniel Espinouse, MD.
Coudert, Bruno. MD	004637/ 031292	CRLCC Georges Francois Lecierc, Service d'Oncologie Medicale 1, rue du Proffesseur Marion-BP 77980 21079 Dijon cedex, France.	CPP de Lyon Sud-Est IV, Centre Leon Berard 28 rue Laennec, Lyon cedex 08, 69373 France.<br><br>Chairperson- Daniel Espinouse, MD.

Investigator	Investigator no./Center no.	Description of Research Facility, Hospital/ Institution, and Address	Name of IEC/IRB Committee, Address, Committee Chair
Delaloge, Suzette. MD	055396/030530	IGR, 39 rue Camille Desmoulins, Villejuif cedex, 94805 France.	CPP de Lyon Sud-Est IV, Centre Leon Berard 28 rue Laennec, Lyon cedex 08, 69373 France.<br><br>Chairperson- Daniel Espinouse, MD.
Delva, Remy. MD	001234/031227	Centre Paul Papin, Service d'oncologie Medical, 2 rue Moll, Angers cedex 1, 49033 France.	CPP de Lyon Sud-Est IV, Centre Leon Berard 28 rue Laennec, Lyon cedex 08, 69373 France.<br><br>Chairperson- Daniel Espinouse, MD.
Dourthe, Louis-Marie. MD	097457/033333	Cabinet d'Oncologie, 39 allee de la Robertsau, Strasbourg, 67 000 France.	CPP de Lyon Sud-Est IV, Centre Leon Berard 28 rue Laennec, Lyon cedex 08, 69373 France.<br><br>Chairperson- Daniel Espinouse, MD.
Extra, Jean-Marc	099019/032207	Institut Paoli Calmette, 232, boulevard Sainte Marguerite, BP 156, Marseille, 13273 France.	CPP de Lyon Sud-Est IV, Centre Leon Berard 28 rue Laennec, Lyon cedex 08, 69373 France.

Investigator	Investigator no./Center no.	Description of Research Facility, Hospital/ Institution, and Address	Name of IEC/IRB Committee, Address, Committee Chair
			Chairperson- Daniel Espinouse, MD.
Facchini, Thomas. MD	096551/031058	Polyclinique Courlancy, 38 rue de Courlancy, Reims, 51100 France.	CPP de Lyon Sud-Est IV, Centre Leon Berard 28 rue Laennec, Lyon cedex 08, 69373 France.<br><br>Chairperson- Daniel Espinouse, MD.
Ferrero, Jean-Marc. MD	040632/032507	Service d'Oncologie Medicale, CAC Antoine Lacassagne 33, avenue de Vatombrose, Nice, 06189 France.	CPP de Lyon Sud-Est IV, Centre Leon Berard 28 rue Laennec, Lyon cedex 08, 69373 France.<br><br>Chairperson- Daniel Espinouse, MD.
Gutierrez, Maya. MD	052996/030635	Centre Rene Hugenin, 35 r Daily, Saint Cloud, 92210 France.	CPP de Lyon Sud-Est IV, Centre Leon Berard 28 rue Laennec, Lyon cedex 08, 69373 France.<br><br>Chairperson- Daniel Espinouse, MD.

Investigator	Investigator no./Center no.	Description of Research Facility, Hospital/ Institution, and Address	Name of IEC/IRB Committee, Address, Committee Chair
Jaubert, Dominique. MD	096651/031139	Clinique Tivoli, 220 rue Mandron, Bordeaux, 33000 France.	CPP de Lyon Sud-Est IV, Centre Leon Berard 28 rue Laennec, Lyon cedex 08, 69373 France.<br><br>Chairperson- Daniel Espinouse, MD.
Kerbrat, Pierre.MD	096957/031284	Centre Eugene Marquis (CRLCC Rennes) Oncologie Medicale, Rue de la Bataille Flandres-Dunkerque-CS 44229 35042 Rennes cedex, France.	CPP de Lyon Sud-Est IV, Centre Leon Berard 28 rue Laennec, Lyon cedex 08, 69373 France.<br><br>Chairperson- Daniel Espinouse, MD.
Levy, Christelle. MD.<br>Delozier, Thierry.MD (Former PI)	050817/031239	CRLCC Francois Baclesse, Comite Pathologies Mammaries, Avenue du general Harris, BP 5026, 14076 Caen cedex 05, France.	CPP de Lyon Sud-Est IV, Centre Leon Berard 28 rue Laennec, Lyon cedex 08, 69373 France.<br><br>Chairperson- Daniel Espinouse, MD.
Lortholary, Alain.MD	067974/031565	Clinique Catherine de Sienne, Service de Cancerologie, 2 rue Eric Tabarly- BP 20215, 44202 Nantes cedex 2, France.	CPP de Lyon Sud-Est IV, Centre Leon Berard 28 rue Laennec, Lyon cedex 08, 69373 France.

Investigator	Investigator no./Center no.	Description of Research Facility, Hospital/ Institution, and Address	Name of IEC/IRB Committee, Address, Committee Chair
			Chairperson- Daniel Espinouse, MD.
Luporsi, Elisabeth. MD	050378/045545	CRLCC Alexis Vautrin, Service Medecine Oncologique, Avenue de Bourgogne, 54511 Vandoeuvre-Les-Nancy, France.	CPP de Lyon Sud-Est IV, Centre Leon Berard 28 rue Laennec, Lyon cedex 08, 69373 France.<br><br>Chairperson- Daniel Espinouse, MD.
Machover, David. MD	105250/ 032346	Hopital Paul Brousse, Service Oncologie Medicale 12, avenue Paul-Vaillant-Couturier, 94000 Villejuif, France.	CPP de Lyon Sud-Est IV, Centre Leon Berard 28 rue Laennec, Lyon cedex 08, 69373 France.<br><br>Chairperson- Daniel Espinouse, MD.
Medionio, Jacques. MD	062009/030634	Hopital European Georges Pompidou, Service de cancerologie Medicale, 20 rue Leblanc,Paris Cedex 15, 75908 France.	CPP de Lyon Sud-Est IV, Centre Leon Berard 28 rue Laennec, Lyon cedex 08, 69373 France.<br><br>Chairperson- Daniel Espinouse, MD.
Miglianico, Laurent. MD	096958/031285	Centre hospitalier prive Saint Gregoire, 6 bvd	CPP de Lyon Sud-Est IV, Centre Leon Berard

Investigator	Investigator no./Center no.	Description of Research Facility, Hospital/ Institution, and Address	Name of IEC/IRB Committee, Address, Committee Chair
		Boutiere- CS 56816, Saint Gregoire, 35768 France.	28 rue Laennec, Lyon cedex 08, 69373 France.<br><br>Chairperson- Daniel Espinouse, MD.
Monnier, Alain.MD	097837/031616	Le Mittan, Boulevard do Marechal Juin, Montbeliard, 25 200 France.	CPP de Lyon Sud-Est IV, Centre Leon Berard 28 rue Laennec, Lyon cedex 08, 69373 France.<br><br>Chairperson- Daniel Espinouse, MD.
Pivot, Xavier.MD	049858/031290	Service d'Oncologie, CHU Besancon, 3 boulevard Fleming, Besancon, 25030 France.	CPP de Lyon Sud-Est IV, Centre Leon Berard 28 rue Laennec, Lyon cedex 08, 69373 France.<br><br>Chairperson- Daniel Espinouse, MD.
Rimailho, Jacques. MD	053292/ 036990	Service de Chirurgie Gynecologique, Bat H2 7eme etage CHU de Rangueil 1, avenue Jean Poulhes TSA 50032, Toulouse cedex 9, 31059 France.	CPP de Lyon Sud-Est IV, Centre Leon Berard 28 rue Laennec, Lyon cedex 08, 69373 France.

Investigator	Investigator no./Center no.	Description of Research Facility, Hospital/ Institution, and Address	Name of IEC/IRB Committee, Address, Committee Chair
			Chairperson- Daniel Espinouse, MD.
Rohart de Cordoue, Sylvie. MD	103568/034223	Centre Bourgogne Service Oncologie Medicale, 144 avenue de Dunkerque, Lille, 59 000 France.	CPP de Lyon Sud-Est IV, Centre Leon Berard 28 rue Laennec, Lyon cedex 08, 69373 France.<br><br>Chairperson- Daniel Espinouse, MD.
Romieu, Gilles. MD	044034/033776	CRLCC Val d'Aurelle Paul Lamarque, 326 rue des Apothicaires, Parc Euromedecine, Montpellier cedex 5, 34298 France.	CPP de Lyon Sud-Est IV, Centre Leon Berard 28 rue Laennec, Lyon cedex 08, 69373 France.<br><br>Chairperson- Daniel Espinouse, MD.
Rouverand, Nicole. MD	099238/032313	Clinique de la Victorie Service d'Oncologie 1, Quai du Havre, Tourcoing, 59200 France.	CPP de Lyon Sud-Est IV, Centre Leon Berard 28 rue Laennec, Lyon cedex 08, 69373 France.

Investigator	Investigator no./Center no.	Description of Research Facility, Hospital/ Institution, and Address	Name of IEC/IRB Committee, Address, Committee Chair
			Chairperson- Daniel Espinouse, MD.
**Germany**			
Abenhardt, Wolfgang*	049379/030349	Dr. Med Wolfgang Abenhardt Gemeinschaftspraxis, Haematologie und internistische Onkologie, Prielmayerstr. 1, Muenchen, 80335 Germany.	Ethik-Kommission der Med. Fakultaet der LMU Muenchen, Prof. Dr. med. Gustav Paumgartner, Klinikum der Universitaet Grosshadern Marchioninistreet 15, Muenchen, 81377 Germany.<br><br>Chairperson- Prof. Dr. Wolfgang Eisenmenger
Augustin, Doris. Dr.med	109171/039149	Dr. med. Doris Audustin, Klinikum Deggendorf, Mammazentrum Perlasber Street. 41, Deggendorf, 94469 Germany.	Ethik-Kommission der Med. Fakultaet der LMU Muenchen, Prof. Dr. med. Gustav Paumgartner, Klinikum der Universitaet Grosshadern Marchioninistreet 15, Muenchen, 81377 Germany.<br><br>Chairperson- Prof. Dr. Wolfgang Eisenmenger

Investigator	Investigator no./Center no.	Description of Research Facility, Hospital/ Institution, and Address	Name of IEC/IRB Committee, Address, Committee Chair
Bauerfeind, Ingo. Dr.med*	098188/031944	Dr. med. Ingo Bauerfeind, Klinikurn der Universitaet Muenchen – Grosshadern, Klinik und Poliklinik fuer, Frauenheilkunde und Geburtshilfe Marchioninistreet 15, Muenchen, 81377 Germany.	Ethik-Kommission der Med. Fakultaet der LMU Muenchen, Prof. Dr. med. Gustav Paumgartner, Klinikum der Universitaet Grosshadern Marchioninistreet 15, Muenchen, 81377 Germany.<br><br>Chairperson- Prof. Dr. Wolfgang Eisenmenger
Bechtner, Christina. Dr.med<br>Maerz, Wolfgang. Dr.med (Former PI)	012998//030416	Dr. med. Christina Benchtner Klinikum Memmingen, Bismarckstr. 23, Memmingen, 87700 Germany.	Ethik-Kommission der Med. Fakultaet der LMU Muenchen, Prof. Dr. med. Gustav Paumgartner, Klinikum der Universitaet Grosshadern Marchioninistreet 15, Muenchen, 81377 Germany.<br><br>Chairperson- Prof. Dr. Wolfgang Eisenmenger
Beha, Michaela. Dr.med	098190/031946	Dr. med. Michaela Beha, Klinikum Saint. Marien Amberg Brustzentrum Amberg,	Ethik-Kommission der Med. Fakultaet der LMU Muenchen, Prof. Dr. med. Gustav

Investigator	Investigator no./Center no.	Description of Research Facility, Hospital/ Institution, and Address	Name of IEC/IRB Committee, Address, Committee Chair
		Mariahilfbergweg 5-7, Amberg, 92224 Germany.	Paumgartner, Klinikum der Universitaet Grosshadern Marchioninistreet 15, Muenchen, 81377 Germany.<br><br>Chairperson- Prof. Dr. Wolfgang Eisenmenger
Beldermann, Frank. Dr.med	091040/030351	Dr. med. Frank Beldermann Gemeinschaftapaxis Dres. Beldermann und Kuhn<br>Werderstr. 66, Stuttgart, 70190 Germany.	Ethik-Kommission der Med. Fakultaet der LMU Muenchen, Prof. Dr. med. Gustav Paumgartner, Klinikum der Universitaet Grosshadern Marchioninistreet 15, Muenchen, 81377 Germany.<br><br>Chairperson- Prof. Dr. Wolfgang Eisenmenger
Bischoff, Joachim. Dr.med	083042/030356	Dr. med Joachim Bischoff, Universitaetsfrauenklinik Magdeburg, Gerhart-hauptmann-Strasse 35, Magdeburg, 39108 Germany.	Ethik-Kommission der Med. Fakultaet der LMU Muenchen, Prof. Dr. med. Gustav Paumgartner, Klinikum der Universitaet Grosshadern Marchioninistreet 15,

Investigator	Investigator no./Center no.	Description of Research Facility, Hospital/ Institution, and Address	Name of IEC/IRB Committee, Address, Committee Chair
			Muenchen, 81377 Germany.<br><br>Chairperson- Prof. Dr. Wolfgang Eisenmenger
Bittl, Andreas. Dr.med	091044/030357	Dr. med. Andreas Bittl Gemeinschaftspaxis Dres. Bittl, Klein, Horn, Gruener und Wieland Weinbergweg 16, Roth, 91154 Germany.	Ethik-Kommission der Med. Fakultaet der LMU Muenchen, Prof. Dr. med. Gustav Paumgartner, Klinikum der Universitaet Grosshadern Marchioninistreet 15, Muenchen, 81377 Germany.<br><br>Chairperson- Prof. Dr. Wolfgang Eisenmenger
Conrad, Bettina. Dr.med	109187/070668	Dr. med. Bettina Conrad, Elisabeth Krankenhaus GmbH, Brustzentrum Kassel, Weinbergstreet 7, Kassel, 34117 Germany.	Ethik-Kommission der Med. Fakultaet der LMU Muenchen, Prof. Dr. med. Gustav Paumgartner, Klinikum der Universitaet Grosshadern Marchioninistreet 15, Muenchen, 81377 Germany.

Investigator	Investigator no./Center no.	Description of Research Facility, Hospital/ Institution, and Address	Name of IEC/IRB Committee, Address, Committee Chair
			Chairperson- Prof. Dr. Wolfgang Eisenmenger
Cordes, Hans-Joerg. Dr.med*	063138/030360	Dr. med. Hans-Joerg Cordes, Vitanos GmbH, Internistisches Facharztzentrum Stresemannallee 3, Frankfurt a.M, 60596 Germany.	Ethik-Kommission der Med. Fakultaet der LMU Muenchen, Prof. Dr. med. Gustav Paumgartner, Klinikum der Universitaet Grosshadern Marchioninistreet 15, Muenchen, 81377 Germany.<br><br>Chairperson- Prof. Dr. Wolfgang Eisenmenger
Deertz, Holger. Dr.med	111788/050710	Dr. med. Holger Deertz, Praxis Dr. med. H. Deertz, Ruettenscheider Street 56, Essen, 45130 Germany.	Ethik-Kommission der Med. Fakultaet der LMU Muenchen, Prof. Dr. med. Gustav Paumgartner, Klinikum der Universitaet Grosshadern Marchioninistreet 15, Muenchen, 81377 Germany.<br><br>Chairperson- Prof. Dr. Wolfgang Eisenmenger

Investigator	Investigator no./Center no.	Description of Research Facility, Hospital/ Institution, and Address	Name of IEC/IRB Committee, Address, Committee Chair
Deryal, Mustafa. Dr.med<br>Kirschbaum, Michael. Dr.med (Former PI)	140327/030409	Dr. med. Mustafa Deryal, Caritasklinik Street. Theresia – Frauenklinik, Brustzentrum Saar Mitte, Rheinstr. 2, Saarbruecken, 66113 Germany.	Ethik-Kommission der Med. Fakultaet der LMU Muenchen, Prof. Dr. med. Gustav Paumgartner, Klinikum der Universitaet Grosshadern Marchioninistreet 15, Muenchen, 81377 Germany.<br><br>Chairperson- Prof. Dr. Wolfgang Eisenmenger
Diedrich, Klaus. Dr.med	095230/031778	Universitaetsklinikum Schleswig-Holstein, Campus Luebeck Klinikum fuer Frauenheilkunde und Geburtshilfe, Ratzeburger Allee 160, Luebeck, 23558 Germany.	Ethik-Kommission der Med. Fakultaet der LMU Muenchen, Prof. Dr. med. Gustav Paumgartner, Klinikum der Universitaet Grosshadern Marchioninistreet 15, Muenchen, 81377 Germany.<br><br>Chairperson- Prof. Dr. Wolfgang Eisenmenger

Investigator	Investigator no./Center no.	Description of Research Facility, Hospital/ Institution, and Address	Name of IEC/IRB Committee, Address, Committee Chair
Dietrich, Maria. Dr.med	091136/030361	Frau. Dr. med. Maria Dietrich Klinikum Fichtelgebirge Marktredwitz, Brustzentrum Weiden-Marktredwitz, Am Schillerhain 1-8, Marktredwitz, 95615 Germany.	Ethik-Kommission der Med. Fakultaet der LMU Muenchen, Prof. Dr. med. Gustav Paumgartner, Klinikum der Universitaet Grosshadern Marchioninistreet 15, Muenchen, 81377 Germany.<br><br>Chairperson- Prof. Dr. Wolfgang Eisenmenger
Dietz, Wolfgang. Dr.med	091140/030362	Dr. med. Wolfgang Dietz, Praxis Dr. med. Wolfgang Dietz, Albert-Schweitzer-Str. 18, Salzgitter, 38226 Germany.	Ethik-Kommission der Med. Fakultaet der LMU Muenchen, Prof. Dr. med. Gustav Paumgartner, Klinikum der Universitaet Grosshadern Marchioninistreet 15, Muenchen, 81377 Germany.<br><br>Chairperson- Prof. Dr. Wolfgang Eisenmenger
Doering, Gabriele. Dr.med	111792/050371	Dr. med. Gabriele Doering Haematologisch-Onkologische Praxis,	Ethik-Kommission der Med. Fakultaet der LMU Muenchen, Prof. Dr. med. Gustav

Investigator	Investigator no./Center no.	Description of Research Facility, Hospital/ Institution, and Address	Name of IEC/IRB Committee, Address, Committee Chair
		Schwachhauser Heerstr. 54, 2 Bremen, 8209 Germany.	Paumgartner, Klinikum der Universitaet Grosshadern Marchioninistreet 15, Muenchen, 81377 Germany.<br><br>Chairperson- Prof. Dr. Wolfgang Eisenmenger
Eiermann, Wolfgang. Dr.med	005905/031780	Prof. Dr. med. Wolfgang Eiermann, Frauenklinik vom Roten Kreuz, I. gynaekologisch-geburtshilfliche Abteilung, Taxisstrasse 3, Muenchen, 80637 Germany.	Ethik-Kommission der Med. Fakultaet der LMU Muenchen, Prof. Dr. med. Gustav Paumgartner, Klinikum der Universitaet Grosshadern Marchioninistreet 15, Muenchen, 81377 Germany.<br><br>Chairperson- Prof. Dr. Wolfgang Eisenmenger
Eschenburg, Henning. Dr.med	091144/031786	Dr. med. Henning Eschenburg Praxis Dr. med. Henning Eschenburg, Am Wall 1, Guestrow, 18273 Germany.	Ethik-Kommission der Med. Fakultaet der LMU Muenchen, Prof. Dr. med. Gustav Paumgartner, Klinikum der Universitaet Grosshadern Marchioninistreet 15,

Investigator	Investigator no./Center no.	Description of Research Facility, Hospital/ Institution, and Address	Name of IEC/IRB Committee, Address, Committee Chair
			Muenchen, 81377 Germany.<br><br>Chairperson- Prof. Dr. Wolfgang Eisenmenger
Fasching, Peter A. Dr.med	054327/030395	Dr. rned. Peter A. Fasching, Frauenklinik Universitaetsklinikurn Erlangen, Universitaets-Brustzentrum Franken Universitaetsstrasse 21 -23, Erlangen, 91054 Germany.	Ethik-Kommission der Med. Fakultaet der LMU Muenchen, Prof. Dr. med. Gustav Paumgartner, Klinikum der Universitaet Grosshadern Marchioninistreet 15, Muenchen, 81377 Germany.<br><br>Chairperson- Prof. Dr. Wolfgang Eisenmenger
Fehm, Tanja. Dr.med<br>Marme, Alexander. Dr, med (Former PI)<br>Huober, Jens. Dr.med (Former PI)	121197/031970	Prof. Dr. med. Tanja Fehm, Universitaetsfrauenklinik Tuebingen, Universitaets-Brustzentrum Tuebingen, Calwerstrasse 7, Tuebingen, 72076 Germany.	Ethik-Kommission der Med. Fakultaet der LMU Muenchen, Prof. Dr. med. Gustav Paumgartner, Klinikum der Universitaet Grosshadern Marchioninistreet 15, Muenchen, 81377 Germany.

Investigator	Investigator no./Center no.	Description of Research Facility, Hospital/ Institution, and Address	Name of IEC/IRB Committee, Address, Committee Chair
			Chairperson- Prof. Dr. Wolfgang Eisenmenger
Felderbaum, Ricardo E. Dr.med	091152/031788	Prof. Dr. med. Ricardo E. Felberbaum, Klinikum Kempten-Oberallgaeu GmbH, Interdisziplinaeres Brustzentrum Kempten - Allgaeu (IBZK-A), Robert-Weixler-Strasse 50, 87439 Kempten, Germany.	Ethik-Kommission der Med. Fakultaet der LMU Muenchen, Prof. Dr. med. Gustav Paumgartner, Klinikum der Universitaet Grosshadern Marchioninistreet 15, Muenchen, 81377 Germany.<br><br>Chairperson- Prof. Dr. Wolfgang Eisenmenger
Fietz, Thomas	119128/ 040226	Dr. Thomas Fietz, Gem. Praxis Drs. Banhardt und Fietz, Virchowstreet 10c, Singen, 78224 Germany.	Ethik-Kommission der Med. Fakultaet der LMU Muenchen, Prof. Dr. med. Gustav Paumgartner, Klinikum der Universitaet Grosshadern Marchioninistreet 15, Muenchen, 81377 Germany.<br><br>Chairperson- Prof. Dr. Wolfgang Eisenmenger

Investigator	Investigator no./Center no.	Description of Research Facility, Hospital/ Institution, and Address	Name of IEC/IRB Committee, Address, Committee Chair
Forstbauer, Helmut. Dr.med	063225/030396	Dr. med. Helmut Forstbauer, GOSPL (Gesellschaft fuer Onkologische Studien und Privatliquidation GbR) Schlosssteet 18, Troisdorf, 53840 Germany.	Ethik-Kommission der Med. Fakultaet der LMU Muenchen, Prof. Dr. med. Gustav Paumgartner, Klinikum der Universitaet Grosshadern Marchioninistreet 15, Muenchen, 81377 Germany.<br><br>Chairperson- Prof. Dr. Wolfgang Eisenmenger
Fricke, Hans Christian. Dr.med	116556/038554	Klinikum Konstanz Luisenstr. 7, Konstanz, 78464 Germany.	Ethik-Kommission der Med. Fakultaet der LMU Muenchen, Prof. Dr. med. Gustav Paumgartner, Klinikum der Universitaet Grosshadern Marchioninistreet 15, Muenchen, 81377 Germany.<br><br>Chairperson- Prof. Dr. Wolfgang Eisenmenger
Fuxuis, Stefan. Dr.med	062885/030397	Dr. med. Stefan Fuxius Gemeinschaftspraxis Dres. Fuxius und Karcher, Kurfuerstenanlage	Ethik-Kommission der Med. Fakultaet der LMU Muenchen, Prof. Dr. med. Gustav

Investigator	Investigator no./Center no.	Description of Research Facility, Hospital/ Institution, and Address	Name of IEC/IRB Committee, Address, Committee Chair
		34, Heidelberg, 69115 Germany.	Paumgartner, Klinikum der Universitaet Grosshadern Marchioninistreet 15, Muenchen, 81377 Germany.<br><br>Chairperson- Prof. Dr. Wolfgang Eisenmenger
Geberth, Matthias. Dr.med<br>Chatsiproios, Dimitrios. Dr.med (Former PI)	125202/050370	CGG Klinik GmbH Quadrat P7, 16-17 Mannheim, 68161 Germany.	Ethik-Kommission der Med. Fakultaet der LMU Muenchen, Prof. Dr. med. Gustav Paumgartner, Klinikum der Universitaet Grosshadern Marchioninistreet 15, Muenchen, 81377 Germany.<br><br>Chairperson- Prof. Dr. Wolfgang Eisenmenger
Goerke, Kay. Dr.med*	064985/030398	Dr. med. Kay Goerke, Krankenhaus Schwetzingen, Bodelschwinghstreet 10, Schwetzingen, 68723 Germany.	Ethik-Kommission der Med. Fakultaet der LMU Muenchen, Prof. Dr. med. Gustav Paumgartner, Klinikum der Universitaet Grosshadern Marchioninistreet 15,

Investigator	Investigator no./Center no.	Description of Research Facility, Hospital/ Institution, and Address	Name of IEC/IRB Committee, Address, Committee Chair
			Muenchen, 81377 Germany.<br><br>Chairperson- Prof. Dr. Wolfgang Eisenmenger
Graffunder, Gerd. Dr.med	091156/030399	Gerd Graffunder, F rauenarzt -Zentrum-Zehlendorf, Teltower Damm 7, Berlin, 14169 Germany.	Ethik-Kommission der Med. Fakultaet der LMU Muenchen, Prof. Dr. med. Gustav Paumgartner, Klinikum der Universitaet Grosshadern Marchioninistreet 15, Muenchen, 81377 Germany.<br><br>Chairperson- Prof. Dr. Wolfgang Eisenmenger
Grunewald, Ralf. Dr.med	091158/030400	Onkologlsche Gemeinschaftspraxis, 1m Pruefling 17-19, Frankfurt a. M., 60389 Germany.	Ethik-Kommission der Med. Fakultaet der LMU Muenchen, Prof. Dr. med. Gustav Paumgartner, Klinikum der Universitaet Grosshadern Marchioninistreet 15, Muenchen, 81377 Germany.

Investigator	Investigator no./Center no.	Description of Research Facility, Hospital/ Institution, and Address	Name of IEC/IRB Committee, Address, Committee Chair
			Chairperson- Prof. Dr. Wolfgang Eisenmenger
Guth, Dagmar. Dr.med	117498/039528	Dr. med. Dagmar Guth, Plauener Street 33 A, Plauen-Kauschwitz, 08525 Germany.	Ethik-Kommission der Med. Fakultaet der LMU Muenchen, Prof. Dr. med. Gustav Paumgartner, Klinikum der Universitaet Grosshadern Marchioninistreet 15, Muenchen, 81377 Germany.<br><br>Chairperson- Prof. Dr. Wolfgang Eisenmenger
Hackmann, John. Dr.med	091159/030401	Herr Dr. med. John Hackmann, Marienhospital Witten, Brustzentrum Marienplatz 2, Witten, 58452 Germany.	Ethik-Kommission der Med. Fakultaet der LMU Muenchen, Prof. Dr. med. Gustav Paumgartner, Klinikum der Universitaet Grosshadern Marchioninistreet 15, Muenchen, 81377 Germany.<br><br>Chairperson- Prof. Dr. Wolfgang Eisenmenger

Investigator	Investigator no./Center no.	Description of Research Facility, Hospital/ Institution, and Address	Name of IEC/IRB Committee, Address, Committee Chair
Hahn, Lars. Dr.med	072969/030402	Dr. med. Lars Hahn, Gemeinschaftspraxis Dres. Schumann, Reinhardt und Hahn, Wiescherstreet 20, Herne, 44623 Germany.	Ethik-Kommission der Med. Fakultaet der LMU Muenchen, Prof. Dr. med. Gustav Paumgartner, Klinikum der Universitaet Grosshadern Marchioninistreet 15, Muenchen, 81377 Germany.<br><br>Chairperson- Prof. Dr. Wolfgang Eisenmenger
Hanf, Volker. Dr.med	091160/030403	Prof. Dr. med. Volker Hanf, Staedtisches Klinikum Fuerth, Brustzentrurn, Jakob-Henle-Street 1, Fuerth, 90766 Germany.	Ethik-Kommission der Med. Fakultaet der LMU Muenchen, Prof. Dr. med. Gustav Paumgartner, Klinikum der Universitaet Grosshadern Marchioninistreet 15, Muenchen, 81377 Germany.<br><br>Chairperson- Prof. Dr. Wolfgang Eisenmenger
Heinig, Karin. Dr.med	117953/039745	Dr. med. Karin Heinig, Arztpraxis fuer Frauenheilkunde, Seilergasse 1, Sprernberg,	Ethik-Kommission der Med. Fakultaet der LMU Muenchen, Prof. Dr. med. Gustav

Investigator	Investigator no./Center no.	Description of Research Facility, Hospital/ Institution, and Address	Name of IEC/IRB Committee, Address, Committee Chair
		03130 Germany.	Paumgartner, Klinikum der Universitaet Grosshadern Marchioninistreet 15, Muenchen, 81377 Germany.<br><br>Chairperson- Prof. Dr. Wolfgang Eisenmenger
Heinrich, Georg. Dr.med	038214/030405	Dr. med. Georg Heinrich, Schwerpunktpraxis fuer Gynaekologische Onkologie Domgasse 1, Fuerstenwalde, 15517 Germany.	Ethik-Kommission der Med. Fakultaet der LMU Muenchen, Prof. Dr. med. Gustav Paumgartner, Klinikum der Universitaet Grosshadern Marchioninistreet 15, Muenchen, 81377 Germany.<br><br>Chairperson- Prof. Dr. Wolfgang Eisenmenger
Hempel, Dirk. Dr.med*	064427/030406	Dr. med. Dirk Hempel, Praxis Dr. Hernpel, Bergstr. 9a, Rehling, 86508 Germany.	Ethik-Kommission der Med. Fakultaet der LMU Muenchen, Prof. Dr. med. Gustav Paumgartner, Klinikum der Universitaet Grosshadern Marchioninistreet 15,

Investigator	Investigator no./Center no.	Description of Research Facility, Hospital/ Institution, and Address	Name of IEC/IRB Committee, Address, Committee Chair
			Muenchen, 81377 Germany.<br><br>Chairperson- Prof. Dr. Wolfgang Eisenmenger
Herwig, Uwe. Dr.med<br>Carstensen, Martin. Dr.med (Former PI)	093597/030359	Dr. med. Uwe Herwig, Albertinen Krankenhaus, Hamburg 8uentelstrasse 11 a, Hamburg, 22457 Germany.	Ethik-Kommission der Med. Fakultaet der LMU Muenchen, Prof. Dr. med. Gustav Paumgartner, Klinikum der Universitaet Grosshadern Marchioninistreet 15, Muenchen, 81377 Germany.<br><br>Chairperson- Prof. Dr. Wolfgang Eisenmenger
Hoess, Cornelia.PD. Dr.med	091014/033515	PD Dr. med. Comelia Hoess, Kreisklinik Ebersberg GmbH, Brustzentrum Rosenheim – Ebersberg, Pfarrer-Guggetzer Street 3, Ebersberg, 85560 Germany.	Ethik-Kommission der Med. Fakultaet der LMU Muenchen, Prof. Dr. med. Gustav Paumgartner, Klinikum der Universitaet Grosshadern Marchioninistreet 15, Muenchen, 81377 Germany.

Investigator	Investigator no./Center no.	Description of Research Facility, Hospital/ Institution, and Address	Name of IEC/IRB Committee, Address, Committee Chair
			Chairperson- Prof. Dr. Wolfgang Eisenmenger
Huebner, Jutta. Dr.med	119133/040227	Dr. med. Jutta Huebner, Gemeinschaftspraxis am Rhoenplatz, Standort Onkologie, Wigandstreet 1, Kassel, 34131 Germany.	Ethik-Kommission der Med. Fakultaet der LMU Muenchen, Prof. Dr. med. Gustav Paumgartner, Klinikum der Universitaet Grosshadern Marchioninistreet 15, Muenchen, 81377 Germany.<br><br>Chairperson- Prof. Dr. Wolfgang Eisenmenger
Jueckstock, Julia. Dr.med<br>Sommer, Harald. Dr. med (Former PI)<br>Janni, Wolfgang. Dr.med (Former PI)	096658/030348	Prof. Dr.med. Harald Sommer, Klinikum der Universitaet Muenchen, Frauenheilkunde, Maistreet 11, Muenchen, 80337 Germany.	Ethik-Kommission der Med. Fakultaet der LMU Muenchen, Prof. Dr. med. Gustav Paumgartner, Klinikum der Universitaet Grosshadern Marchioninistreet 15, Muenchen, 81377 Germany.<br><br>Chairperson- Prof. Dr. Wolfgang Eisenmenger

Investigator	Investigator no./Center no.	Description of Research Facility, Hospital/ Institution, and Address	Name of IEC/IRB Committee, Address, Committee Chair
Kast, Karin<br>Distler, Wolfgang. Dr.med<br>(Former PI)	095796/030363	Dr. med. Karin Kast, Universitatsklinikum Carl Gustav Carus, Regionales Brustzentrum Dresden, Fetscherstrasse 74, Dresden, 01307 Germany.	Ethik-Kommission der Med. Fakultaet der LMU Muenchen, Prof. Dr. med. Gustav Paumgartner, Klinikum der Universitaet Grosshadern Marchioninistreet 15, Muenchen, 81377 Germany.<br><br>Chairperson- Prof. Dr. Wolfgang Eisenmenger
Klare, Peter. Dr.med	040459/036075	Dr. med. Peter Klare, Praxisklinik Krebsheilkunde fuer Frauen, Moellendorffstreet 52, Berlin, 10367 Germany.	Ethik-Kommission der Med. Fakultaet der LMU Muenchen, Prof. Dr. med. Gustav Paumgartner, Klinikum der Universitaet Grosshadern Marchioninistreet 15, Muenchen, 81377 Germany.<br><br>Chairperson- Prof. Dr. Wolfgang Eisenmenger
Ko, Yon-Dschun Dr.med	032777/031972	Prof. Dr. med. Yon-Dschun Ko, Johanniter Krankenhaus, Innere Medizin I,	Ethik-Kommission der Med. Fakultaet der LMU Muenchen, Prof. Dr. med. Gustav

Investigator	Investigator no./Center no.	Description of Research Facility, Hospital/ Institution, and Address	Name of IEC/IRB Committee, Address, Committee Chair
		Johanniterstreet 3 – 5, Bonn, 53113 Germany.	Paumgartner, Klinikum der Universitaet Grosshadern Marchioninistreet 15, Muenchen, 81377 Germany.<br><br>Chairperson- Prof. Dr. Wolfgang Eisenmenger
Krabisch, Petra. Dr.med<br>Steck, Thomas. Dr.med (Former PI)	055393/031811	Dr. med. Petra Krabisch, Klinikum Chemnitz gGmbH, Brustzentrum, Flemmingstrabe 4, Chemnitz, 09116 Germany.	Ethik-Kommission der Med. Fakultaet der LMU Muenchen, Prof. Dr. med. Gustav Paumgartner, Klinikum der Universitaet Grosshadern Marchioninistreet 15, Muenchen, 81377 Germany.<br><br>Chairperson- Prof. Dr. Wolfgang Eisenmenger
Krafft, Winfried. Dr.med	112539/037387	Prof. Dr. med. Winfried Krafft, Kleine Loebergasse 1, Eisenach, 99817 Germany.	Ethik-Kommission der Med. Fakultaet der LMU Muenchen, Prof. Dr. med. Gustav Paumgartner, Klinikum der Universitaet Grosshadern Marchioninistreet 15,

Investigator	Investigator no./Center no.	Description of Research Facility, Hospital/ Institution, and Address	Name of IEC/IRB Committee, Address, Committee Chair
			Muenchen, 81377 Germany.<br><br>Chairperson- Prof. Dr. Wolfgang Eisenmenger
Kreienberg, Rolf	037503/031796	Universitaetsklinikum Ulm, Prittwitzstreet 43, Ulm, Baden-Wuerttemberg, 89075 Germany.	Ethik-Kommission der Med. Fakultaet der LMU Muenchen, Prof. Dr. med. Gustav Paumgartner, Klinikum der Universitaet Grosshadern Marchioninistreet 15, Muenchen, 81377 Germany.<br><br>Chairperson- Prof. Dr. Wolfgang Eisenmenger
Kreisel-Buestgens, Christiane. Dr.med	091026/030410	Dr. med. Christiane Kreisel-Buestgens, Praxis Dr. med. Christiane Kreisel- Buestgens, Flurweg 13, Porta Westfellica, 32457 Germany.	Ethik-Kommission der Med. Fakultaet der LMU Muenchen, Prof. Dr. med. Gustav Paumgartner, Klinikum der Universitaet Grosshadern Marchioninistreet 15, Muenchen, 81377 Germany.

Investigator	Investigator no./Center no.	Description of Research Facility, Hospital/ Institution, and Address	Name of IEC/IRB Committee, Address, Committee Chair
			Chairperson- Prof. Dr. Wolfgang Eisenmenger
Kroening, Hendrik. Dr.med	091029/031797	Dr. med. Hendrik Kroening, Hasselbachplatz 2, Magdeburg, 39104 Germany.	Ethik-Kommission der Med. Fakultaet der LMU Muenchen, Prof. Dr. med. Gustav Paumgartner, Klinikum der Universitaet Grosshadern Marchioninistreet 15, Muenchen, 81377 Germany.<br><br>Chairperson- Prof. Dr. Wolfgang Eisenmenger
Landthaler, Robert. Dr.med	116540/039150	Dr. med. Robert Landthaler, Gemeinschaftspraxis, Dres. Landthaler und Litschmann, Mindelheimerstreet 69, 86381 Krumbach, Germany.	Ethik-Kommission der Med. Fakultaet der LMU Muenchen, Prof. Dr. med. Gustav Paumgartner, Klinikum der Universitaet Grosshadern Marchioninistreet 15, Muenchen, 81377 Germany.<br><br>Chairperson- Prof. Dr. Wolfgang Eisenmenger

Investigator	Investigator no./Center no.	Description of Research Facility, Hospital/ Institution, and Address	Name of IEC/IRB Committee, Address, Committee Chair
Latta, Sabine. Dr.med<br>Warm, Mathias. Dr.med<br>(Former PI)	222522/037376	Dr. med. Sabine Latta, Klinikum der Universitaet zu Koeln, Kerpener Street 62, Koeln,<br>50924 Germany.	Ethik-Kommission der Med. Fakultaet der LMU Muenchen, Prof. Dr. med. Gustav Paumgartner, Klinikum der Universitaet Grosshadern Marchioninistreet 15, Muenchen, 81377 Germany.<br><br>Chairperson- Prof. Dr. Wolfgang Eisenmenger
Leitsmann, Horst. Dr.sc.med	091035/030411	Prof.Dr.sc.med. Horst Leitsmann, Praxis Dres. Leitsrnann und Lenk, Goethestreet 21, Zwickau, 08060 Germany.	Ethik-Kommission der Med. Fakultaet der LMU Muenchen, Prof. Dr. med. Gustav Paumgartner, Klinikum der Universitaet Grosshadern Marchioninistreet 15, Muenchen, 81377 Germany.<br><br>Chairperson- Prof. Dr. Wolfgang Eisenmenger
Luhn, Birgit. Dr.med<br>Lipp, Rainer. Dr.med (Former	063764/030412	OncoResearch Lerchenfeld UG, Lerchenfeld 14, Hamburg, 22081 Germany.	Ethik-Kommission der Med. Fakultaet der LMU Muenchen, Prof. Dr. med. Gustav

Investigator	Investigator no./Center no.	Description of Research Facility, Hospital/ Institution, and Address	Name of IEC/IRB Committee, Address, Committee Chair
PI)			Paumgartner, Klinikum der Universitaet Grosshadern Marchioninistreet 15, Muenchen, 81377 Germany.<br><br>Chairperson- Prof. Dr. Wolfgang Eisenmenger
Maasberg, Michael. Dr.med	095232/030414	Dr. med. Michael Maasberg, Praxis Dr. med. Michael Maasberg, Siegfriedstr. 20, Mayen, 56727 Germany.	Ethik-Kommission der Med. Fakultaet der LMU Muenchen, Prof. Dr. med. Gustav Paumgartner, Klinikum der Universitaet Grosshadern Marchioninistreet 15, Muenchen, 81377 Germany.<br><br>Chairperson- Prof. Dr. Wolfgang Eisenmenger
Madjar, Helmut. Dr.med	095235/030415	Prof. Dr. med. Helmut Madjar, Stiftung Deutsche Klinik fuer Diagnostik GmbH, Gynaekologie, Aukarnmallee 33, Wiesbaden, 65191 Germany.	Ethik-Kommission der Med. Fakultaet der LMU Muenchen, Prof. Dr. med. Gustav Paumgartner, Klinikum der Universitaet Grosshadern Marchioninistreet 15,

Investigator	Investigator no./Center no.	Description of Research Facility, Hospital/ Institution, and Address	Name of IEC/IRB Committee, Address, Committee Chair
			Muenchen, 81377 Germany.<br><br>Chairperson- Prof. Dr. Wolfgang Eisenmenger
Marschner, Norbert. Dr.med	091041/037348	Dr. med. Norbert Marschner, Onkologische, Gemeinschaftspraxis, Breisacher Street 117, Freiburg, 79106 Germany.	Ethik-Kommission der Med. Fakultaet der LMU Muenchen, Prof. Dr. med. Gustav Paumgartner, Klinikum der Universitaet Grosshadern Marchioninistreet 15, Muenchen, 81377 Germany.<br><br>Chairperson- Prof. Dr. Wolfgang Eisenmenger
Mau, Christine. Dr.med<br>Wunschel, Aysen. Dr.med (Former PI)<br>Keil, Elke. Dr.med (Former PI)	126433/030408	Dr. med. Christine Mau, Helios Klinikum Berlin Buch, Brustzentrum, Schwan becker Chausee 50, Berlin, 13125 Germany.	Ethik-Kommission der Med. Fakultaet der LMU Muenchen, Prof. Dr. med. Gustav Paumgartner, Klinikum der Universitaet Grosshadern Marchioninistreet 15, Muenchen, 81377 Germany.

Investigator	Investigator no./Center no.	Description of Research Facility, Hospital/ Institution, and Address	Name of IEC/IRB Committee, Address, Committee Chair
			Chairperson- Prof. Dr. Wolfgang Eisenmenger
Moebus, Volker. Dr.med*	040470/031800	Prof. Dr. med. Volker Moebus, Staedtische Kliniken Frankfurt am Main – Hoechst, Gotenstr. 6-8, Frankfurt a.M., 65929 Germany.	Ethik-Kommission der Med. Fakultaet der LMU Muenchen, Prof. Dr. med. Gustav Paumgartner, Klinikum der Universitaet Grosshadern Marchioninistreet 15, Muenchen, 81377 Germany.<br><br>Chairperson- Prof. Dr. Wolfgang Eisenmenger
Morack, Doerthe. Dr.med*	091049/031801	Dr. med. Doerthe Momck, Praxis Dr. med. Doerthe Morack, Blankenburger Street 47, 1 Berlin, 3156 Germany.	Ethik-Kommission der Med. Fakultaet der LMU Muenchen, Prof. Dr. med. Gustav Paumgartner, Klinikum der Universitaet Grosshadern Marchioninistreet 15, Muenchen, 81377 Germany.<br><br>Chairperson- Prof. Dr. Wolfgang Eisenmenger

Investigator	Investigator no./Center no.	Description of Research Facility, Hospital/ Institution, and Address	Name of IEC/IRB Committee, Address, Committee Chair
Mueller, Volkmar. Dr.med<br>Lisboa, Bjoern. Dr.med (Former PI)	091170/030413	Universitaetsklinikum Harnburg-Eppendorf, Brustzentrum, Martinistr.52, 2 Hamburg, 0246 Germany.	Ethik-Kommission der Med. Fakultaet der LMU Muenchen, Prof. Dr. med. Gustav Paumgartner, Klinikum der Universitaet Grosshadern Marchioninistreet 15, Muenchen, 81377 Germany.<br><br>Chairperson- Prof. Dr. Wolfgang Eisenmenger
Mueller-Hagen, Sigrun. Dr.med	054139/033514	Dr. med. Sigrun Mueller-Hagen, Haematologisch-oncologischer Schwerpunkt, Pinneberger Street 25, Hamburg, 22457 Germany.	Ethik-Kommission der Med. Fakultaet der LMU Muenchen, Prof. Dr. med. Gustav Paumgartner, Klinikum der Universitaet Grosshadern Marchioninistreet 15, Muenchen, 81377 Germany.<br><br>Chairperson- Prof. Dr. Wolfgang Eisenmenger
Nusch, Arnd. Dr.med	062508/030418	Dr. med. Arnd Nusch, Praxis Dr. rned. Arnd Nusch, Friedrichstr, 311, Velbert, 42551	Ethik-Kommission der Med. Fakultaet der LMU Muenchen, Prof. Dr. med. Gustav

Investigator	Investigator no./Center no.	Description of Research Facility, Hospital/ Institution, and Address	Name of IEC/IRB Committee, Address, Committee Chair
		Germany.	Paumgartner, Klinikum der Universitaet Grosshadern Marchioninistreet 15, Muenchen, 81377 Germany.<br><br>Chairperson- Prof. Dr. Wolfgang Eisenmenger
Overkamp, Friedrich. Dr.med	116542/039515	Dr. med. Friedrich Overkamp, Oncologianova GmbH, Springstrasse 24, Recklinghausen, 45657 Germany.	Ethik-Kommission der Med. Fakultaet der LMU Muenchen, Prof. Dr. med. Gustav Paumgartner, Klinikum der Universitaet Grosshadern Marchioninistreet 15, Muenchen, 81377 Germany.<br><br>Chairperson- Prof. Dr. Wolfgang Eisenmenger
Papke, Jens. Dr.med	090996/030419	Dr. med. Jens Papke, Praxis Dr. med. Jens Papke Rosa-Luxemburg-Street 6, Neustadt, 01844 Germany.	Ethik-Kommission der Med. Fakultaet der LMU Muenchen, Prof. Dr. med. Gustav Paumgartner, Klinikum der Universitaet Grosshadern Marchioninistreet 15,

Investigator	Investigator no./Center no.	Description of Research Facility, Hospital/ Institution, and Address	Name of IEC/IRB Committee, Address, Committee Chair
			Muenchen, 81377 Germany.<br><br>Chairperson- Prof. Dr. Wolfgang Eisenmenger
Park-Simon, Tjoung-Won. Dr.med<br>Kuehnle, Henning. Dr.med (Former PI)	154925/031971	Prof. Dr. rned. Tjoung-Won Park-Simon, Medizinische Hochschule Hannover, Allg. Gynaekologie und Geburtshilfe, Carl-Neuberg-Street 1, Hannover, 30625 Germany.	Ethik-Kommission der Med. Fakultaet der LMU Muenchen, Prof. Dr. med. Gustav Paumgartner, Klinikum der Universitaet Grosshadern Marchioninistreet 15, Muenchen, 81377 Germany.<br><br>Chairperson- Prof. Dr. Wolfgang Eisenmenger
Peters, Martin. Dr.med<br>Meinen, Klaus. Dr.med (Former PI)	098245/031798	Dr. med. Martin Peters, Saint. Josels Krankenhaus Hilden GmbH, Senologie Walder Street 34-38, Hilden, 40724 Germany.	Ethik-Kommission der Med. Fakultaet der LMU Muenchen, Prof. Dr. med. Gustav Paumgartner, Klinikum der Universitaet Grosshadern Marchioninistreet 15, Muenchen, 81377 Germany.

Investigator	Investigator no./Center no.	Description of Research Facility, Hospital/ Institution, and Address	Name of IEC/IRB Committee, Address, Committee Chair
			Chairperson- Prof. Dr. Wolfgang Eisenmenger
Pihusch, Rudolf. PD, Dr.med*	116546/039516	PD Dr. med. Rudolf Pihusch, Tagesklinik, Rosenheim - Tagesklinik Bad Trissl, Onkologische Schwerpunktpraxis, Rathausstreet 14, 83022 Rosenheim, 83022 Germany.	Ethik-Kommission der Med. Fakultaet der LMU Muenchen, Prof. Dr. med. Gustav Paumgartner, Klinikum der Universitaet Grosshadern Marchioninistreet 15, Muenchen, 81377 Germany.<br><br>Chairperson- Prof. Dr. Wolfgang Eisenmenger
Reles, Angela. PD,Dr.med	052244/039518	PD Dr. med. Angela Reles, Arztpraxis fuer Frauenheilkunde und Geburtshilfe, Ruedesheimer Platz 5, Berlin, 14197 Germany.	Ethik-Kommission der Med. Fakultaet der LMU Muenchen, Prof. Dr. med. Gustav Paumgartner, Klinikum der Universitaet Grosshadern Marchioninistreet 15, Muenchen, 81377 Germany.<br><br>Chairperson- Prof. Dr. Wolfgang Eisenmenger

Investigator	Investigator no./Center no.	Description of Research Facility, Hospital/ Institution, and Address	Name of IEC/IRB Committee, Address, Committee Chair
Rezai, Mahdi. Dr.med*	090999/031803	Dr. med. Mahdi Rezai, Brustzentrum Duesseldorf Luisenkrankenhaus, Degerstrasse 8, Duesseldorf, 40235 Germany.	Ethik-Kommission der Med. Fakultaet der LMU Muenchen, Prof. Dr. med. Gustav Paumgartner, Klinikum der Universitaet Grosshadern Marchioninistreet 15, Muenchen, 81377 Germany.<br><br>Chairperson- Prof. Dr. Wolfgang Eisenmenger
Ruffert, Klaus. PD, Dr.med	091001/030420	PD Dr. med. Klaus Ruffert, Early Phase Solutions GmbH (EPS), Goethe Galerie 3b, Jena, 07743 Germany.	Ethik-Kommission der Med. Fakultaet der LMU Muenchen, Prof. Dr. med. Gustav Paumgartner, Klinikum der Universitaet Grosshadern Marchioninistreet 15, Muenchen, 81377 Germany.<br><br>Chairperson- Prof. Dr. Wolfgang Eisenmenger
Ruhmland, Birgit. Dr.med	116640/039519	Dr. med. Birgit Ruhmland, Frauenheilkunde und Geburtshilfe, Eisterwerdaer Platz 1, 12683	Ethik-Kommission der Med. Fakultaet der LMU Muenchen, Prof. Dr. med. Gustav

Investigator	Investigator no./Center no.	Description of Research Facility, Hospital/ Institution, and Address	Name of IEC/IRB Committee, Address, Committee Chair
		Berlin, Germany.	Paumgartner, Klinikum der Universitaet Grosshadern Marchioninistreet 15, Muenchen, 81377 Germany.<br><br>Chairperson- Prof. Dr. Wolfgang Eisenmenger
Runnebaum, Ingo. Dr.med	091003/031804	Prof. Dr. med Ingo Runnebaum, Klinikum der Friendrich-Schiller-Universitaet Jena, Interdisziplinaeres Brustzentrum Jena, Bachstrasse 18, Jena, 07743 Germany.	Ethik-Kommission der Med. Fakultaet der LMU Muenchen, Prof. Dr. med. Gustav Paumgartner, Klinikum der Universitaet Grosshadern Marchioninistreet 15, Muenchen, 81377 Germany.<br><br>Chairperson- Prof. Dr. Wolfgang Eisenmenger
Sallmann, Alexandra<br>Jochim-Maier, Rosa. Dr.med (Former PI)<br>Hauschild, Maik. Dr.med	184790/030404	Frauenklinik Rheinfelden, Brustzentrum, Therese-Herzog-Weg 2, Rheinfelden/BD, 79618 Germany.	Ethik-Kommission der Med. Fakultaet der LMU Muenchen, Prof. Dr. med. Gustav Paumgartner, Klinikum der Universitaet Grosshadern Marchioninistreet 15,

Investigator	Investigator no./Center no.	Description of Research Facility, Hospital/ Institution, and Address	Name of IEC/IRB Committee, Address, Committee Chair
(Former PI)			Muenchen, 81377 Germany.<br><br>Chairperson- Prof. Dr. Wolfgang Eisenmenger
Schaefers, Michael. Dr.med	119143/040228	Dr. med. Michael Schaefers, Mercatorstreet 58, Duisburg, 47051 Germany.	Ethik-Kommission der Med. Fakultaet der LMU Muenchen, Prof. Dr. med. Gustav Paumgartner, Klinikum der Universitaet Grosshadern Marchioninistreet 15, Muenchen, 81377 Germany.<br><br>Chairperson- Prof. Dr. Wolfgang Eisenmenger
Schleicher, Peter. Dr.med	114503/038547	Dr. med. Peter Schleicher, Praxis Dres. Bernd und Peter Schleicher, Breite Street 12, Schwandorf, 92421 Germany.	Ethik-Kommission der Med. Fakultaet der LMU Muenchen, Prof. Dr. med. Gustav Paumgartner, Klinikum der Universitaet Grosshadern Marchioninistreet 15, Muenchen, 81377 Germany.

Investigator	Investigator no./Center no.	Description of Research Facility, Hospital/ Institution, and Address	Name of IEC/IRB Committee, Address, Committee Chair
			Chairperson- Prof. Dr. Wolfgang Eisenmenger
Schmidt, Marcus. Dr. med	087247/030422	Dr. med. Marcus Schmidt, Klinikum der Johannes-Gutenberg Universitaet Mainz, Brustzentrum, Langenbeckstreet 1, Mainz, 55101 Germany.	Ethik-Kommission der Med. Fakultaet der LMU Muenchen, Prof. Dr. med. Gustav Paumgartner, Klinikum der Universitaet Grosshadern Marchioninistreet 15, Muenchen, 81377 Germany.<br><br>Chairperson- Prof. Dr. Wolfgang Eisenmenger
Schmidt, Peter. Dr. med	056821/030423	Dr. med. Peter Schmidt, Praxis Dr. med. Peter Schmidt, Onkologische Schwerpunktpraxis, Hebbelstreet 2, Neunkirchen, 66538 Germany.	Ethik-Kommission der Med. Fakultaet der LMU Muenchen, Prof. Dr. med. Gustav Paumgartner, Klinikum der Universitaet Grosshadern Marchioninistreet 15, Muenchen, 81377 Germany.<br><br>Chairperson- Prof. Dr. Wolfgang Eisenmenger

Investigator	Investigator no./Center no.	Description of Research Facility, Hospital/ Institution, and Address	Name of IEC/IRB Committee, Address, Committee Chair
Schmitz, Stephan. PD Dr. med	092745/038203	PD Dr. med. Stephan Schmitz, Praxis fuer Haematologie und Onkologie, Sachsenring 69, Koeln, 50677 Germany.	Ethik-Kommission der Med. Fakultaet der LMU Muenchen, Prof. Dr. med. Gustav Paumgartner, Klinikum der Universitaet Grosshadern Marchioninistreet 15, Muenchen, 81377 Germany.<br><br>Chairperson- Prof. Dr. Wolfgang Eisenmenger
Schnappauf, Benjamin<br>von Minckwitz, Gunter (Former PI)<br>Schwedler, Kathrin. Dr.med (Former PI)	108355/031816	Klinikum der Johann-Wolfgang Goethe-Universitaet, Zentrum fuer Frauenhellkunde und Geburtshilfa, Theodor-Stern-Kai 7, , Frankfurt a. M, 60590 Germany.	Ethik-Kommission der Med. Fakultaet der LMU Muenchen, Prof. Dr. med. Gustav Paumgartner, Klinikum der Universitaet Grosshadern Marchioninistreet 15, Muenchen, 81377 Germany.<br><br>Chairperson- Prof. Dr. Wolfgang Eisenmenger
Schneider, Achim. Dr. med.	058871/031807	Prof. Dr. med. Achim Schneider, Charite Universitaetsmedizin Berlin, Interdisziplinaeres	Ethik-Kommission der Med. Fakultaet der LMU Muenchen, Prof. Dr. med. Gustav

Investigator	Investigator no./Center no.	Description of Research Facility, Hospital/ Institution, and Address	Name of IEC/IRB Committee, Address, Committee Chair
		Brustzentrum. Campus Mitte, Charite Platz 1, Berlin, 10117 Germany.	Paumgartner, Klinikum der Universitaet Grosshadern Marchioninistreet 15, Muenchen, 81377 Germany.<br><br>Chairperson- Prof. Dr. Wolfgang Eisenmenger
Schroeder, Jan. Dr. med.	091019/031808	Dr. med. Jan Schroeder, Gemeinschaftspraxis Dres. Schroeder und Sieg, Kettwigerstr. 62, 45473 Muelheim a.d.R, Germany.	Ethik-Kommission der Med. Fakultaet der LMU Muenchen, Prof. Dr. med. Gustav Paumgartner, Klinikum der Universitaet Grosshadern Marchioninistreet 15, Muenchen, 81377 Germany.<br><br>Chairperson- Prof. Dr. Wolfgang Eisenmenger
Schroeder, Willibald. Prof. Dr. Med*	091023/030424	Herr Prof. Dr. med. Willibald Schroeder, Klinikum Bremen-Mitte gGmbH, Brustzentrum Klinikum Bremen-Mitte (ZKH), Street.-Juergen-Street. 1, Bremen, 28205 Germany.	Ethik-Kommission der Med. Fakultaet der LMU Muenchen, Prof. Dr. med. Gustav Paumgartner, Klinikum der Universitaet Grosshadern Marchioninistreet 15,

Investigator	Investigator no./Center no.	Description of Research Facility, Hospital/ Institution, and Address	Name of IEC/IRB Committee, Address, Committee Chair
			Muenchen, 81377 Germany.<br><br>Chairperson- Prof. Dr. Wolfgang Eisenmenger
Schulz, Volker. Dr.med	056814/030425	Brustzentrum Kiel Mitte, Pruener Gang 7, Klel, 24103 Germany.	Ethik-Kommission der Med. Fakultaet der LMU Muenchen, Prof. Dr. med. Gustav Paumgartner, Klinikum der Universitaet Grosshadern Marchioninistreet 15, Muenchen, 81377 Germany.<br><br>Chairperson- Prof. Dr. Wolfgang Eisenmenger
Schulze, Mathias. Dr. med	091031/030426	Dr. med. Mathias Schulze, Praxis Dr. med. Mathias Schulze, Goerlitzer Street 10a, Zittau, 02763 Germany.	Ethik-Kommission der Med. Fakultaet der LMU Muenchen, Prof. Dr. med. Gustav Paumgartner, Klinikum der Universitaet Grosshadern Marchioninistreet 15, Muenchen, 81377 Germany.

Investigator	Investigator no./Center no.	Description of Research Facility, Hospital/ Institution, and Address	Name of IEC/IRB Committee, Address, Committee Chair
			Chairperson- Prof. Dr. Wolfgang Eisenmenger
Selbach, Johannes. Dr.med	091033/031809	Dr. med. Johannes Selbach, Praxis Dr. med. Johannes Selbach, Schreckerstr. 16, Duisburg, 47166 Germany.	Ethik-Kommission der Med. Fakultaet der LMU Muenchen, Prof. Dr. med. Gustav Paumgartner, Klinikum der Universitaet Grosshadern Marchioninistreet 15, Muenchen, 81377 Germany.<br><br>Chairperson- Prof. Dr. Wolfgang Eisenmenger
Simon, Wolfgang. Dr. med	091036/031810	Prof. Dr. med. Wolfgang Simon, Robert-Bosch-Krankenhaus GmbH, Brustzentrum, Auerbachstreet 110, Stuttgart, 70376 Germany,	Ethik-Kommission der Med. Fakultaet der LMU Muenchen, Prof. Dr. med. Gustav Paumgartner, Klinikum der Universitaet Grosshadern Marchioninistreet 15, Muenchen, 81377 Germany.<br><br>Chairperson- Prof. Dr. Wolfgang Eisenmenger

Investigator	Investigator no./Center no.	Description of Research Facility, Hospital/ Institution, and Address	Name of IEC/IRB Committee, Address, Committee Chair
Solomayer, Erich. Dr.<br>Schmidt, Werner. Dr (Former PI)	179164/031805	Prof, Dr. med, Franz Erich Solomayar, Universitatsklinik des Saarlandes, Brustzentrum Homburg/Saar Kirrberger Strasse, Haus 9, Homburg, Saar, 66421 Germany.	Ethik-Kommission der Med. Fakultaet der LMU Muenchen, Prof. Dr. med. Gustav Paumgartner, Klinikum der Universitaet Grosshadern Marchioninistreet 15, Muenchen, 81377 Germany.<br><br>Chairperson- Prof. Dr. Wolfgang Eisenmenger
Stefek, Andrea. Dr<br>Henschen, Stephan. Dr.med (Former PI)	233396/030407	Johanniter KH Genthin Stendal gGmbH, Brustzentrum Altmark, Bahnhofstreet 24-26, Stendal, 39576 Germany.	Ethik-Kommission der Med. Fakultaet der LMU Muenchen, Prof. Dr. med. Gustav Paumgartner, Klinikum der Universitaet Grosshadern Marchioninistreet 15, Muenchen, 81377 Germany.<br><br>Chairperson- Prof. Dr. Wolfgang Eisenmenger
Terhaag, Juergen. Dr.med	091046/031812	Dr. med. Juergen Terhaag, Kreiskrankenhaeuser Rottal-Inn gGmbH,	Ethik-Kommission der Med. Fakultaet der LMU Muenchen, Prof. Dr. med. Gustav

Investigator	Investigator no./Center no.	Description of Research Facility, Hospital/ Institution, and Address	Name of IEC/IRB Committee, Address, Committee Chair
		Simonsoeder Allee 20, Eggenfelden, 84307 Germany.	Paumgartner, Klinikum der Universitaet Grosshadern Marchioninistreet 15, Muenchen, 81377 Germany.<br><br>Chairperson- Prof. Dr. Wolfgang Eisenmenger
Tessen, Hans Werner. Dr. med	064531/030427	Dr. med. Hans Werner Tessen, Praxis Dr. med. Hans Werner Tessen, Koesliner Street 10b, Goslar, 38642 Germany.	Ethik-Kommission der Med. Fakultaet der LMU Muenchen, Prof. Dr. med. Gustav Paumgartner, Klinikum der Universitaet Grosshadern Marchioninistreet 15, Muenchen, 81377 Germany.<br><br>Chairperson- Prof. Dr. Wolfgang Eisenmenger
Thomalla, Joerg. Dr.med	102315/033634	Dr. med. Joerg Thomalla, Institut fur Versorgungsforschung in der Onkologie GbR, Neversstr. 5, Koblenz, 56068 Germany.	Ethik-Kommission der Med. Fakultaet der LMU Muenchen, Prof. Dr. med. Gustav Paumgartner, Klinikum der Universitaet Grosshadern Marchioninistreet 15,

Investigator	Investigator no./Center no.	Description of Research Facility, Hospital/ Institution, and Address	Name of IEC/IRB Committee, Address, Committee Chair
			Muenchen, 81377 Germany.<br><br>Chairperson- Prof. Dr. Wolfgang Eisenmenger
Thomssen, Christoph. Dr. med	040473/030428	Herr Prof. Dr. med. Christoph Thomssen, Martin-Luther-Universitaet Halle-Wittenberg, Klinik und Poliklinik fuer Gynaekologie, Ernst-Grube-Street 40, Halle/Saale, 06097 Germany.	Ethik-Kommission der Med. Fakultaet der LMU Muenchen, Prof. Dr. med. Gustav Paumgartner, Klinikum der Universitaet Grosshadern Marchioninistreet 15, Muenchen, 81377 Germany.<br><br>Chairperson- Prof. Dr. Wolfgang Eisenmenger
Tulusan, Augustinus Dr. Med*	037534/031813	Prof. Dr. med. Augustinus Tulusan, Klinikum Bayreuth GmbH, Brustzentrum, Preuschwitzerstreet 101, Bayreuth, 95445 Germany.	Ethik-Kommission der Med. Fakultaet der LMU Muenchen, Prof. Dr. med. Gustav Paumgartner, Klinikum der Universitaet Grosshadern Marchioninistreet 15, Muenchen, 81377 Germany.

Investigator	Investigator no./Center no.	Description of Research Facility, Hospital/ Institution, and Address	Name of IEC/IRB Committee, Address, Committee Chair
			Chairperson- Prof. Dr. Wolfgang Eisenmenger
Uleer, Christoph. Dr.med	056819/031815	Dr. med. Christoph Uleer, Praxis Dr. med. Christoph Uleer, Bahnhofplatz 5, Hildesheim, 31134 Germany.	Ethik-Kommission der Med. Fakultaet der LMU Muenchen, Prof. Dr. med. Gustav Paumgartner, Klinikum der Universitaet Grosshadern Marchioninistreet 15, Muenchen, 81377 Germany.<br><br>Chairperson- Prof. Dr. Wolfgang Eisenmenger
Voigtmann, Rudolf. Dr.med	112550/037384	Prof. Dr. med. Rudolf Voigtmann, Technologiezentrurn der Ruhr-Universitaet, Universitaetsstreet 142, Bochum, 44799 Germany.	Ethik-Kommission der Med. Fakultaet der LMU Muenchen, Prof. Dr. med. Gustav Paumgartner, Klinikum der Universitaet Grosshadern Marchioninistreet 15, Muenchen, 81377 Germany.<br><br>Chairperson- Prof. Dr. Wolfgang Eisenmenger

Investigator	Investigator no./Center no.	Description of Research Facility, Hospital/ Institution, and Address	Name of IEC/IRB Committee, Address, Committee Chair
von Abel, Ekkehard. Dr.med<br>Schlicht, Erik (Former PI)	096744/030421	Dr. med. Ekkehard von Abel, Kiinikum Schwaebisch Gmuend Frauenkiinik, Brustzentrum Schwaebisch Gmuend, Wetzgauer Strasse 85, Mutlangen, 73557 Germany.	Ethik-Kommission der Med. Fakultaet der LMU Muenchen, Prof. Dr. med. Gustav Paumgartner, Klinikum der Universitaet Grosshadern Marchioninistreet 15, Muenchen, 81377 Germany.<br><br>Chairperson- Prof. Dr. Wolfgang Eisenmenger
von Gruenhagen, Ulrich. Dr.med	091050/030430	Dr. med. Ulrich von Gruenhagen, Praxis Dr. rned. Ulrich von Gruenhagen, Bahnhofstr. 63, Cottbus, 03046 Germany.	Ethik-Kommission der Med. Fakultaet der LMU Muenchen, Prof. Dr. med. Gustav Paumgartner, Klinikum der Universitaet Grosshadern Marchioninistreet 15, Muenchen, 81377 Germany.<br><br>Chairperson- Prof. Dr. Wolfgang Eisenmenger
Walther, Jochem. Dr.med	067705/031817	Dr.med. Jochem Walther, Haematologisch-Onkologische Schwerpunktpraxis mit	Ethik-Kommission der Med. Fakultaet der LMU Muenchen, Prof. Dr. med. Gustav

Investigator	Investigator no./Center no.	Description of Research Facility, Hospital/ Institution, and Address	Name of IEC/IRB Committee, Address, Committee Chair
		Tagesklinik, Sonnenstreet 11, Muenchen, 80331 Germany.	Paumgartner, Klinikum der Universitaet Grosshadern Marchioninistreet 15, Muenchen, 81377 Germany.<br><br>Chairperson- Prof. Dr. Wolfgang Eisenmenger
Bregulla, Markus<br>Weiss, Johann. Dr.med<br>(Former PI)	192953/030431	Dr. Markus Bregulla, Praxis Dr. med. Bregulla, Friedrich Ebert Street 12 a, Weiden, 92637 Germany.	Ethik-Kommission der Med. Fakultaet der LMU Muenchen, Prof. Dr. med. Gustav Paumgartner, Klinikum der Universitaet Grosshadern Marchioninistreet 15, Muenchen, 81377 Germany.<br><br>Chairperson- Prof. Dr. Wolfgang Eisenmenger
Welslau, Manfred. Dr.med	091058/030432	Dr. med. Manfred Welslau<br>Praxis Dr. med. Manfred Welslau<br>Elisenstr. 2 6, 63739 Aschaffenburg, Germany.	Ethik-Kommission der Med. Fakultaet der LMU Muenchen, Prof. Dr. med. Gustav Paumgartner, Klinikum der Universitaet Grosshadern Marchioninistreet 15,

Investigator	Investigator no./Center no.	Description of Research Facility, Hospital/ Institution, and Address	Name of IEC/IRB Committee, Address, Committee Chair
			Muenchen, 81377 Germany.<br><br>Chairperson- Prof. Dr. Wolfgang Eisenmenger
Wetzel, Kuno. Dr.med	116544/039520	Dr. med. Kuno Wetzel, Praxis Dr. med. Kuno Wetzel, Helsunger Street 7, Blankenburg, 38889 Germany.	Ethik-Kommission der Med. Fakultaet der LMU Muenchen, Prof. Dr. med. Gustav Paumgartner, Klinikum der Universitaet Grosshadern Marchioninistreet 15, Muenchen, 81377 Germany.<br><br>Chairperson- Prof. Dr. Wolfgang Eisenmenger
Wiest, Wolfgang. Dr.med	091059/030433	Katholisches Klinikum Mainz, Brustzentrum, An der Goldgrube 11, Mainz, 55131 Germany.	Ethik-Kommission der Med. Fakultaet der LMU Muenchen, Prof. Dr. med. Gustav Paumgartner, Klinikum der Universitaet Grosshadern Marchioninistreet 15, Muenchen, 81377 Germany.

Investigator	Investigator no./Center no.	Description of Research Facility, Hospital/ Institution, and Address	Name of IEC/IRB Committee, Address, Committee Chair
			Chairperson- Prof. Dr. Wolfgang Eisenmenger
Wilke, Jochen. Dr.med	091060/030434	Dr. med. Jochen Wilke, Praxis Dr. med. Jochen Wilke, Jakob Henle Street 1, Fuerth, 90766 Germany	Ethik-Kommission der Med. Fakultaet der LMU Muenchen, Prof. Dr. med. Gustav Paumgartner, Klinikum der Universitaet Grosshadern Marchioninistreet 15, Muenchen, 81377 Germany.<br><br>Chairperson- Prof. Dr. Wolfgang Eisenmenger
**Greece**			
Georgoulias, Vassilis. MD	002886/031401	Medical Oncology Department, University Hospital of Heraklion, Stavrakion and Voutes street Heraklion, 71201, Greece.	National Drug Organisation (NGO) 284, Mesogion Avenue 15562, Holargos, Greece. Chairperson- Ioannis Tountas<br><br>National Ethics Committee (NEC)<br>284, Mesogion Avenue, 15562, Holargos, Greece.

Investigator	Investigator no./Center no.	Description of Research Facility, Hospital/ Institution, and Address	Name of IEC/IRB Committee, Address, Committee Chair
			Chairperson- Konstantinos Dimopoulos
Kontzoglou, Konstantinos. MD	103794/034299	2nd Surgery Clinic, General Hospital of Athens "Laiko", 17 Agiou Thoma street, Athens, 11527, Greece.	National Drug Organisation (NGO) 284, Mesogion Avenue 15562, Holargos, Greece. Chairperson- Ioannis Tountas<br><br>National Ethics Committee (NEC)<br>284, Mesogion Avenue 15562, Holargos, Greece.<br>Chairperson- Konstantinos Dimopoulos
Mylonakis, Nikolaos. MD	084803/031404	"METAXA' General Cancer Hospital, 2nd Internal Medicine Clinic, 51 Botasi Street, Athens18537, Greece.	National Drug Organisation (NGO) 284, Mesogion Avenue 15562, Holargos, Greece. Chairperson- Ioannis Tountas<br><br>National Ethics Committee (NEC)<br>284, Mesogion Avenue 15562, Holargos, Greece.<br>Chairperson- Konstantinos Dimopoulos

Investigator	Investigator no./Center no.	Description of Research Facility, Hospital/ Institution, and Address	Name of IEC/IRB Committee, Address, Committee Chair
Syrigos, Konstantinos. MD, PhD	109576/036015	General Hospital of Chest Diseases of Athens "Sotiria" 3rd University Pathology Clinic-Oncology Unit, 152 Mesogeion Avenue, 11527 Athens, Greece.	National Drug Organisation (NGO) 284, Mesogion Avenue 15562, Holargos, Greece. Chairperson- Ioannis Tountas<br><br>National Ethics Committee (NEC) 284, Mesogion Avenue 15562, Holargos, Greece. Chairperson- Konstantinos Dimopoulos.
Varthalitis, Ioannis. MD	105609/035140	Oncology Clinic General Hospital of Chania, Mournies Chania 733 00, Greece.	National Drug Organisation (NGO) 284, Mesogion Avenue 15562, Holargos, Greece. Chairperson- Ioannis Tountas.<br><br>National Ethics Committee (NEC) 284, Mesogion Avenue 15562, Holargos, Greece. Chairperson- Konstantinos Dimopoulos.
Zografos, Georgios. MD	104326/035141	General Hospital of Athens "Ippokratio" A' Propaedeutic Surgery University Clinic 114 Vas. Sofias Avenue, Athens 11527, Greece.	National Drug Organisation (NGO) 284, Mesogion Avenue 15562, Holargos, Greece. Chairperson- Ioannis Tountas.

Investigator	Investigator no./Center no.	Description of Research Facility, Hospital/ Institution, and Address	Name of IEC/IRB Committee, Address, Committee Chair
			National Ethics Committee (NEC)<br>284, Mesogion Avenue 15562, Holargos, Greece.<br>Chairperson- Konstantinos Dimopoulos.
**Hong Kong**			
Chow, Louis. MBBS, MS	041380/029948	UNIMED Medical Institute Comprehensive Centre for Breast Disease, 10/F Luk Kwok Centre, 72 Gloucester Road, Wanchai, Hong Kong.	UNIMED Medical Institute Institutional Review Board, Department of Clinical Oncology, PB-115, Queen Mary Hospital, 102 Pokfulam Road, Hong Kong<br><br>Chairperson: Dr. Ashely Cheng
Liang, Raymond. MD<br>Epstein Richard. MD (Former PI)	061340/029946	Department of Medicine, Queen Mary Hospital, 102 Pokfulam Road, Hong Kong.	Institutional Review Board of the Univesity of Hong Kong/Hospital Authority Hong Kong West Cluster, Room 901, Administration Block, Queen Mary Hospital, 102 Pokfulam Road, Hong Kong.

Investigator	Investigator no./Center no.	Description of Research Facility, Hospital/ Institution, and Address	Name of IEC/IRB Committee, Address, Committee Chair
			Chairperson: Dr. Virginia Wong
Yeo, Winnie. MD	041381/029947	Department of Clinical Oncology, Prince of Wales Hospital, 30-32 Ngan Shing Street, Shatin, Hong Kong.	Joint CUHK-New Territories East Cluster Clinical Research Ethics Committee, Centre for Clinical Research Trials and Epidemiological Research, 5/F School of Public Health, Prince of Wales Hospital, 30-32 Ngan Shing Street, Shatin, Hong Kong.<br><br>Chairperson: Dr Benny ZeeZ
**Hungary**			
Kahan, Zsuzsanna. MD	084500/049544	Szent-Gyorgyi Albert Klinikai Kozpont, Onkoterapias Klinika, Koranyl fasor 12, H-6720 Szeged, Hungary.	Egeszsegugyi Tudomanyos Tanacs, Klinikofarmakologlai Etikai Bizottsaga, Arany J. u. 6-8, Budapest- 1051, Hungary. Chairperson- Zsuzsanna Fürst<br><br>Orszagos Gyogyszereszeti Intezet, 1051 Budapest, Zriyni u. 3, Hungary.

Investigator	Investigator no./Center no.	Description of Research Facility, Hospital/ Institution, and Address	Name of IEC/IRB Committee, Address, Committee Chair
			Chairperson- Zsuzsanna Fürst
Kammerer, Kinga. MD	057358/033197	Pest Megyei Flor Ferenc Korhaz, Onkologia Osztaly, Klstarca H-2143, Semmelweis ter 1 Hungary.	Egeszsegugyi Tudomanyos Tanacs, Klinikofarmakologlai Etikai Bizottsaga, Arany J. u. 6-8, Budapest- 1051, Hungary. Chairperson- Zsuzsanna Fürst<br><br>Orszagos Gyogyszereszeti Intezet, 1051 Budapest, Zriyni u. 3, Hungary. Chairperson- Zsuzsanna Fürst
Magyar, Tamás*. MD	101279/033200	Fovarosi Onkormanyzat Peterfy, Sandor Utcai Korhaza, Onkologiai Osztaly, Peterfy sandor u. 8-20 H-1076 Budapest, Hungary.	Egeszsegugyi Tudomanyos Tanacs, Klinikofarmakologlai Etikai Bizottsaga, Arany J. u. 6-8, Budapest- 1051, Hungary. Chairperson- Zsuzsanna Fürst<br><br>Orszagos Gyogyszereszeti Intezet, 1051 Budapest, Zriyni u. 3, Hungary. Chairperson- Zsuzsanna Fürst

Investigator	Investigator no./Center no.	Description of Research Facility, Hospital/ Institution, and Address	Name of IEC/IRB Committee, Address, Committee Chair
Pápai, Zsuzsanna. MD	101270/033202	Allami Egeszsbgugyi Kozpont, Klinikai Onkologia, 1062 Budapest, Podmaniczky u.111, Hungary	Egeszsegugyi Tudomanyos Tanacs, 1051 Budapest, Arany Janos u. 6-8. Hungary. Chairperson- Zsuzsanna Fürst
Pintér, Tamás. MD	086060/033198	Petz ALadar Megyei Oktato Korhez, Onkologial Osztaly, H-9024 Gyor, Zrlnyi u., 13. Hungary.	Egeszsegugyi Tudomanyos Tanacs, Klinikofarmakologlai Etikai Bizottsaga, Arany J. u. 6-8, Budapest- 1051, Hungary. Chairperson- Zsuzsanna Fürst<br><br>Orszagos Gyogyszereszeti Intezet, 1051 Budapest, Zriyni u. 3, Hungary. Chairperson- Zsuzsanna Fürst
Somogyiné Ezer, Eva. MD<br>Győrfy, Károly. MD (Former PI)	112332/033201	Kaposi Mor Megyei Korhaz, Onkologia Osztaly Kaposvar H- 7400, Tallian gy. U. 20-32, Hungary.	Egeszsegugyi Tudomanyos Tanacs, 1051 Budapest, Arany Janos u. 6-8. Hungary.<br><br>Chairperson- Zsuzsanna Fürst
Varga, Zsuzsanna. MD	101275/033203	Pecsi Tudomanyegyetem KK, Onkotherapias Intezet H-7624 Pecs, Edesanyak utja 17. Hungary.	Egeszsegugyi Tudomanyos Tanacs Klinikai Farmakologiai Etikai Bizottsaga H-1051, Budapest Arany Janos u. 6-8, Hungary.

Investigator	Investigator no./Center no.	Description of Research Facility, Hospital/ Institution, and Address	Name of IEC/IRB Committee, Address, Committee Chair
			Chairperson- Zsuzsanna Fürst
**India**			
Digumarti, Raghunadharao	070469/030964	Department of Medical Oncology, # 607 E Block, 6th Floor, Medical Oncology Dept., Nizam's Institute of Medical Siences, Panjagutla, Hyderabad - 500 082, Andhra Pradesh, India.	Institutional Ethics Committee, Nizam's Institute of Medical Siences, Panjagutla, Hyderabad - 500 082, Andhra Pradesh, India.<br><br>Chairperson: Dr. K. Ramaswamy
Jain, Minish	096956/048057	Grand Medical Foundation's Ruby Hall Clinic, new Cancer Building, Third Floor, 40, Sassoon Road, Pune -411001, Maharashtra, India.	Poona Medical Foundation's Ruby Hall Clinic, 40, Sassoon Road, Pune -411001, Maharashtra, India.<br><br>Chairperson: Mr. B. P. Shaligram
Koppiker, Chaitanyanand	127783/045124	Jehangir Clinical Development Centre Pvt ltd, Jehangir Hospital Premises, 32 Sasson Road, Puna 411001, Maharashtra, India.	Jehangir Clinical Developmental Centre (JCDC), Jehangir Hospital, 32 Sasson Road, Pune 411001, Maharashtra, India.<br><br>Chairperson: Dr. Ravindra Ghool

Investigator	Investigator no./Center no.	Description of Research Facility, Hospital/ Institution, and Address	Name of IEC/IRB Committee, Address, Committee Chair
Maru, Anish	267058/041919	SEAROC Cancer Centre, S. K. Soni Hospital Sector 5, Vidyadhar Nagar, Sikar Road, Jaipur- 302013, Rajasthan, India.	SEAROC Ethics Committee, SEAROC Cancer Centre., S. K. Soni Hospital, Sector 5, Vidyadhar Nagar, Sikar Road, Jaipur- 302013, Rajasthan, India.<br><br>Chairperson: Mr. P. C. Jain
Nair, Reena	103833/034251	Tata Memorial Centre, Department of Medical Oncology, Dr. Ernest Borges Road, Mumbai, 400012 India.	Institutional Reveiw Board, Tata Memorial Hospital, 3rd Floor, CRS, Main BUilding, Dr. Ernest Borges Road,Parel, Mumbai - 400012, Maharashtra, India.<br><br>Chairperson: Dr. Madhuri Gore and Dr. Urmila Thatte (HEC-1 and HEC-2)
Raina, Vinod	086107/034253	Dr. B R. A Institute Rotary Cancer Hospital, All India Institute of Medical Sciences, Ansari Nagar<br>New Delhl- 110029, India.	Standing Ethics Committee, All Indio Institute of Medical Sciences (AIIMS), Ansari Nagar, New Delhi 110029, India.<br><br>Chairperson: Prof J. P. Wali

Investigator	Investigator no./Center no.	Description of Research Facility, Hospital/ Institution, and Address	Name of IEC/IRB Committee, Address, Committee Chair
Raja, Thirumalairaj	086740/033875	Apollo Speciality Hospital, Padma Complex, 320, Mount Road, Chennai - 600 006, Tamil Nadu, India.	The Ethics Committee, Apollo Hospitals Enterprise Limited, 21, Greams lane, Off Greams Road, Chennai- 600 006, Tamil Nadu, India.<br><br>Chairperson: Mr. S. Narayanan
**Israel**			
Barak (Berestechko), Frida. MD	002430/032464	Oncology Unit, Barzilai Medical Center, 2 Hahistadrut St, Ashkelon 78278, Israel.	Institutional Review Board, Barzilai Medical Center, 2 Hahistadrut St, Ashkelon 78278, Israel.<br><br>Chairperson- Gil Luggasi
Efrat (Ben-Baruch), Noa. MD	061547/032466	Department of Oncology, Kaplan Medical Center, P.O. Box 1, Rehovol, Israel.	Institutional Review Board, Kaplan Medical Center. P.O. Box 1. Rehovot, Israel.<br><br>Chairperson- Avraham Eliraz and Lidia Arcavi
Inbar, Moshe. MD	071923/032467	Oncology division, Breast Cancer unit. Tel Aviv	Helsinki Committee, Tel Aviv Sourasky MC. 6

Investigator	Investigator no./Center no.	Description of Research Facility, Hospital/ Institution, and Address	Name of IEC/IRB Committee, Address, Committee Chair
Jiveliouk, Irina. MD (Fomer PI)<br>Yaal-Hahoshen, Neora. MD (Fomer PI)		Sourasky MC, 6 Weizman Street Tel Aviv 64239, Israel.	Weizman street. Tel Aviv 64239, Israel.<br><br>Chairperson- Topilsky Marcel
Kaufman, Bella. MD	061545/032463	Oncology division, Breast Cancer unit, Sheba medical center, Rarnat Gan 52621 Israel.	Oncology division, Breast Cancer unit, Sheba medical center, Rarnat Gan 52621 Israel.<br><br>Chairperson- Dror Haratz and<br>Ruth Harduf
Nisenbaum, Bella. MD	106953/035255	Oncology Institute, Meir Medical Center, 59 Tsharnichovski Street. Kfar-Saba 44281, Israel.	Institutional Review Board, Meir Medical Center, 59 Tsharnichovski Street. Kfar-Saba 44281, Israel.<br><br>Chairperson- Yitshal Brener.
Peretz-Yablonski, Tamar. MD	089259/032465	Sharett institute of Oncoclogy, Hadassah-Hebrew University Medical Center, Jerusalem, 91120<br>Israel.	Helsinki Committee<br>Sharett institute of Oncoclogy<br>Hadassah-Hebrew University Medical Center, Jerusalem, 91 120 Israel.

Investigator	Investigator no./Center no.	Description of Research Facility, Hospital/ Institution, and Address	Name of IEC/IRB Committee, Address, Committee Chair
			Chairperson- Jacques Michel.
Steiner, Marianna. MD	117211/050799	Oncology department, Carmel medical center/ Lin medical center, 7 Michal Street, Haifa, Israel.	Helsinki committee, Oncology department, Carmel medical center, Haifa, Israel.<br><br>Chairperson- Ruth Kitzes.
**Italy**			
Cavanna, Luigi. MD	095729/030578	Azienda Unita Sanataria Locale di Placenza, Oncologia Medica ed Ematologia Via Taverna, 49<br>29100 Placenza, Italy.	Comitato di Bioetica, Azienda USL N.1 Sassari, Via Monte Grappa, 82 07100 Sassari, Italy.<br>Chairperson- Dr. Michele Poddighe.<br><br>Comitato Etico dell' ASL di Piacenza, Via Taverna, 49, 29100 Piacenza, Italy.<br><br>Chairperson- Prof. Enzo Poli.
Del Mastro, Lucia.MD	096442/031006	Lucia Del Mastro, MD Istituto Nazionale per la Ricerca sul Cancro Oncologia Medica A Largo	Comitato di Bioetica, Azienda USL N.1 Sassari, Via Monte Grappa, 82 07100

Investigator	Investigator no./Center no.	Description of Research Facility, Hospital/ Institution, and Address	Name of IEC/IRB Committee, Address, Committee Chair
		Rosanna Benzi, 10 16132 Genova Italy.	Sassari, Italy.<br>Chairperson- Dr. Michele Poddighe<br><br>Comitato Etico, Istituto di Ricerca a Carattere Scientific - IST<br>Largo R. Benzi, 10 16132 Genova<br>Italy.<br>Chairperson- Prof. Paolo Pronzato
Gori, Stefania. MD	038511/030990	Stefania Gori, MD Azienda Ospedaliera di Perugia<br>S.C. di Oncologia Medica, Ospedale R. Silverstrini Via G. Dottori, 1 06132 Perugia, Italy.	Comitato di Bioetica, Azienda USL N.1 Sassari, Via Monte Grappa, 82 07100 Sassari, Italy.<br>Chairperson- Dr. Michele Poddighe.<br><br>Comitato Etico delle Aziende, Sanitarie dell'Umbria Segreteria Scientifico-Amministrativa, Centro Commerciale La Galleria II Piano, Int. 37 Via Gramsci, 6 06132 Perugia, Italy.

Investigator	Investigator no./Center no.	Description of Research Facility, Hospital/ Institution, and Address	Name of IEC/IRB Committee, Address, Committee Chair
			Chairperson- Prof. Adolfo Puxeddu.
Gridelli, Cesare. MD	030281/030577	Cesare Gridelli, MD U.O. di Oncologia Medica. Azienda Ospedaliera S. G. Moscati, Contrada Amoretta - Cittadella, Ospedaliera - 83100 Avellino Italy.	Comitato di Bioetica, Azienda USL N.1 Sassari, Via Monte Grappa, 82 07100 Sassari, Italy.<br>Chairperson- Dr. Michele Poddighe.<br><br>Comitato Etico dell'Azienda Ospedaliem S. Giuseppe Moscati<br>c/o Contrada Amoretta - CitM Ospedaliem - Pal. Uffici, 831 00 Avellino, Italy.<br><br>Chairperson- Dr. Stefano La Verde.
Sarobba, Giuseppina<br><br>Farris, Antonio. MD (Former PI)	061259/030372	Antonio Farris, MD Cattedra Oncologia Medica, Universita di Sassari, Viale San Pietro, 8<br>07100 Sassari, Italy.	Comitato di Bioetica, Azienda USL N.1 Sassari, Via Monte Grappa, 82 07100 Sassari, Italy.<br><br>Chairperson- Dr. Michele Poddighe.

Investigator	Investigator no./Center no.	Description of Research Facility, Hospital/ Institution, and Address	Name of IEC/IRB Committee, Address, Committee Chair
Venturini, Marco. MD	094697/030377	Marco Venturini, MD A.O. Sacro Cuore - Don Calabria Reparto di Oncologia Via Don A. Sempreboni, 5 37024 Negrar (VR) Italy.	Comitato di Bioetica, Azienda USL N.1 Sassari, Via Monte Grappa, 82 07100 Sassari, Italy.<br>Chairperson- Dr. Michele Poddighe<br><br>Comitato di Etica Ospedale "Sacro Cuore - Don Calabria"<br>Via Don Sempreboni, 5 37024 Negrara (VR) Italy.<br>Chairperson- Dr. Fabrizio Nicolis
**Korea**			
Im, Young-Hyuck. MD, Ph.D	029133/030536	135-710 .50. Ilwon-Dong, Kangnam-Ku, Seoul, Korea, Samsung Medical Center. Korea.	Institutional Review Board of Samsung Medical Center. 135-710 .50. Ilwon-Dong, Kangnam-Ku, Seoul, Korea.<br><br>Chairperson: Lee, Suk-Koo.
Kim, Sung-Bae. MD, Ph.D	086110/030535	Asan Medical Center, 388-1, Poongnap-dong, Songpa-ku, Seoul, 138-736, Korea.	Institutional Review Board of Asan Medical Center, 388-1, Poongnap-dong, Songpa-ku,

Investigator	Investigator no./Center no.	Description of Research Facility, Hospital/ Institution, and Address	Name of IEC/IRB Committee, Address, Committee Chair
			Seoul, 138-736, Korea.<br><br>Chairperson: Sang Koo Lee.
Kim, Tae-You. MD, Ph.D	093249/030539	Seoul National University Hospital, 101 Daehang-ro (28 Yeongeon-dong), J ongno-gu, Seoul, 110-744, Korea.	Seoul National University Hospital Institutional Review Board, Seoul National University Hospital, 101 Daehang-ro (28 Yeongeon-dong), Jongno-gu, Seoul, 110-744, Korea.<br><br>Chairperson: Jae-Seung Paick
Lee, Keun Seok. MD	142961/085924	National Cancer Center, 809 Madu1-dong, Iisandong-gu, Goyang city, Gyeonggi-do, 411-769, Republic of Korea.	Institutional Review Board of National Cancer Center, 809 Madu1-dong, Iisandong-gu, Goyang city, Gyeonggi-do, 411-769, Republic of Korea.<br><br>Chairperson: Kim, Heung Tae

**Investigator**	**Investigator no./Center no.**	**Description of Research Facility, Hospital/ Institution, and Address**	**Name of IEC/IRB Committee, Address, Committee Chair**
**Lativa**			
Purkalne, Gunta. MD	000832/032397	P. Stradina kus, Pilsonu Iela 13, Riga, LV 1002, Latvia.	Paula stradina kllnlskas universitates sllmnlcas attistibas fonda zalu un farmaceitisko produktu izpetes, Etlkas komiteja, Pilsonu iela 13, Riga, LV 1002 Latvia.<br><br>Chairperson- Mr. Peteris Stradins.
Skrodele, Maija. MD	037869/032399	Piejuras slimnica, onkologiska klinika Jurmalas iela 2, liepaja. LV 3401, Latvia.	Paula stradina kllnlskas universitates sllmnlcas attistibas fonda zalu un farmaceitisko produktu izpetes, Etlkas komiteja, Pilsonu iela 13, Riga, LV 1002 Latvia.<br><br>Chairperson- Mr. Peteris Stradins.
Zvirbule, Zanete. MD	037867/032396	Rigas austrumu slimnica, latvijas onkologijas centrs, Hipokrata iela 4 Riga, LV 1079, Latvia.	Paula stradina kliniskas universitates slimnicas attistibas biedribas kliniskas izpetes etikas komiteja, Pilsonu iela 13, Riga, LV

Investigator	Investigator no./Center no.	Description of Research Facility, Hospital/ Institution, and Address	Name of IEC/IRB Committee, Address, Committee Chair
			1002, Latvia.<br><br>Chairperson- Mr. Peteris Stradins.
**Lithuania**			
Cesnaviciene, Irmante	093701/031055	Klaipeda Hospital, Oncology Department, Liepojos 49, LT-92288, Klaipeda, Lithuania.	Lithuanian Bioethics Committee, Didzioji Street. 22, LT-01128 Vilnius, Lithuania.<br><br>Chairperson- Mr. Eugenijus Gefenas.
Grigiene, Ruta. MD, Ph.D	084792/035846	Vilnius University Oncology Institute, Chemo and Radiotherapy Department, Santariskiu 1, LT-08660, Vilnius, Lithuania.	Lithuanian Bioethics Committee<br>Didzioji Street. 22, LT-01128 Vilnius Lithuania.<br><br>Chairperson- Mr. Eugenijus Gefenas.
Inciura, Arturas. MD, Ph.D	096632/031056	Kaunas Medical University Hospital, Oncology Clinic Eiveniu 2, LT-50009, Kaunas Lithuania.	Lithuanian Bioethics Committee<br>Didzioji Street. 22, LT-01128 Vilnius Lithuania.<br><br>Chairperson- Mr. Eugenijus Gefenas.

Investigator	Investigator no./Center no.	Description of Research Facility, Hospital/ Institution, and Address	Name of IEC/IRB Committee, Address, Committee Chair
**Mexico**			
Arce-Salinas, Claudia-Haydee. MD	093465/030282	Instituto Nacional de Cancerologia, Av San Fernando No.22 Colonia Seccion XVI Del. Tlalpan, Mexico City, CP 14080, Mexico.	Comite Bioetico y Comite Cientifico, Instituto Nacional de Cancerologia, Av. San Fernando No. 22 Col. Secc. XVI, Del. Tlalpan, Mexico city, 14080, Mexico.<br><br>Chairperson: Dr. Luis A. Herrera Montalvo.
Brito-Villanueva, William Orlando. MD<br>Suárez-Sahui, Tirzo. MD (Former PI)	132700/030281	Hospital Regional ISSSTE Mérida, Calle 7 S/N X 36 Col. Pensiones, Mérida, Yucatán, 97500, Mexico.	Comite de Investigacion y Bloetica, Hospital Regional ISSSTE Mérida, Calle 7 S/N X 36 Col. Pensiones, Mérida, Yucatán, 97500, Mexico.<br><br>Chairperson: Dr Miguel Angel Serapio Hernández.
Lugo Quintana, Roberto Sergio. MD	032785/030283	Hospital Christus Muguerza del Parque, Calle Dr Pedro Leal Rodriguez 1802, Col. Santa Rita, Chihuahua, 31000, Mexico.	Comite de Etica e Investigacion Christus Muguerza del Parque SA de CV, Calle Dr. Pedro Leal Rodriguez 1802, Col. Santa Rita, Chihuahua, 31000, Chih Mexico.

**Investigator**	**Investigator no./Center no.**	**Description of Research Facility, Hospital/ Institution, and Address**	**Name of IEC/IRB Committee, Address, Committee Chair**
			Chairperson: Dr. Miguel Ángel de la Peña.
Olivares-Beltran, Guillermo. MD	122237/042822	Centro Medico ABC, Sur 132 #108 Consultorio 605, Col Las Americas, Del. Alvaro Obregon,Cp 01120 Mexico DF Mexico.	Comite de Etica y Comite Institutcional de Investigacion, Centro Medico ABC, Sur 132 #108 Consultorio 605, Col Las Americas, Del. Alvaro Obregon,Cp 01120 Mexico OF Mexico.<br><br>Chairperson: Dr Bernardo Tanur-Tatz
Sánchez Forgach, Ernesto. MD<br>Pérez Puentes, Ancizar. MD (Former PI)<br>Martinez Prieto, Marcela. MD (Former PI)	098299/031848	Centro de Estudios Mastologicos S,A de C V, Avenida Paseo de la Reforma Numero 155, 1er piso, Col. Lomas de Chepultepec, Delegacion Miguel Hidalgo, DF, 11000, Mexico.	Fundacion para la Investigacion y Ensenanza Medica, Instituto de GIneco Obstetricia y de Perinatologia S A de C V, Paseo Alexander von Humboldt No. 88 3' Seccion de. Lomas Verdes, 63120, Naucalpan, Estado de Mexico, Mexico.<br><br>Chairperson: Dr. Fernando Rio

Investigator	Investigator no./Center no.	Description of Research Facility, Hospital/ Institution, and Address	Name of IEC/IRB Committee, Address, Committee Chair
			de la Loza.
**New Zealand**			
Fitzharris, Bernard. MB ChB, FRACP	088221/037021	Oncology Service, Christchurch Hospital, Riccarton Avenue, Christchurch 8001, New Zealand.	Mutli-Region Ethics Committee, Ministry of Health, Level 2, Reception, 1-3 The Terrace, Wellington, 6011, New Zealand.<br><br>Chairperson: Mr Hector Matthews.
Harvey, Vernon. MBBS, MD, FRACP, MRCS, MRCP	035472/036332	Department of Oncology, Level 6, Auckland City Hospital, Park Road, Grafton 1023, Auckland, New Zealand.	Mutli-Region Ethics Committee, Ministry of Health, Level 2, Reception, 1-3 The Terrace, Wellington, 6011, New Zealand.<br><br>Chairperson: Mr Hector Matthews.
Kuper-Hommel, Marion. MD	096201/042954	Regional Cancer Centre, Waikato Hospital. Pembroke Street, Hamilton 2001, New Zealand.	Mutli-Region Ethics Committee, Ministry of Health, Level 2, Reception, 1-3 The Terrace, Wellington, 6011, New Zealand.<br><br>Chairperson: Mr Hector Matthews.

Investigator	Investigator no./Center no.	Description of Research Facility, Hospital/ Institution, and Address	Name of IEC/IRB Committee, Address, Committee Chair
**Peru**			
Gomez, Henry MD	053310/030387	Instituto Nacional de Enfermedades Neoplásicas, Av. Angamos Este 2520, Lima 34, Peru.	Comite de Etica en Investigacion Biomedica, Instituto Nacional de Enfermedades Neoplásicas, Av. Angamos Este 2520, Lima 34, Peru.<br><br>Chairperson: Ronaldo Calderon Velasco.
Hurtado de Mendoza, Fernando MD	073419/030388	Hospital Nacional Edgardo Rebagliati Martins, Av. Edgardo Rebagliati No. 490, Jesus Maria, Lima 11, Peru.	Comite De Etica Del Hospital Nacional Edgardo Rebagliatti Martins, Av. Edgardo Rebagliati No. 490, Jesus Maria, Lima 11, Peru.<br><br>Chairperson: Julio Cesar Alfaro Mantilla
Salas, Fernando MD	073397/031268	Hopital Nacional Guillermo Almenara Irigoyen, Av.Grau 800 - La Victoria, Lima 13, Peru.	Hopital Nacional Guillermo Almenara Irigoyen, Av.Grau 800 - La Victoria, Lima 13, Peru.<br><br>Chairperson: Demetrio Molero Castro

Investigator	Investigator no./Center no.	Description of Research Facility, Hospital/ Institution, and Address	Name of IEC/IRB Committee, Address, Committee Chair
**Philippines**			
Chan, Valorie MD	062411/031277	Veterans Memorial Medical Center, Department of Oncology, Research Centre, North Avenue, Diliman, Quezon City, 1110 Philippines.	Research and Ethtcs Committee, Veterans Memorial Medical Center, North Avenue, Diliman, Quezon City, 1110 Philippines.<br><br>Chairperson: Tito C. Atienza, MD
Igama, Jasmin MD	095633/031392	Baguio General Hospital and Medical Center, Department of Medicine, Governor Pack Road, Baguio City, Benguet, 2600, Philippines.	Ethics Review Commitee, Baguio General Hospital and Medical Center, Governor Pack Road, Baguio City, Benguet, 2600 Philippines.<br><br>Chairperson: Concessa Padilla, MD
Tiangco, Beatrice MD	093514/031005	The Medical City, Unit 405, Medical Arts Tower Inc., Ortigas Avenue, Pasig City,1600 Philippines.	Research Ethics Review Board, The Medical City, Ortigas Avenue, Pasig City, 1600 Philippines.<br><br>Chairperson: Milagros T. Jocson, MD

Investigator	Investigator no./Center no.	Description of Research Facility, Hospital/ Institution, and Address	Name of IEC/IRB Committee, Address, Committee Chair
Tudtud, Dennis Ramon MD	096488/031279	Hospice Care and Patient Centre, Suite 803, SPC Medical Specialty Center, Gorordo Avenue, Cebu, 6000 Philippines.	Bioethics Committee, Perpetual Succour Hospital, SPC Medical Specialty Center, Gorordo Avenue, Cebu, 6000 Philippines.<br><br>Chairperson: Ellie May Villegas, MD
**Poland**			
Cedrych, Ida. MD<br>Rolski, Janusz. MD, Ph.D<br>(Former PI)	216372/030393	Centrum Onkologii, Instytut im. Marii Sklodowskiej-Curie, Oddzial w Krakowie, Klinika Nowotworow Ukladowych i Uogolnionych ul. Gamcarska 11, 31-115 Krakow, Poland.	Komisja Bioetyczna przy Okregowej Warminsko-Mazurskiej Izbie Lekarskiej w Olsztynie, ul. Zolnierska 16, 10-591 Olsztyn, Poland.<br><br>Chairperson- Zdzisław Piesiak.
Chmielowska, Ewa. MD, Ph.D	141958/050927	Centrum Onkologii, Oddzial Kliniczny Onkologii, ul. Izabeli Romanowskiej 2, 85-796 Bydgoszcz, Poland.	Komisja Bioethczna przy Okregowej Warminsko-Mazurskiej Izbie Lekarskiej w Olsztynie, ul. Zolnierska 16, 10-591 Olsztyn, Poland.<br><br>Chairperson- Zdzisław Piesiak.

Investigator	Investigator no./Center no.	Description of Research Facility, Hospital/ Institution, and Address	Name of IEC/IRB Committee, Address, Committee Chair
Jagiello-Gruszfeld, Agnieszka. MD, Ph.D*	040529/030394	ZOZ MSWiA z Warminsko-Mazurskim Centrurm Onkologii, ul. Wojsaka Polskiego 37 10-288 Olsztyn, Poland.	Komisja Bioetyczna przy Okregowej Warminsko-Mazurskiej Izbie Lekarskiej w Olsztynie, ul. Zolnierska 16, 10-591 Olsztyn, Poland.<br><br>Chairperson- Zdzisław Piesiak.
Jagiello-Gruszfeld, Agnieszka. MD, Ph.D	040529/055845	ONKO-MED. Oddzial Onkologii, ul. Wojska Polskiego 30, 10-226 Olsztyn, Poland.	Komisja Bioethczna przy Okregowej Warminsko-Mazurskiej Izbie Lekarskiej w Olsztynie, ul. Zolnierska 16, 10-591 Olsztyn, Poland.<br><br>Chairperson- Zdzisław Piesiak.
Sawrycki, Piotr. MD	133877/049563	Wojewodzki Szpital Zespolony Im Ludwika Rydygiera, Oddzial Onkologii Klinicznej, ul. Sw. Jozefa 53/59, 87-100 Torun, Poland.	Komisja Bioethczna przy Okregowej Warminsko-Mazurskiej Izbie Lekarskiej w Olsztynie, ul. Zolnierska 16, 10-591 Olsztyn, Poland.<br><br>Chairperson- Zdzisław Piesiak.

Investigator	Investigator no./Center no.	Description of Research Facility, Hospital/ Institution, and Address	Name of IEC/IRB Committee, Address, Committee Chair
Szczylik, Cezary. MD, Ph.D	084603/ 030392	Klinika Onkologii, Wojskowy Instytut Medyczny, Szaserow 128, 00-909 Warszawa, Poland.	Kornisja Bioetyczna przy, Okregowej Warmnsko- Mazurskiej Izbie Lekarskiej w Olszynie, ul. Zolnierska 16, 10-591 Olsztyn, Poland.<br><br>Chairperson- Zdzisław Piesiak
**Russia**			
Andreeva, Elena. MD<br>Korman, David. MD, Ph.D, DM (Former PI)	054296/033142	GUZ City Clinical Hospital # 40, Chemotherapy Department, 7, Kasatkina street, Moscow, 129301, Russia.	The Ethics Committee attached to GUZ City Clinical Hospital # 40, 7, Kasatkina street, Moscow, 129301, Russia.<br><br>Chairperson- R.I.Shaburov.<br><br>Ethics Council, Ministry of Healthcare and social development of the Russian Federation, 3, Rahmanovskij pereulok, Moscow, 127994, Russia.

Investigator	Investigator no./Center no.	Description of Research Facility, Hospital/ Institution, and Address	Name of IEC/IRB Committee, Address, Committee Chair
			Deputy Chairperson – N.A. Mikhailova.
Cheporov, Sergey. MD	087820/033144	GUZ YO Regional Ciinicai Oncology Hospital, 4A, ui.Chkalova, Yaroslavl, 150054, Russia.	The Ethcis Committee attached to the Federal Agency for Control of Quality of Medicinal Product, 8, Petrovsky Boulevard, Build 3, Moscow, 127051, Russia.<br><br>Chairperson- K.I. Tebloev.
Dobrovolskaya, Natalya. MD, Ph.D	041623/033136	FGU Russian Roentgeno-Radiology Research Center of Roszdrav. 86. Profsouznaya Street., Moscow. 117997, Russia.	The Ethics Committee attached to FGU Russian Roentgeno-Radiology Research Center of Roszdrav, 86, Profsouznaya Street., Moscow, 117997, Russia.<br>Chairperson- G.A. Panshin.<br><br>Ethics Council attached to the Ministry of Healthcare of Russian Federation, 127051, Moscow, Petrovsky bulvar, 8, bldn. 2.<br><br>Deputy Chairperson – N.A. Mikhailova.

Investigator	Investigator no./Center no.	Description of Research Facility, Hospital/ Institution, and Address	Name of IEC/IRB Committee, Address, Committee Chair
Manikhas, Alexey. MD, Ph.D	041569/033138	SPbGUZ City Clinical Oncology Dispensary, 3/5, 2nd Berezovaya alleya, St-Petersburg, 197022, Russia.	The Ethics Committee attached to the SPbGUZ City Cliniacl Oncology Discpensary, 3/5, 2nd Berezovaya alleya, St-Petersburg, 197022, Russia.<br>Chairperson- A.V. Pavlysh.<br><br>Ethics Council attached to the Ministry of Healthcare of Russian Federation, 127051, Moscow, Petrovsky bulvar, 8, bldn. 2.<br><br>Deputy Chairperson – N.A. Mikhailova.
Semiglazov, Vladimir. MD, Ph.D, DM	040179/033141	GUN Scientific Research Institute of Oncology n.a. prof. N.N.Petrov of Roszdrav, 68. Leningradskaya Street., Saint Petersburg, Pesochny-2, 197758, Russia.	The Ethics Committee attached to the GUN Scientific Research Institute of Oncology n.a. prof. N.N.Petrov of Roszdrav, 68, Leningradskaya Street., SaintPetersburg, Pesoohny-2, 197758, Russia.<br><br>Chairperson- E.V Dyomin

Investigator	Investigator no./Center no.	Description of Research Facility, Hospital/ Institution, and Address	Name of IEC/IRB Committee, Address, Committee Chair
			Ethics Council attached to the Ministry of Healthcare of Russian Federation, 127051, Moscow, Petrovsky bulvar, 8, bldn. 2.<br><br>Deputy Chairperson – N.A. Mikhailova.
Shomova, Marina. MD, Ph.D, DM	101133/033151	GUS Regional Clinical Oncology Dispensary. 13 Sportivnaya Street, Ryazan, 39001 1, Russia.	The Ethics Committee attached to the GUZ Regional Clinical Oncology Dispensary, 13 Sportivnaya Street, Ryazan, 390011, Russia. Chairperson- A.I. Smirnov<br><br>Ethics Council attached to the Ministry of Healthcare of Russian Federation, 127051, Moscow, Petrovsky bulvar, 8, bldn. 2.<br><br>Deputy Chairperson – N.A. Mikhailova.
Tjulandin, Serey. MD, Ph.D, DM	082721/033135	GU Russian Oncology Scientific Center n.a. N.N.Blokhin RAMS, Department of Clinical	The Ethics Committee attached to GU Russian Oncology Scientific Center n.a. N.N,

Investigator	Investigator no./Center no.	Description of Research Facility, Hospital/ Institution, and Address	Name of IEC/IRB Committee, Address, Committee Chair
		Pharmacology and Chemotherapy. 24, Kashirskoye shosse. Moscow, 115478, Russia.	Blokhin RAMS, 24, Kashirskoye shosse, Moscow, 115478, Russia.<br><br>Chairperson- D.Z. Kuptchan<br>Ethics Council attached to the Ministry of Healthcare of Russian Federation.<br><br>Deputy Chairperson – N.A. Mikhailova.
**Slovakia**			
Chovanec, Jozef. MD	041224/032870	Department of Oncology, Hospital with Policlinics Bardejov, Sv.Jakuba 21, 08501 Bardejov I Slovakia.	Ethics Committee Hospltal with Policlinics Bardejov, Sv. Jakuba 21, 085 01 Bardejov.<br><br>Chairperson- Martina Suchova, MD, PhD
Koza, Ivan. MD, DrSC	038176/032865	National Cancer Institute, Klenova 1, 833 10 Bratislava / Slovakia.	Institutional Review Board of National Cancer Institute, Klenova 1, 833 10 Bratislava / Slovakia.<br><br>Chairperson- Kristina Krizanova, MD.

Investigator	Investigator no./Center no.	Description of Research Facility, Hospital/ Institution, and Address	Name of IEC/IRB Committee, Address, Committee Chair
Pritzova, Eva. MD	014023/032863	Dspartment of Oncology, F.D. Roosevelt's Faculcy Hospital, Nam. L Svobodu 1, 976 17 Banska Bystrica / Slovakia.	Ethics Cornmittse F.D. Roosevelt's Faculcy Hospital, Nam, L. Svobodu 1, 975 17 Banske Bystrica, Slovakia.<br><br>Chairperson- Juraj Svac, MD, PhD.
Stresko, Marian. MD	100655/032864	Department of Radiotherapy and Medical Oncology, Faculcy Hospital Nilra, Spitalska 6, 94901 Nitra Slovakia.	Ethics Committee Faculcy Hospital Nitra, Spitalska 6, 94901, Nitra/ Slovakia.<br><br>Chairperson- Maria Goboova, PharmDr.
**South Africa**			
Barnardt, Pieter. MB, ChB	094137/030131	Tygerberg Hospital, Gene Louw Building Francie van Zijl Drive, Tygerberg 7505, South Africa.	University of Stellenbosch Committee for Pharmaceutical Trials, Office for Pharmaceutical Trials, Research, Development and Support, Room 500BA, Teaching Block, Francie van Zijl Drive Tygerberg 7505,South Africa.<br><br>Chairperson: Dr WAJ Meintjes

Investigator	Investigator no./Center no.	Description of Research Facility, Hospital/ Institution, and Address	Name of IEC/IRB Committee, Address, Committee Chair
Coetzee, Corlia. MB<br><br>Raats, Johannes. MB, ChB, PhD (Former PI)	148550/030134	GVI Oncology Clinical Research Unit, Tiger Ave, Windsor Park, Kraaifontein, Western Cape 7570<br>South Africa.	University of the Witwatersrand Human Research Ethics Committee, 8 Blackwood Avenue, Parktown, 2193, South Africa.<br><br>Chairperson: Prof PE Cleaton Jones
Cohen, Graham. MB, ChB, FCP(SA)	025585/030132	Mary Potter Oncology Centre, Little Company of Mary Hospltal, Totlus Street Groenkloof, Pretoria, 0181 South Africa.	University of the Witwatersrand Human Research Ethics Committee, 8 Blackwood Avenue, Parktown, 2193, South Africa.<br><br>Chairperson: Prof PE Cleaton Jones.
Jones, Lee-Ann. MB, ChB, (FC(Rad Onc)	093602/030140	Langenhoven Drive Oncology Centre, 1 Mangold street Newtown Park, Port Elizabeth, 6045 South Africa.	University of the Witwatersrand Human Research Ethics Committee, 8 Blackwood Avenue, Parktown, 2193, South Africa.<br><br>Chairperson: Prof PE Cleaton Jones.
Landers, Gregory. MB, ChB,MMed	034241/030141	The Oncology Centre, 535 Peter Mokaba Road, Overport, Durban, 4091, South Africa.	University of the Witwatersrand Human Research Ethics Committee, 8 Blackwood Avenue, Parktown, 2193, South Africa.

Investigator	Investigator no./Center no.	Description of Research Facility, Hospital/ Institution, and Address	Name of IEC/IRB Committee, Address, Committee Chair
			Chairperson: Prof PE Cleaton Jones
Rapoport, Bernardo. MD	093006/030135	The Medical Oncology Centre of Rosebank, 129 Oxford Road, Cm Oxford & Northwold rds Saxonwold, 2196 South Africa.	University of the Witwatersrand Human Research Ethics Committee, 8 Blackwood Avenue, Parktown, 2193, South Africa.<br><br>Chairperson: Prof PE Cleaton Jones.
Ruff, Paul. MB, Bch, MMed(Int Med), FCP(SA)	023258/030136	University of Witwatersrand, Faculty of Health Sciences, Dept Medical Oncology, Johannesburg Hospital, Area 495, 7 York Road, Parkiown, 2193 South Africa.	University of the Witwatersrand Human Research Ethics Committee, 8 Blackwood Avenue, Parktown, 2193, South Africa.<br><br>Chairperson: Prof PE Cleaton Jones.
Ruff, Paul. MB, Bch, MMed(Int Med), FCP(SA)	023258/030137	University Witwatersrand Oncology, 17 and 18 Etan Road, Parktown, 2193, South Africa.	University of the Witwatersrand Human Research Ethics Committee, 8 Blackwood Avenue, Parktown, 2193, South Africa.<br><br>Chairperson: Prof PE Cleaton Jones.

Investigator	Investigator no./Center no.	Description of Research Facility, Hospital/ Institution, and Address	Name of IEC/IRB Committee, Address, Committee Chair
Szpak, Waldemar. MB.<br>Pirjol, Anca. MB (Former PI)	093176/030133	Dr's Pirjol & Szpak Private Oncology Practice, 315 Ipahla Road, Athlone Park Amanzimtoti, 4126 South Africa.	University of the Witwatersrand Human Research Ethics Committee, 8 Blackwood Avenue, Parktown, 2193, South Africa.<br><br>Chairperson: Prof PE Cleaton Jones.
Vorobiof, Daniel. MD	051420/030130	Sandton Oncology Centre, 159 Rivonia Rd Morningside, Sandton 2199, South Africa.	University of the Witwatersrand Human Research Ethics Committee, 8 Blackwood Avenue, Parktown, 2193, South Africa.<br><br>Chairperson: Prof PE Cleaton Jones.
**Spain**			
Albanell, Joan. MD, PhD	041436/030501	Hospital del Mar. Paseo Maritimo 25-29, Ed.Itaca, 08003 Barcelona, Spain.	Comite Etico delInstituto Municipal de Asistencia Sanitaria (IMIM), C/ Doctor Aiguader 80, 08003 Barcelona, Spain.<br><br>Chairperson- Magí Farré Albaladejo.
Ales, Jose E. MD, PhD	030076/030496	Hospital Ruber Internacional. Servicio dn Oncologia Medica, C/La Maso 38 28034	Comite Etico de Investigacion Clinica, Hospital Ruber Internacional, Servicio dn

Investigator	Investigator no./Center no.	Description of Research Facility, Hospital/ Institution, and Address	Name of IEC/IRB Committee, Address, Committee Chair
		Madrid, Spain.	Oncologia Medica, C/La Maso 38 28034 Madrid, Spain.<br><br>Chairperson- Angel Ruiz de Aguiar.
Anton, Antonio. MD, PhD	010554/030482	Hospital Miguel Servet, Servicio de Oncologia, P° Isabel la Catolica 1-3, 50009 Zaragoza, Spain.	CEIC Hospital Miguel Servet. Avda. Gomez Laguna 25, planta 3.50009 Zaragoza, Spain.<br><br>Chairperson- María González Hinjos.<br><br>Comite Etico de Investigacion Clinica, Hospital Universitario de Bellvitge C/Feixa Llarga s/n (Frente a Urgencias), Edificio Fundacio August PI i Sunyer<br>08907 Hospitalet de Llobregat (Barcelona), Spain.<br><br>Chairperson- María González Hinjos.

Investigator	Investigator no./Center no.	Description of Research Facility, Hospital/ Institution, and Address	Name of IEC/IRB Committee, Address, Committee Chair
Avella Mestre, Antoni. MD<br>Rifá, Julio. MD (Former PI)	125166/030484	Hospital Universitari Son Espases, Ctra. de Valldemossa 79, 07010 Palma de Mallorca Spain.	Comite Etico de Investigacion Clinica, Hospital Universitario de Bellvitge, Edkficio de Consultas Extemas, planta 1º C/Feixa Llarga s/n, 08907 Hospitalet de Llobregat, Barcelona, Spain.<br><br>Chairperson- Joan Bargay Lleonat.<br><br>Llles Baleares Clinical Research Ethics Commitee, Conselleria de Salut i Consum, C/ Cecil Metel 18, 07003 Palma de Mallorca, Spain.<br><br>Chairperson- Joan Bargay Lleonat.
Batista, Jose. MD, PhD	010323/030485	Hospital Universitario de Canarias, Servicio de Oncotogia, C/Offa sin. La Laguna 38320 T enerife, Spain.	Comite Etico de Investigaci6n Clinica del Hospital Universitario de Canarias CI Ofra s/n- La Cuesta, Pabe1l6n de Gobierno 31

Investigator	Investigator no./Center no.	Description of Research Facility, Hospital/ Institution, and Address	Name of IEC/IRB Committee, Address, Committee Chair
			Planta La Laguna, 38320 Tenetife Spain.<br><br>Chairperson- Mª del Mar García Sáez.
Brunet, Joan. MD, PhD<br>Colomer, Ramon. (Former PI)	118691/030489	Hospital Universitario Dr. Josep Trueta, Servicio de Oncologia, Avda. De Francia s/n, 17007 Girona, Spain.	Comite Etico de Investigacion Clinica, Hospital Universitario Dr. Josep Trueta Avenida de Francia s/n planta 9ª A, 17005 Girona, Spain.<br><br>Chairperson- Ferrán García-Bragado.<br><br>Comite Etico de Investigacion Clinica, Hospital Un iversitario de Bellvitge C/Feixa Llarga s/n (Frente a Urgencias), Edificio Fundacio August Pi i Sunyer<br>08907 Hospitalet de Llobregat (Barcelona), Spain.<br><br>Chairperson- Ferrán García-Bragado.

Investigator	Investigator no./Center no.	Description of Research Facility, Hospital/ Institution, and Address	Name of IEC/IRB Committee, Address, Committee Chair
Catalan, Gustavo. MD	095825/031362	Hospital Son Llatzer, Servicio de Oncologia, Carretera de Manacor Km. 4, 07198 Palma de Mallorca, Spain.	Comite Etico de Investigacion Clinica de Baleares Conselleria de Salut i Consum, C/ Cecili Metel 18 07003 Palma de Mallorca, Spain.<br><br>Chairperson- Joan Bargay Lleonat.<br><br>Comite Etico de Investigacion Clinica, Hospital Universitario de Bellvitge, C/Feixa Llarga s/n (Frente a Urgencias), Edificio Fundacio August Pi i Sunyer, 08907 Hospitalet de Liobregat (Barcelona), Spain.<br><br>Chairperson- Joan Bargay Lleonat.
Cortes, Javier. MD, PhD	055507/031394	Hospilal Vall d' Hebron. P° Vall d' Hebron 119-129, Ed. General planta baja, Servicio de Oncologia, 08035 Barcelona. Spain.	Ethics Committee of Clinical Research, Hospital Vall d'Hebron<br>Direccion de Investigacion, Edificio Institu de Recerca, 2ª planta, P° Vall d' Hebron 119-

Investigator	Investigator no./Center no.	Description of Research Facility, Hospital/ Institution, and Address	Name of IEC/IRB Committee, Address, Committee Chair
			129, 08035 Barcelona, Spain.<br><br>Chairperson- Soledad Gallego Melcón
Garcia Mata, Jesus. MD*	093240/030495	Hospital Cristal Pinol, Oncology Department. C/Ramon Puga 54, 32005 Orense, Spain.	Servicio Gallego de Salud, Comite Etico de Investigacion Clinica, Edificio Administrativo San Lazaro s/n Santiago de Compostela, 15703 A Coruna. Spain.<br><br>Chairperson- Rosendo Bugarin González.
García Saenz, Jose Angel. MD<br>Olmos, Miguel. MD, PhD<br>(Former PI)	078089/030499	Hospital Clinico San Carlos, Oncology Department<br>C/Profesor Martin Lagos s/n<br>28040 Madrid, Spain.	Comite Etica de Investigacion Cllnica Regional de la Comunidad de Madrid C/ Aduana, 29, 28013 Madrid, Spain.<br><br>Chairperson- Alfonso Moreno González.
García Saenz, Jose Angel<br>Gil, Miguel. MD	041286/030478	ICO Bellvitge, Avda. Castelldefells Km. 2.7, Hospitalet de Llobregat, 08907 Barcelona, Spain.	Comite Etico de Investigacion Clinica, Hospital Universitario de Bellvitge C/Feixa Llarga s/n (Frente a Urgencias)<br>Edificio Fundacio August Pi i Sunyer 08907

Investigator	Investigator no./Center no.	Description of Research Facility, Hospital/ Institution, and Address	Name of IEC/IRB Committee, Address, Committee Chair
			Hospitalet de Llobregat Barcelona, Spain.<br><br>Chairperson- Francesc Esteve Urbano.
Jara, Carlos	040284/030541	Fundacian Hospital de Alcorcan, Servicio de Oncologia, C/Budapest 1, Alcorcan, 28922 Madrid, Spain.	Comite Etico de Investigacion Clinica de la Fundacion Hospital de Alcorcan C/ Budapest 1, Planta Satano Alcorcan<br>28922 Madrid, Spain.<br><br>Chairperson- Patricia Sanmartín Fenollera.
Lianes, Pilar. MD, Ph.D	010375/030491	Hospital de Mataro, Servicio de Oncologia, Carretera de Cirera s/n Mataro, 08034 Barcelona, Spain.	Comite Etico de Investigacion Clinica del Consorcio Sanitario del Maresme, Carretera de Cirera s/n Planta -2 Unidad de Recerca, 08034, Barcelona, Spain.<br><br>Chairperson- Mateu Serra Prat.
Lluch Hernandez, Ana. MD	041442/030498	Hospital Clinico de Valencia,Medical Oncology Department, Avda Blasco Ibanez, 17 46010 Valencia, Spain.	Ethics Committee of Clinical Research, Hospital Clinico de Valencia, Avda Blasco Ibanez, 17 46010 Valencia, Spain.

Investigator	Investigator no./Center no.	Description of Research Facility, Hospital/ Institution, and Address	Name of IEC/IRB Committee, Address, Committee Chair
			Chairperson- Antonio Peláez Hernández.
López de Ceballos, Helena. MD	097914/030493	Hospital San Pedro de Alcantara, Avda. Pablo Naranjo sIn, 10003 Caceres, Spain.	Clinical Research Etics Committee, Complejo Hospitalario San Pedro de Alcantara, Avenida Pablo Naranjo sIn, 10003 Caceres, Spain.<br><br>Chairperson- Luis Palomo Cobos.
Lopez Vega, Manuel. MD, Ph.D	095829/031393	Hospital Marques de Valdecilla, Servicio da Oncologia, Avda. de Valdecilla s/n<br>39008 Santander, Spain.	Comite Etico de Investigacion Clinica de Cantabria Fundacion Marques de Valdecilla, Escuela de Enfermeria Planta 5° Avenida de Valdecilla s/n, 39008 Santander, Spain.<br><br>Chairperson- Carlos Redondo Figuero.<br><br>Comite Etico de Investigacion Clinica, Hospital Universitario de Bellvitge, C/Feixa Llarga s/n (Frente a Urgencias), Edificio

Investigator	Investigator no./Center no.	Description of Research Facility, Hospital/ Institution, and Address	Name of IEC/IRB Committee, Address, Committee Chair
			Fundacio August Pi i Sunyer, 08907 Hospitalet de Llobregat (Barcelona), Spain.<br><br>Chairperson- Carlos Redondo Figuero.
Lopez, Rafael. MD	093241/031397	Hospital Clinico Universitario de Santiago, C/Choupana s/n, Santiago de Compostela, 15706 A Coruna, Spain.	Comite Etico de Investigacion Clinica del Servicio Galego de Salud (SERGAS) Edificio Administrative San Lazaro s/n<br>Santiago de Compostela, 15703 A Corufia. Spain.<br><br>Chairperson- Rosendo Bugarin González.
Morales, Serafin. MD<br>Llombart, Antonio. MD (Former PI)	010280/031395	Hospital Arnau de Vilanova, Oncology Service, Edificio Viejo 3er piso, Avda. Alcalde Rovira Roure 44, 25198 Lleida, Spain.	Cllnical Research Ethics Committee, Hospital Amau de Vilanova Hospital Amau de Villanova, Ediflcio Viejo 3er piso, Avda. Alcalde Rovira Roure, 44 25198 Llelda, Spain.<br><br>Chairperson- Joan Antoni Schoenenberger.

Investigator	Investigator no./Center no.	Description of Research Facility, Hospital/ Institution, and Address	Name of IEC/IRB Committee, Address, Committee Chair
Morales, Serafin<br><br>Llombart, Antonio (Former PI)<br><br>Sanchez Rovira, Pedro. MD, PhD	040280/030480	Dr. Pedro Sanchez Rovira, Complejo Hospitalario de Jaen, Servicio de Oncologia, Planta baja, Avda. del Ejercito Espanol 10, 23007 Jaen, Spain.	Comite Etico de Investigacion Clinica Regional Andaluz, Consejeria de Salud, Avenida de la Innovacion s/n, Edificio Arena 1, 41020 Sevilla, Spain.<br><br>Chairperson- Jesús Foronda Bengoa.
**Ukraine**			
Banakhevych, Natalyia. MD	101658/034163	Kiev City Oncology Hospital 69. Verkhovinna Street., Kyiv 031 15, Ukraine	State Pharmacological Centre. Ministry of Health of Ukraine, 40 Ushinskyi Street, Kyiv 03151, Ukraine.<br>Chairperson- Mykhaylo Nesterchuk.<br><br>Local Ethics Committee, Kiev City Oncology Hospital 69. Verkhovinna Street, Kyiv 031 15, Ukraine.<br><br>Chairperson- Hordiychuk Prokop.

Investigator	Investigator no./Center no.	Description of Research Facility, Hospital/ Institution, and Address	Name of IEC/IRB Committee, Address, Committee Chair
Bondarenko, Igor. MD, PhD, DSc	080493/033689	City Clinial Hospital # 31, Blizhnaya Street Dnepropetrovsk 49102. Ukraine.	State Expert Centre. Ministry of Health of Ukraine, 40 Ushinskyi Street, Kyiv 03151, Ukraine.<br><br>Chairperson- Mykhaylo Nesterchuk.<br><br>Local Ethics Committee, City Clinial Hospital # 431, Blizhnaya Street Dnepropetrovsk 49102, Ukraine.<br><br>Chairperson- Shynkarenko Mykola.
Hotko, Yevhen. MD, PhD	101413/034438	Transcarpathian Regional Oncology Clinical Dispensary 2. Brodlakovicha Street., Uzhgorod 88014, Ukraine.	State Expert Centre. Ministry of Health of Ukraine, 40 Ushinskyi Street, Kyiv 03151, Ukraine.<br>Chairperson- Mykhaylo Nesterchuk.<br><br>Local Ethics Committee, Uzhgorod Central City Clinical Hospital 20, Gryboiedov Street.,

Investigator	Investigator no./Center no.	Description of Research Facility, Hospital/ Institution, and Address	Name of IEC/IRB Committee, Address, Committee Chair
			Uzhgorod 88017, Ukraine.<br><br>Chairperson- Volodymyr Shpontak.
Shparyk, Yaroslav. MD, PhD	002259/033688	Lviv State Regional Oncology Medical and Diagnostic Centre, 2A. Hashek Street., Lviv 79031, Ukraine	State Expert Centre. Ministry of Health of Ukraine, 40 Ushinskyi Street., Kyiv 03151, Ukraine.<br>Chairperson- Mykhaylo Nesterchuk.<br><br>Local Ethics Committee, Lviv State Regional Oncology Medical and Diagnostic Centre 2A. Hashek Street., Lviv 79031, Ukraine.<br><br>Chairperson- Yurii Diychuk.
**United Kingdom**			
Agrawal, Rajiv. MB, DNRT, MD, FRCR	040286/031609	The Shrewsbury and Telford Hospital NHS Trust, consisiting of The Royal Shrewsbury Hospital,<br>Mytton Oak Road Shrewsbury, SY3 8XQ ,	West Glasgow Ethics Committee (1)<br>Administration Building<br>Western Infirmary, Dumbarton Road, Glasgow, Scotland, G11 6NT, United

Investigator	Investigator no./Center no.	Description of Research Facility, Hospital/ Institution, and Address	Name of IEC/IRB Committee, Address, Committee Chair
		United Kingdom.	Kingdom.<br><br>Chairperson- Dr John Hunter.
Anand, Anjana.FRCR, MSc (ONC), MRCP, MBBS<br>Ahmed, Samreen I.MD (Former PI)	108879/031029	Nottingham University Hospital NHS Trust, City Hospital Campus, Hucknall Road, Nottingham NGS 1PB, United Kingdom.	West Glasgow Ethics Committee (1)<br>Administration Building<br>Western Infirmary, Dumbarton Road, Glasgow, Scotland, G11 6NT, United Kingdom.<br><br>Chairperson- Dr John Hunter.
Armstrong, Anne. BSe (Hons), MB ChB, MRCP, PhD<br>Wardley, Andrew (Former PI)	095741/035603	Christie Hospital NHS Trust, 550 Wilmslow Road, Manchester, M20 4BX, United Kingdom.	West Glasgow Ethics Committee (1)<br>Administration Building<br>Western Infirmary, Dumbarton Road, Glasgow, Scotland, G11 6NT, UK.<br><br>Chairperson- Dr John Hunter.
Bundred, Nigel. MB BS, FRCS, MD	112882/037473	Wythenshawe Hospitel, Nightingale genesis Centre, Southmoor Road. Wythenshawe.	West Glasgow Ethics Committee (1)<br>Administration Building Western Infirmary,

Investigator	Investigator no./Center no.	Description of Research Facility, Hospital/ Institution, and Address	Name of IEC/IRB Committee, Address, Committee Chair
		MancMster, M23 9L T. United Kingdom.	Dumbarton Road, Glasgow, Scotland, G11 6NT, United Kingdom.<br><br>Chairperson- Dr John Hunter.
Bundred, Nigel. MB BS, FRCS, MD<br><br>Canney, Peter. FRCR, MBChB, MD	102618/33827	The Beatson West of Scotland Cancer Centre, Level 0, 1053 Great Western Road, Glasgow, G12 OYN, United Kingdom.	West Glasgow Ethics Committee (1) Administration BuildingWestern Infirmary, Dumbarton Road, Glasgow, Scotland, G11 6NT, United Kingdom.<br><br>Chairperson- Dr John Hunter
Coleman, Robert. FRCP, MBBS, MRCP, MD	033602/031605	Weston Park Hospital, Whitham Road, Sheffield, S10 2SJ, United Kingdom.	West Glasgow Ethics Committee (1) Administration BuildingWestern Infirmary, Dumbarton Road, Glasgow, Scotland, G11 6NT, United Kingdom.<br><br>Chairperson- Dr John Hunter.
Davidson, Neville. MBBS	001218/031031	Mid Essex Hospitals NHS Trust, Oncology Research, Ground Floor, West Wing Two,	West Glasgow Ethics Committee (1) Administration Building Western Infirmary,

Investigator	Investigator no./Center no.	Description of Research Facility, Hospital/ Institution, and Address	Name of IEC/IRB Committee, Address, Committee Chair
		Broomfield Hospital, Court Road, Chelmsford, Essex CM1 7ET, United Kingdom.	Dumbarton Road, Glasgow, Scotland, G11 6NT, United Kingdom.<br><br>Chairperson- Dr John Hunter.
Harper-Wynne, Catherine. MD, MRCPI, MBBS	042257/033286	Kent Oncology Centre, Maidstone Hospital, Hermitage Lane, Barming, Maidstone Kent, ME16 9QQ, United Kingdom.	West Glasgow Ethics Committee (1) Administration BuildingWestern Infirmary, Dumbarton Road, Glasgow, Scotland, G11 6NT, United Kingdom.<br><br>Chairperson- Dr John Hunter.
Harries, Mark. MA PhD MRCP	084653/034199	Medical Oncology Bermondsey Wing, Guy's & Saint Thomas' NHS Foundation Trust, Great Maze Pond, London SE1 9RT, United Kingdom.	West Glasgow Ethics Committee (1) Administration BuildingWestern Infirmary, Dumbarton Road, Glasgow, Scotland, G11 6NT, United Kingdom.<br><br>Chairperson- Dr John Hunter.
Hickish, Tamash. MA, MD, FRCP	001500/031032	Royal Bournemouth Hospital NHS Trust, Castle Lane East, Bournemouth, Dorset, BH7	West Glasgow Ethics Committee (1) Administration BuildingWestern Infirmary,

Investigator	Investigator no./Center no.	Description of Research Facility, Hospital/ Institution, and Address	Name of IEC/IRB Committee, Address, Committee Chair
		7DWm United Kingdom.	Dumbarton Road, Glasgow, Scotland, G11 6NT, United Kingdom.<br><br>Chairperson- Dr John Hunter.
Joffe, Johnathan. MBBS, MD, FRCP, MRCP	033604/031033	Caulderdale and Huddersfield NHS Trust, Acre Street, Lindley, Huddersfield, HD3 3EA, United Kingdom.	West Glasgow Ethics Committee (1) Administration BuildingWestern Infirmary, Dumbarton Road, Glasgow, Scotland, G11 6NT United Kingdom.<br><br>Chairperson- Dr John Hunter.
Johnston, Stephen. MBBS	038785/033576	The Royal Marsden NHS Foundation Trust, Downs Road, Sutton, SM2 5PT United Kingdom.	West Glasgow Ethics Committee (1) Administration BuildingWestern Infirmary, Dumbarton Road, Glasgow, Scotland, G11 6NT, United Kingdom.<br><br>Chairperson- Dr John Hunter.
Jones, Alison. MB, ChB, MRCP, MD, FRCP	049326/031618	Royal Free Hospital, Pond Street, Hampstead, London, NW3 2QG, United Kingdom.	West Glasgow Ethics Committee (1) Administration BuildingWestern Infirmary,

Investigator	Investigator no./Center no.	Description of Research Facility, Hospital/ Institution, and Address	Name of IEC/IRB Committee, Address, Committee Chair
			Dumbarton Road, Glasgow, Scotland, G11 6NT, United Kingdom.<br><br>Chairperson- Dr John Hunter.
Rea, Daniel. BSc(Hons) MBBS, MRCP, CCST, PhD, FRCP	033607/035860	Cancer Centre, Queen Elizabeth Hospital, Vincent Drive, Edgbaston, Birmingham, B15 2TH, United Kingdom.	West Glasgow Ethics Committee (1) Administration BuildingWestern Infirmary, Dumbarton Road, Glasgow, Scotland, G11 6NT, United Kingdom.<br><br>Chairperson- Dr John Hunter.
Stein, Rob.	060032/038853	Department of Oncology UCL Hospitals, First Floor Central 250 Euston Road. London, NWI 2PG, United Kingdom.	West Glasgow Ethics Committee (1) Administration BuildingWestern Infirmary, Dumbarton Road, Glasgow, Scotland, G11 6NT, United Kingdom.<br><br>Chairperson- Dr John Hunter.
Verrill, Mark	049313/031034	Clinical Research Facility, Newcastle upon Tyne Hospitals NHS Trust, Royal Victoria	West Glasgow Ethics Committee (1) Administration BuildingWestern Infirmary,

Investigator	Investigator no./Center no.	Description of Research Facility, Hospital/ Institution, and Address	Name of IEC/IRB Committee, Address, Committee Chair
		Infirmary, 4th Floor, Leazes Wing, RVI, Newcastle, NE1 4LP, United Kingdom.	Dumbarton Road, Glasgow, Scotland, G11 6NT, United Kingdom.<br><br>Chairperson- Dr John Hunter.
**United States**			
Anderson, Thomas. MD	057691/031411	Texas Oncology, 1615 Hospital Parkway, Suite 300 Bedford, Texas 76022, United States.	US Oncology, Inc. Institutional Review Board, 10101 Woodloch Forest, The Woodlands, Texas 77380, United States.<br><br>Chairperson- Elizabeth Rogg, M.D.
Asbury, Robert MD	054773/037973	Interlakes Oncology & Hematology, 211 White Spruce Boulevard, Rochester, New York, 14623, United States.	Copernicus Group IRB, One Triangle Drive, Suite 100, PO Box 110605, Research Triangle Park, North Caroline, 27703, United States.<br><br>Chairperson- Glenn C. Veit, JD, CIP
Baltz, Brad. MD	093205/032643	Hematology Oncology Services of Arkansas, 9101 Kanis Road. Suite 100, Little Rock,	Copernicus Group IRB, One Triangle Drive Suite 100, PO box 110605, Research

Investigator	Investigator no./Center no.	Description of Research Facility, Hospital/ Institution, and Address	Name of IEC/IRB Committee, Address, Committee Chair
		Arizona 72205, United States.	Triangle Park, North Carolina 27709, United States.<br><br>Chairperson- Glenn C. Veit, JD, CIP.
Beck, Joseph Thaddeus MD	090697/036664	Highlands Oncology Group, 3232 N. North Hills Boulevard, Fayetteville, Arizona 72703, United States.	Copernicus Group IRB, One Triangle Drive, Suite 100 P. 0. Box 110605, Research Triangle Park, North Caroline 27709, United States.<br><br>Chairperson- Glenn C. Veit, JD, CIP
Blachly, Ronald. MD	026926/032530	NEA Baptist Clinic, 311 East Matthews, Jonesboro, Arizona 72401, United States.	Copernicus Group IRB, One Triangle Drive, Suite 100PO Box 110605, Research Triangle Park, North Caroline 27703, United States.<br><br>Chairperson- Glenn C. Veit, JD, CIP.
Block, Caroline. MD	007218/039043	New England Hematology/Oncology Associates, P.C. Vernon Cancer Center, 2014 Washington Street, Newton, Massachusettes	Human Research and Investigation Committee, Newton-Wellesley Hospital Ellison Building 2nd Floor, 2014 Washington

Investigator	Investigator no./Center no.	Description of Research Facility, Hospital/ Institution, and Address	Name of IEC/IRB Committee, Address, Committee Chair
		02462, United States.	Street, Newton, Massachusettes 02462, United States.<br><br>Chairperson- Fred Millham
Blum, Joanne. MD	058153/032531	Texas Oncology-Baylor Charles A. Sammons Cancer Center 3535 Worth Street Dallas, Texas 75246, United States.	US Oncology, Inc. Institutional Review Board, 10101 Woodloch Forest, The Woodlands, Texas 77380, United States.<br><br>Chairperson- Elizabeth Rogg, M.D.
Bosserman, Linda. MD	035834/037631	Wilshire Oncology Medical Group, Inc. 1502 Arrow Highway La Verne, California 91750, United States.	Western Institutional Review Board 3535 Seventh Avenue SW Olympia, Washington 98502, United States.<br><br>Chairperson- Theodore D. Schultz.
Bowers, Barbara. MD	037811/041971	Southdale Cancer Clinic, University of Minnesota, Fairview – Edina, 6363 France Avenue South, Suite 610, Edina, Minnesota 55435, United States.	Research Subjects' Protection Programs, MMC 820, 420 Delaware Street, Minneapolis, Minnesota 55455, United States.

Investigator	Investigator no./Center no.	Description of Research Facility, Hospital/ Institution, and Address	Name of IEC/IRB Committee, Address, Committee Chair
			Chairperson- FWA#: 00000312
Camacho, Elber. MD	073586/031412	Comprehensive Cancer Center, 1180 N. Indian Canyon Drive, Suite E218, Palm Springs, California 92262, United States.	Desert Regional Medical Center IRB, 1150 N. Indian Canyon Drive, Palm Springs, California 92262, United States.<br><br>Chairperson- Robert Rosser MD
Carroll, Robert. MD	026303/032175	Robert R. Carroll, MD, PA<br>6400 W. Newberry Road, Suite 206, Gainesville, Florida 32605, United States.	Copernicus Group IRB, One Triangle Drive Suite 100, PO box 110605, Research Triangle Park, North Carolina 27709, United States.<br><br>Chairperson- Glenn C. Veit.
Cartwright, Thomas. MD	057773/033091	Ocala Oncology Center, 433 S.W. 10th Street, Ocala, Florida 34474, United States.	US Oncology, Inc. Institutional Review Board, 4144 N. Central Expressway, Suite 1250 Dallas, Texas 7520, United States.<br><br>Chairperson- Elizabeth Rogg, M.D.

Investigator	Investigator no./Center no.	Description of Research Facility, Hospital/ Institution, and Address	Name of IEC/IRB Committee, Address, Committee Chair
Charu, Veena. MD	104393/034491	Pacific Cancer Medical Center, Inc. 1801 West Romneya Drive, Suite 203, Anaheim, California 92801, United States.	Copernicus Group IRB, One Triangle Drive, Suite 100, PO Box 110605, Research Triangle Park, North Carolina 27703, United States.<br><br>Chairperson- Glenn C. Veit, JD, CIP
Dakhil, Shaker. MD	115846/038901	Cancer Center of Kansas, 818 N Emporia, Suite 403, Wichita, Kansas 67214, United States.	Copernicus Group IRB, One Triangle Drive, Suite 100, P.O. Box 110605, Research Triangle Park, North Carolina 27709, United States.<br><br>Chairperson- Glenn C. Veit, JD, CIP
Dice, Yuhoe. MD	058586/032533	Cancer Care Centre of South Texas - HOAST, 1448 East Common Street, New Braunfels, Texas 78130, United States.	US Oncology, Inc. Institutional Review Board, 10101 Woodloch Forest, The Woodlands, Texas 77380, United States.<br><br>Chairperson- Elizabeth Rogg, M.D.

Investigator	Investigator no./Center no.	Description of Research Facility, Hospital/ Institution, and Address	Name of IEC/IRB Committee, Address, Committee Chair
Dreisbach, Luke. MD	112918/037904	Luke P. Dreisbach, MD, 39800 Bob Hope Drive Suite C, Rancho Mirage, California 92270, United States.	Copernicus Group IRB, One Triangle Drive Suite 100, PO Box 110605, Research Triangle Park, North Carolina 27703, United States.<br><br>Chairperson- Glenn C. Veit, JD, CIP
Drengler, Ronald. MD	021362/034089	South Texas Oncology and Hematology, P.A.; Research Department; 4383 Medical Dr. San Antonio, Texas 78229, United States.	Copernicus Group IRB, One Triangle Drive, Suite 100, PO Box 110605, Research Triangle Park, North Carolina 27703, United States.<br><br>Chairperson- Glenn C. Veit, JD, CIP
Ellison, David. MD	101508/033624	Charleston Hematology Oncology, PA, 125 Doughty Street Suite 500<br>Charleston, South Carolina 29403, United States.	Copernicus Group IRB, One Triangle Drive Suite 100, PO box 110605, Research Triangle Park, North Carolina 27709, United States.<br><br>Chairperson- Glenn C. Veit, JD, CIP

Investigator	Investigator no./Center no.	Description of Research Facility, Hospital/ Institution, and Address	Name of IEC/IRB Committee, Address, Committee Chair
Encarnacion, Carlos. MD	094015/033328	Texas Oncology – Waco, 1700 West Highway. 6 Waco, Texas 76712, United States.	US Oncology, Inc. Institutional Review Board, 10101 Woodloch Forest, The Woodlands, TX 77380.<br><br>Chairperson- Elizabeth Rogg, M.D.
Fain, Jerry. MD<br>Kerr, Robert. MD (Former PI)	112233/032181	Texas Oncology, PA Centres, Central Austin: 901 West 38th Street, Suite 200, Austin, Texas 78705, United States.	Copernicus Group IRB, One Triangle Drive Suite 100, PO box 110605, Research Triangle Park, North Carolina 27709, United States.<br><br>Chairperson- Glenn C. Veit, JD, CIP
Flores, Maria Regina. MD<br>Alemany, Carlos MD (Former PI)<br>Danso, Michael MD (Former PI)	095299/035284	Cancer Centers of Florida, 70 West Gore Street, Orlando, Florida 32806, United States.	US Oncology, Inc. Institutional Review Board, 10101 Woodloch Forest, The Woodlands, Texas 77380, United States.<br><br>Chairperson- Elizabeth Rogg, M.D.
Flynn, Patrick J. MD	008751/036714	Minnesota Oncology Hematology, P.A. Piper Building, 913 East 26th Street, Suite 405,	US Oncology, Inc. Institutional Review Board, 4144 North Central Expressway, Suite

Investigator	Investigator no./Center no.	Description of Research Facility, Hospital/ Institution, and Address	Name of IEC/IRB Committee, Address, Committee Chair
		Minneapolis, Minnesota 55404, United States.	1250,Dallas, Texas 75204, United States.<br><br>Chairperson- Elizabeth Rogg, M.D.
Frank, Richard. MD	040894/037628	Norwalk Hospital, 24 Stevens Street, Norwalk, Connecticut 06856, Untied States.	Norwalk Hospital IRB, 34 Maple Street Norwalk, Connecticut 06856, United States.<br><br>Chairperson- Pat Toni and Saraswathi Nair.
Garbo, Lawrence. MD	073399/034492	New York Oncology Hematology, P.C., 400 Patroon Creek Boulevard, Suite 1, Albany, New York 12206, United States.	US Oncology, Inc. Institutional Review Board, 10101 Woodloch Forest, The Woodlands, Texas 77380, United States.<br><br>Chairperson- Elizabeth Rogg.
Goldberg, Jonathan. MD	108950/036004	Mount Kisco Medical Group, 90 South Bedford Street, Mount Kisco New York 10549, Untied States.	Northern Westchester Hospital<br>400 East Main Street, Mount Kisco, New York 10549, Untied States.<br><br>Chairperson- Craig Brandt.
Graham, Charles. MD	101063/033162	Charleston Cancer Center, 2910 Tricom	Copernicus Group IRB, 118 MacKenan Drive,

Investigator	Investigator no./Center no.	Description of Research Facility, Hospital/ Institution, and Address	Name of IEC/IRB Committee, Address, Committee Chair
		Street, Charleston, South Carolina 29406, United States.	Suite 400, Cary, North Carolina 27511, United States.<br>Chairperson- Glenn C. Veit, JD, CIP.
Halibey, Bohdan. MD	064734/035798	Sparta Cancer Center, 89 Sparta Ave. Suite 130<br>Sparta, New Jersy 07871, United States.	Copernicus Group IRB, One Triangle Drive, Suite 100,P.O. Box 110605, Research Triangle Park, North Carolina 27709, United States.<br><br>Chairperson- Glenn C. Veit, JD.
Hallmeyer, Sigrun. MD	094897/035278	Oncology Specialists, S.C., 1700 Luther Lane, Park Ridge, Illinois 60068, United States.	Western Institutional Review Board, 3535 Seventh Avenue SW,Olympia, Washington 98502, United States.<br><br>Chairperson- Glenn C. Veit, JD, CIP
Hansen, Vincent. MD	033988/031414	Northern Utah Associates, 4403 Harrison Blvd., Suite 1685, Ogden, Utah 84403, United States.	Copernicus Group IRB, One Triangle Drive Suite 100, PO box 110605, Research Triangle Park, North Carolina 27709, United States.

Investigator	Investigator no./Center no.	Description of Research Facility, Hospital/ Institution, and Address	Name of IEC/IRB Committee, Address, Committee Chair
			Chairperson- Glenn C. Veit, JD, CIP
Hargis, Jeffrey. MD	116315/039044	Jewish Cancer Care, 2401 Terra Crossing Boulevard, Louisville, Kentucky 40245, United States.	Copernicus Group IRB, One Triangle Drive, Suite 100 PO Box 110605, Research Triangle Park, North Carolina 27709 United States.<br><br>Chairperson- Glenn C. Veit, JD, CIP
Hassany, Syed Fuad. MD.	107647/035457	Syed Fuad Hassany, M.D 1410 McFarland Boulevard. N Tuscaloosa, Alabama 35406, United States.	Copernicus Group IRB, One Triangle Drive, Suite 100, P.O. Box 11 0605, Research Triangle Park, North Carolina 27709, United States.<br><br>Chairperson- Glenn C. Veit, JD, CIP
Hellerstedt, Beth. MD	073401/031426	Texas Oncology Cancer Center, 6204 Balcones, Austin, Texas, 78731 United States.	US Oncology, Inc. Institutional Review Board, 10101 Woodloch Forest, The Woodlands, Texas, 77380, United States.<br><br>Chairperson- Elizabeth Rogg, M.D.

Investigator	Investigator no./Center no.	Description of Research Facility, Hospital/ Institution, and Address	Name of IEC/IRB Committee, Address, Committee Chair
Hendricks, Carolyn. MD	144278/051750	Suburban Hospital Cancer Program, 6420 Rockledge Drive, Suite 3900, Bethesda, Maryland 20817, United States.	Suburban Hospital IRB, 8600 Old Georgetown Road, Bethesda, Maryland 2081, United States.<br><br>Chairperson- Howard Lederman
Hermann, Robert. MD	017177/034980	Northwest Georgia Oncology Centers, PC, 340 Kennestone Hospital Blvd., Suite 200, Marietta, Georgia 30060, United States.	Western Institutional Review Board, 3535 Seventh Avenue SW, Olympia, Washington 98502, United States.<br><br>Chairperson- Theodore D. Schultz.
Herrada, Juan. MD	058154/039042	Texas Oncology-El Paso, Cancer Treatment Centre Grandview, 1901 Grandview, El Paso, Texas 79902, United States.	US Oncology, Inc. Institutional Review Board, 10101 Wood loch Forest, The Woodlands, Texas 77380, United Statse.<br>Chairperson- Elizabeth Rogg, M.D.
Holmes, Frankie Ann. MD	076818/033092	Frankie Ann Holmes, MD, Texas Oncology-Memorial City, 925 Gessner, Suite 100 Houston, Texas 77024, United States.	US Oncology, Inc. Institutional Review Board, 10101 Woodloch Forest, The Woodlands, Texas 77380, United States.

Investigator	Investigator no./Center no.	Description of Research Facility, Hospital/ Institution, and Address	Name of IEC/IRB Committee, Address, Committee Chair
			Chairperson- Elizabeth Rogg, M.D.
Iannotti, Nicholas. MD	027246/031883	Hematology/Oncology Associates of the Treasure Coast, 1871 SE Tiffany Avenue, Suite 100, Port Saint Lucie, Florida 34952, United States.	Copernicus Group IRB, One Triangle Drive Suite 100, PO box 110605, Research Triangle Park, North Carolina 27709, United States.<br><br>Chairperson- Glenn C. Veit, JD, CIP.
Jones, Michael. MD	105684/034978	The Jones Clinic, PC; 7710 Wolf River Circle, Germantown, Tennessee 38138, United States.	Copernicus Group IRB, 118 MacKenan Drive, Suite 400, Cary, North Carolina 27511, United States.<br><br>Chairperson- Glenn C. Veit, JD, CIP
Jones, Vicky. MD	042624/034088	Yakima Valley Memorial Hospital/North Star Lodge, 808 North 39th Avenue, Yakima, Washington 98902, United States.	US Oncology, Inc. Institutional Review Board, 10101 Woodloch Forest, The Woodlands, Texas 77380, United States.<br><br>Chairperson- Elizabeth Rogg, M.D.
Kahanic, Stephen. MD	031155/036660	Siouxland Hem-Onc Assoc. LLP, 230	Siouxland Institutional Review Board, Health,

Investigator	Investigator no./Center no.	Description of Research Facility, Hospital/ Institution, and Address	Name of IEC/IRB Committee, Address, Committee Chair
		Nebraska Street, Sioux City, Iowa 51101, United States.	Inc. 230 Nebraska Street, Sioux City, Iowa 51101, Untied States.<br><br>Chairperson- David Daniels, M.D.
Keaton, Mark. MD	081959/034489	Augusta Oncology Associates, 1348 Walton Way, Suite 4300, Augusta, Georgia 30901, United States.	Copernicus Group IRB, One Triangle Drive, Suite 100, PO Box 110605, Research Triangle Park, North Carolina 27709, United States.<br><br>Chairperson- Glenn C. Veit, JD, CIP
Kroener, Joan. MD	030025/036414	Scripps Clinic Torrey Pines, 10666 North Torrey Pines Road, La Jolla California 92037, United States.	Scripps IRB, 11025 North Torrey Pines Road, Suite 200,La Jolla, Carolina 92037, United States.<br><br>Chairperson- FWA#: 00007338
Kuebler, J Philip. MD	099875/032546	Columbus Oncology / Hematology Associates, Inc 810 Jasonway Avenue, Suite A Columbus, Ohio 43214, United States.	Coiumbus CCOP IRB, 1335 Dublin Road, Suite 124-A, Columbus, Ohio 43215, United States.

Investigator	Investigator no./Center no.	Description of Research Facility, Hospital/ Institution, and Address	Name of IEC/IRB Committee, Address, Committee Chair
			Chairperson- Jane Leiby M.D. and Thomas Anderson D.O., FACRO.
Letzer, Jeffrey.DO	101519/033499	Kalamazoo Hematology and Oncology, 1634 Gull Road, Suite 103 Kalamazoo, Michigan 49048, United States.	US Oncology, Inc. Institutional Review Board, 10101 Woodloch Forest, The Woodlands, Texas 77380, United States.<br><br>Chairperson- Elizabeth Rogg, M.D.
Lindquist, Deborah. MD	057928/031427	Arizona Oncology Associates, PC-NAHOA, 3700 W State Route 89A, Sedona, Arizona, 86336 United States.	US Oncology, Inc. Institutional Review Board, 10101 Woodloch Forest, The Woodlands, Texas 77380, United States.<br><br>Chairperson- Elizabeth Rogg, M.D.
Liu, Minetta. MD	057371/032176	Georgetown University Hospital, 3800 Reservoir Road, North West, Washington DC 20007, United States.	MedStar Research Institute-Georgetown University Oncology Institutional Review Board, Medical Dental Building, SW 104<br>3900 Reservoir Road, NW<br>Washington, DC 20057-2197, United States.

Investigator	Investigator no./Center no.	Description of Research Facility, Hospital/ Institution, and Address	Name of IEC/IRB Committee, Address, Committee Chair
			Chairperson- Jimmy Hwang.
Lowenthal, Ivan. MD<br>Overmoyer, Beth. MD (Former PI)	012092/032174	Connecticut Oncology & Hematology, LLP, 200 Kennedy Drive, Torrington, Connecticut 06790, United States.	Connecticut Oncology & Hematology, LLP, 200 Kennedy Drive, Torrington, Connecticut 06790, United States.<br><br>Chairperson- Elizabeth Rogg, M.D.
MacLaughlin, William. MD	106988/035279	Cancer Specialists of Tidewater, Ltd., 1 10 Wimbledon Square, Suite E, Chesapeake, Virginia 23320, United States.	Western Institutional Review Board, 3535 Seventh Avenue SW, Olympia, Washington 98502, United States.<br><br>Chairperson- Theodore D. Schultz.
Malamud, Stephen. MD	100118/032645	Beth Israel Medical Center, 10 Union Square East, New York, New York 10003, United States.	Institutional Review Board, Beth Israel Medical Center, 160 Water Street, 24th Floor, New York, 10038, United States.<br><br>Chairperson- Arnold Winston, M.D.
McGrath, James. MD	101800/033501	Gaston Hematology & Oncology, 261 0 Aberdeen Boulevard, Gastonia North Carolina	Copernicus Group IRB, One Triangle Drive, Suite 100, P.O. Box 110605, Research

Investigator	Investigator no./Center no.	Description of Research Facility, Hospital/ Institution, and Address	Name of IEC/IRB Committee, Address, Committee Chair
		28054, United States.	Triangle Park, North Carolina 27709, United States.<br><br>Chairperson- Glenn C. Veit, JD, CIP.
McIntyre, Kristi. MD	058169/033729	Texas Oncology, 8220 Walnut Hill Lane Professional Bldg. 11, Suite 700 Dallas, Texas 7523 1, United States.	US Oncology, Inc. Institutional Review Board, 101 01 Woodloch Forest -<br>The Woodlands, Texas 77380, United States.<br><br>Chairperson- Elizabeth Rogg, M.D.
Mehrotra, Avanti MD<br>Larson, Timothy. MD (Former PI)<br>Nagargoje, Gauri. MD (Former PI)	219136/037630	Hubert H. Humphrey Cancer Center 3435 West Broadway, Suite 1135, Robbinsdale, Minnesota 55422, United States.	North Memorial Health Care, Institutional Review Board, 3300 Oakdale Avenue, North, Robbinsdale, Minnesota 55422, United States.<br><br>Chairperson- Gary D. Hanovich, M.D.
Middleman, Edward. MD, MPH	064371/035281	Dallas Oncology Consultants, PA, 310 E.	Copernicus Group IRB, One Triangle Drive,

Investigator	Investigator no./Center no.	Description of Research Facility, Hospital/ Institution, and Address	Name of IEC/IRB Committee, Address, Committee Chair
		Highway 67, Duncanviile, Texas 751 37, United States.	Suite 100, P.O. Box 110605, Research Triangle Park, North Carolina 27709, United States.<br><br>Chairperson- Glenn C. Veit, JD, CIP.
Modiano, Manuel. MD	027237/038773	ACRC/Arizona Clinical Research Center, Inc. 1825 North Kolb Road, Tucson, Arizona 85715, United State.	Biomedical Research Institute of America, 2525 Camino del Rio South, Suite 300, San Diego, California 92108, United States.<br><br>Chairperson- Holly Hunter Stull.
Morrow, Phuong Khanh. MD	189531/031881	The University of Texas, M.D. Anderson Cancer Center, 1515 Holcombe Boulevard Houston, Texas 77030-4009, United States.	The University of Texas M.D. Anderson Cancer Center, Institutional Review Board - Unit 198 1515 Holcom be Boulevard Houston, Texas 77030-4009, United States.<br><br>Chairperson- Susan O'Brian
Moy, Beverly. MD, MPH	043676/040255	Massachusetts General Hospital, Yawkey 8 Pharmacy, 32 Fruit Street Boston,	Institutional Review Board cia<br>Office for Protection of Research Subjects

Investigator	Investigator no./Center no.	Description of Research Facility, Hospital/ Institution, and Address	Name of IEC/IRB Committee, Address, Committee Chair
		Massachusettes 02114, United States.	(OPRS) 450 Brookline Avenue Boston, Massachusettes 02215, United States.<br><br>Chairperson- Stephen Sallan, MD and Richard Penson, MD
Moy, Beverly. MD, MPH	043676/040195	Massachusetts General Hospital, 55 Fruit Street, Boston, MA 02114, United States.	Institutional Review Board c/o, Office for Protection of Research Subjects (OPRS) 450 Brookline Avenue, Boston, MA 02215, United States.<br><br>Chairperson- Stephen Sallan, MD and Richard Penson, MD
Moy, Beverly. MD, MPH	043676/033500	Massachusetts General Hospital, 32 Fruit Street, Boston, MA 02114, United States.	Institutional Review Board c/o, Office for Protection of Research Subjects (OPRS) 450 Brookline Avenue, Boston, MA 02215, United States.<br><br>Chairperson- Stephen Sallan, MD and

Investigator	Investigator no./Center no.	Description of Research Facility, Hospital/ Institution, and Address	Name of IEC/IRB Committee, Address, Committee Chair
			Richard Penson, MD
Nikcevich, Daniel. MD, PhD	052325/032029	Duluth Clinic, 420 East 1st Street<br>Duluth, Minnesota 55805, United States.	Saint Mary's Duluth Clinic Health System, Institutional Review Board, 400 East Third Street, Duluth, Minnesota 55805, United States.<br><br>Chairperson- Charles Gessert, MD, MPH.
Oratz, Ruth. MD, FACP	093328/037632	Ruth Oratz MD, 345 East 37th Street, Suite 202, New York 10016, United States.	US Oncology, Inc. Institutional Review Board, 101 01 Woodloch Forest - The Woodlands, Texas 77380, United States.<br><br>Chairperson- Elizabeth Rogg, M.D.
Orlowski, Richard. MD	019980/032183	Carolina Oncology Specialist P.A, 2406 Century Place S.E., Hickory, North Carolina, 28602 United States.	US Oncology, Inc. Institutional Review Board, 10101 Woodloch Forest<br>The Woodlands, Texas 77380, United States.<br><br>Chairperson- Elizabeth Rogg, M.D.

Investigator	Investigator no./Center no.	Description of Research Facility, Hospital/ Institution, and Address	Name of IEC/IRB Committee, Address, Committee Chair
Page, Ray. DO, PhD	011212/031884	The Center for Cancer and Blood Disorders, 800 West Magnolia, Fort Worth, Texas 76104, United States.	Copernicus Group IRB, One Triangle Drive, Suite 100, Research Triangle Park, North Carolina 27709, United States.<br><br>Chairperson- Glenn C. Veit, JD, CIP.
Papish, Steven. MD	018053/031880	Hematology-Oncology Associates of Northern NJ, PA Carol G. Simon Cancer Center, 100 Madison Avenue, Morristown, New Jersy 07962, United States.	US Oncology, Inc. Institutional Review Board, 101 01 Woodloch Forest, The Woodlands, Texas 77380, United States.<br><br>Chairperson- Elizabeth Rogg, M.D.
Patel, Ravi. MD	007639/034090	Comprehensive Blood and Cancer Center, 6501 Truxtun Avenue, Bakersfield, California 93309, United States.	Copernicus Group IRB, One Triangle Drive, Suite 100, PO Box 1 10605, Research Triangle Park, North Carolina 27703, United States.<br><br>Chairperson: Glenn C. Veit, JD, CIP
Paul, Devchand. DO, PhD	080295/045509	Rocky Mountain Cancer Centers,<br>Suite 400, 4700 East Hale Parkway, Denver,	US Oncology. Inc.<br>Institutional Review Board,

Investigator	Investigator no./Center no.	Description of Research Facility, Hospital/ Institution, and Address	Name of IEC/IRB Committee, Address, Committee Chair
		Colorado 80220, United States.	10101 Woodloch Forest Drive,<br>The Woodlands, Texas 77380, United States.<br><br>Chairperson- Elizabeth Rogg, M.D.
Prow, Debra. MD<br>Merchant, Joseph. MD (Former PI)	130236/038869	McFarland Clinic, P.C 1215 Duff Avenue Ames, Iowa 5001 0-3014, United States.	Mary Greeley Medical Center IRB<br>11 11 Duff Avenue Ames, Iowa 5001 0, United States.<br><br>Chairperson- Dario Zaffarano, J.D.
Rakowski, Thomas. MD<br>Chinitz, Allen. MD (Former PI)	121042/035378	The Valley Hospital, Luckow Pavilion, One Valley Health Plaza<br>Paramus, New Jersy 07652, United States.	The Valley Hospital Institutional Review Board 223 N. Van Dien Avenue Ridgewood, New Jersy 07450 United States.<br><br>Chairperson- David Montgomery, MD
Reznikoff, Glen. MD<br>Dressler, Kenneth. MD (Former PI)	102760/031878	Medical Specialists of Fairfield<br>425 Post Road Fairfield, Connecticut 06824, United States.	Copernicus Group IRB, One Triangle Drive Suite 100, PO box 110605, Research Triangle Park, North Carolina 27709, United States.

Investigator	Investigator no./Center no.	Description of Research Facility, Hospital/ Institution, and Address	Name of IEC/IRB Committee, Address, Committee Chair
			Chairperson- Glenn C. Veit, JD, CIP
Richards, Donald. MD, PhD	058188/032177	Tyler Cancer Center 9 10 E. Houston Street, Suite, 100 Tyler, Texas 75702	US Oncology, Inc. Institutional Review Board, 10101 Woodloch Forest<br>The Woodlands, TX 77380.<br><br>Chairperson- Elizabeth Rogg, M.D.
Richards, Donald<br><br>Rigden, Jamie. MD	112920/037905	Heartand Hematology-Oncology Associates Inc. 2000 NE Vivion Road Ist Fl. Kansas City, Missouri 64118, United States.	Copernicus Group IRB<br>118 MacKenan Drive Suite 400<br>Cary, North Carolina 27511, United States<br>.<br>Chairperson- Glenn C. Veit, JD, CIP
Rinn, Kristine. MD	027240/032182	Swedish Cancer Institute<br>122 1 Madison Street Seattle, Washington 98 104, United States.	Western Institutional Review Board 3535 Seventh Avenue, SW<br>Olympia, Washington 98502, United States.<br><br>Chairperson- Theodore D. Schultz.
Robert, Nicholas. MD	061344/031428	Virginia Cancer Specialist PC, 8503 Arlington	US Oncology, Inc. Institutional Review Board,

Investigator	Investigator no./Center no.	Description of Research Facility, Hospital/ Institution, and Address	Name of IEC/IRB Committee, Address, Committee Chair
		Blvd. Suite 400, Fairfax, Virginia 22031, United States.	10101 Woodloch Forest, The Woodlands, Texas 77380, United States.Chairperson- Elizabeth Rogg, M.D.
Roberts, Michael. MD	078410/032178	Hematology Oncology Associates, 3330 North 2nd Street, Suite 400, Phoenix, AZ 85012	US Oncology, Inc. Institutional Review Board, 4144 N. Central Expressway, Suite 1250, Dallas, TX 75204.<br><br>Chairperson- Elizabeth Rogg, M.D.
Rovito, Marc. MD	098747/032184	Consultants in Medical Oncology and Hematology, 2100 Keystone Avenue, Suite 502, Drexel Hill, Pennsylvania 19026, United States.	Copernicus Group IRB, One Triangle Drive Suite 100, PO Box 1 10605<br>Research Triangle Park, North Carolina 27703, United States.<br><br>Chairperson- Glenn C. Veit, JD, CIP.
Rubin, Peter. MD	040392/032644	Moses H. Cone Regional Cancer Center 501 North Elam Avenue<br>Greensboro, North Carolina 27403, United	Copernicus Group IRB<br>One Triangle Drive Suite 100<br>PO Box 1 10605, Research Triangle Park,

Investigator	Investigator no./Center no.	Description of Research Facility, Hospital/ Institution, and Address	Name of IEC/IRB Committee, Address, Committee Chair
		States.	North Carolina 27703, United States.<br><br>Chairperson- Glenn C. Veit, JD, CIP
Rubin, Rene. MD	097448/031882	Rittenhouse Hematology/Oncology PC, 207 N. Broad Street 6th floor, Philadelphia, Pennsylvania 191 07, United States.	Copernicus Group IRB<br>One Triangle Drive, Suite 100 PO Box 11 0605, USA, Research Triangle Park, North Carolina 27703, United States.<br><br>Chairperson- Glenn C. Veit, JD, CIP
Samuel, Edward. MD, PhD	029933/036417	North Shore Hernatology/Oncology Associates<br>235 N. Belle Mead Road.<br>East Setauket, New York 1 1733, United States.	Copernicus Group IRB<br>One Triangle Drive, Suite 100<br>PO Box 11 0605, Research Triangle Park, North Carolina 27703, United States.<br><br>Chairperson- Glenn C. Veit, JD, CIP
Sanchez, Ines. MD	114392/038302	Texas Oncology- El Paso Cancer Treatment Center, 1901 Grandview Avenue, El Paso, Texas 79902, United States.	Copernicus Group IRB, One Triangle Drive, Suite 100,<br>PO Box 11 0605, Research Triangle Park,

Investigator	Investigator no./Center no.	Description of Research Facility, Hospital/ Institution, and Address	Name of IEC/IRB Committee, Address, Committee Chair
			North Carolina 27703, United States.<br><br>Chairperson- Glenn C. Veit, JD, CIP
Savin, Michael. MD	058196/033627	Texas Cancer Center at Medical City 7777 Forest Lane Bldg. D400 Dallas, Texas 75230-2510, United States.	US Oncology, Inc Institutional Review Board, 4 144 N Central Expressway, Suite 1250, Dallas, Texas 75204, United States.<br><br>Chairperson- Elizabeth Rogg, M.D.
Schlabach, Larry. MD	112767/037903	Erlanger Health System, 975 East Third Street, Chattanooga, Tennessee 37403, United States.	University of Tennessee College of Medicine Erlanger IRB<br>Suite 102 960 East Third Street Chattanooga, Tennessee 37403 United States.<br><br>Chairperson- Manoo Bhakta, M.D.
Schlossman, David. MD, PhD	058201/032179	Missouri Cancer Associates, 1705 E Broadway, Suite 100, Columbia, Missouri 65201, United States.	US Oncology, Inc. Institutional Review Board, 4144 North Central Expressway, Suite 1250, Dallas, Texas 75204, United States.

Investigator	Investigator no./Center no.	Description of Research Facility, Hospital/ Institution, and Address	Name of IEC/IRB Committee, Address, Committee Chair
			Chairperson- Elizabeth Rogg, M.D.
Schwartzberg, Lee. MD, FACP	014204/035375	The West Clinic, 100 N. Humphreys Blvd., Memphis, Tennessee 381 20, United States.	Western Institutional Review Board 3535 Seventh Avenue SW, Olympia, Washington 98502, United States.<br><br>Chairperson- Theodore D. Schultz.
Seiler Jr, Milton. MD	064375/034979	Hematology and Oncology Specialists, LLC, 4228 Houma Blvd. Suite 130, Metairie, Louisiana 70006, United States.	Copernicus Group IRB<br>One Triangle Drive, Suite 100<br>PO Box 1 10605, Research Triangle Park, North Carolina 27703, United States.<br><br>Chairperson- Glenn C. Veit, JD, CIP
Shimkus, Brian. MD	105685/034977	Austin Cancer Centers<br>11111 Research Baoulevard. Ste.450 Austin, Texas 78759, United States.	Copernicus Group IRB<br>One Triangle Drive Ste. 100<br>PO Box 110605, Research Triangle Park, North Carolina 27709 United States.<br><br>Chairperson- Glenn C. Veit, JD, CIP.

Investigator	Investigator no./Center no.	Description of Research Facility, Hospital/ Institution, and Address	Name of IEC/IRB Committee, Address, Committee Chair
Silverman, Paula. MD	067686/036418	University Hospitals Case Medical Center11100 Euclid Avenue, Cleveland, Ohio 441 06, United States.	Case Cancer IR8, 10900 Euclid Avenue Cleveland, Ohio 44106, United States.<br><br>Chairperson- Rachel Egler, M.D.
Sivarajan, Kulumani. MD<br>Modi, Sanjiv. MD (Former PI)	073734/033327	Joliet Oncology-Hematology Associates, Ltd.(Main) 2614 W. Jefferson Street, Joliet, Illinois 60435, United States.	Copernicus Group IRB, One Triangle Drive, Suite 100, P.O. Box 110605, Research Triangle Park, North Carolina 27709, United States.<br><br>Chairperson- Glenn C. Veit, JD, CIP
Sleckman, Bethany. MD<br>Borson, Rachel. MD (Former PI)	026670/032180	Saint Louis Cancer & Breast Institute, 6435 Chippewa Saint, Saint Louis, Missouri 63109, United States.	Saint John's Mercy Medical Center Institutional Review Board, 615 S. New Ballas Road, 81. Louis, Missouri 63141C, United States.<br><br>Chairperson- Donald York, Ph.D
Smith, David MD	058202/031415	Northwest Cancer Specialists, P.C. 210 SE 136th Avenue. Vancouver, Washington 98684,	US Oncology, Inc. Institutional Review Board, 101 01 Woodloch Forest, The Woodlands,

Investigator	Investigator no./Center no.	Description of Research Facility, Hospital/ Institution, and Address	Name of IEC/IRB Committee, Address, Committee Chair
		United States.	Texas 77380, United States.<br><br>Chairperson- Elizabeth Rogg.
Smith, Karen. MD, MPH<br>Verma, Nitin. MD (Former PI)<br>Aggrawal, Anita. DO, PhD (Former PI)	105945/035424	Washington Cancer Institute at Washington Hospital Center, 110 Irving Street. NW. Washington 20010, United States	MedStar Research Institute-Georgetown University Oncology, Institutional Review Board, 3900 Reservoir Road, N.W.<br>Med-Dent SW 104, Washington, 20057United States.<br><br>Chairperson- David J. Perry, MD
Swan Jr, Forrest. MD	026587/031431	Cancer Outreach Associates, PC, 104 Abingdon Place, Abingdon, Virginia 24211, United States.	Copernicus Group IRB, One Triangle Drive, Suite 100<br>PO Box 1 10605, Research Triangle Park, North Carolina 27703, United States.<br><br>Chairperson- Glenn C. Veit, JD, CIP
Thomas-Joshua, Gracy. MD<br>Weiner, Robert (Former PI)	120507/038772	Medical Specialists of the Palm Beaches, Inc.10301 Hagen Ranch Road, Suite B4	Copernicus Group IRB, One Triangle Drive, Suite 100, PO Box 1 10605, Research

Investigator	Investigator no./Center no.	Description of Research Facility, Hospital/ Institution, and Address	Name of IEC/IRB Committee, Address, Committee Chair
		Boynton Beach, Florida 33437,United States.	Triangle Park, North Carolina 27703, United States.<br><br>Chairperson- Glenn C. Veit, JD, CIP
Van Haelst, Carol. MD	114380/038291	Cascade Cancer Center<br>12303 NE 130th L ane, Suite 120<br>Kirkland, Washington 98034, United States.	Copernicus Group IRB, One Triangle Drive, Suite 100, PO Box 1 10605, Research Triangle Park, North Carolina 27703, United States.<br><br>Chairperson- Glenn C. Veit, JD, CIP
Vogel, Charles. MD	014693/034582	Boca Raton Comprehensive Cancer Center, 21020 State Road 7 Boca Raton, Florida 33428, Untied States.	Chesapeake Research Review, Inc. 7063 Columbia Gateway Drive, Suite 110, Columbia, Maryland - 21046-3403, United States.<br><br>Chairperson- Anita Tarzian Ph. D
Vrindavanam, Nandagopal. MD	076598/033625	Signal Point Hematology/Oncology, Inc.<br>235 N. Breiel Boulevard, Middletown, Ohio	Copernicus Group IRB, One Triangle Drive Suite 100, PO Box 1 10605, Research

Investigator	Investigator no./Center no.	Description of Research Facility, Hospital/ Institution, and Address	Name of IEC/IRB Committee, Address, Committee Chair
		45042, United States.	Triangle Park, North Carolina 27703, United States.<br><br>Chairperson- Glenn C. Veit, JD, CIP
Weckstein, Douglas. MD	015518/033623	NH Oncology-Hematology, PA, 200 Technology Drive, Hooksett, New Hampshire 031 06, United States.	US Oncology, Inc. Institutional Review Board, 101 01 Woodloch Forest, The Woodlands, Texas 77380, United States.<br><br>Chairperson- Elizabeth Rogg, M.D.
Weigand, Robert. MD	017016/032535	Saint Joseph Oncology, Inc., 902 North Riverside Road, Suite 200, Saint Joseph, Missouri 64507, United States.	US Oncology, Inc. Institutional Review Board, 4144 N. Central Expressway, Suite 1250, Dallas, Texas 75204, United States.<br>Chairperson- Elizabeth Rogg, M.D.
Weigand, Robert<br>Weisberg, Tracey. MD	030049/031417	MCCM, 100 Campus Drive, Unit 108, Scarborough, Maine 04074, United States.	Copernicus Group IRB, One Triangle Drive Suite 100, P.O. Box 11 0605<br>Research Triangle Park, North Carolina 27709, United States.

Investigator	Investigator no./Center no.	Description of Research Facility, Hospital/ Institution, and Address	Name of IEC/IRB Committee, Address, Committee Chair
			Chairperson- Glenn C. Veit, JD, CIP
White Jr, Leonard. MD<br>Eckardt, John (Former PI)	037728/032532	Arch Medical Services, Inc. DBA The Center For Cancer Care and Research, 12855 North Forty Drive, Suite 200, St Louis, Missouri 63141, United States.	US Oncology, Inc. Institutional Review Board, 101 01 Woodloch Forest<br>The Woodlands, Texas 77380, United States.<br><br>Chairperson- Elizabeth Rogg, M.D.
Williams, Stephanie. MD	057038/035283	Hematology Oncology Associates of Illinois, 676 N. Saint Clair, Suite 2140, Chicago, Illinois 6061 1, United States.	US Oncology, Inc. Institutional Review Board, 4144 North Central Expressway, Suite 1250<br>Dallas, Texas 75204, United States.<br><br>Chairperson- Elizabeth Rogg, M.D.
Wright, Gail. MD	073605/031432	Florida Cancer Institute - New Hope 7651 Medical Drive, Hudson, Florida 34667, United States.	US Oncology, Inc. Institutional Review Board, 10101 Woodloch Forest<br>The Woodlands, Texas 77380, United States.<br><br>Chairperson - Elizabeth Rogg, M.D.
Wu, Hillary. MD<br>Loesch, David. MD (Fomer PI)	094788/033326	Central Indiana Cancer Centers 1346 E. County Line Road Indianapolis, Indiana 46227,	US Oncology, Inc. Institutional Review Board, 10101 Woodloch Forest, The Woodlands,

Investigator	Investigator no./Center no.	Description of Research Facility, Hospital/ Institution, and Address	Name of IEC/IRB Committee, Address, Committee Chair
		United States.	Texas 77380, United States.<br><br>Chairperson- Elizabeth Rogg, M.D.
Yanagihara, Ronald. MD	062863/036715	Ronald H Yanagihara, MD<br>9360 No Name Uno<br>Suite 130 Gilroy, California 95020, United States.	Copernicus Group IRB, One Triangle Drive Suite 100, PO Box 1 10605, Research Triangle Park, North Carolina 27703.<br><br>Chairperson- Glenn C. Veit, JD, CIP
Zrada, Stephen. MD	100120/032646	The Center for Cancer and Hematologic Disease, 1930 E. Rte 70, Suite V-107, Cherry Hill, New Jersy 08003, United States.	Copernicus Group IRB, One Triangle Drive, Suite 100, PO Box 1 10605, Research Triangle Park, North Carolina 27703.<br><br>Chairperson- Glenn C. Veit, JD, CIP

*No Patients Randomised.

All centres participated in the study under the US IND.
